# Comprehensive analysis of cuproptosis-related long non-coding RNA signature and personalized therapeutic strategy of breast cancer patients

**DOI:** 10.3389/fonc.2022.1081089

**Published:** 2022-12-22

**Authors:** Qiaonan Guo, Pengjun Qiu, Kelun Pan, Jianqing Lin

**Affiliations:** Department of Breast and Thyroid Surgery, The Second Affiliated Hospital of Fujian Medical University, Quanzhou, China

**Keywords:** breast cancer (BC), cuproptosis, long noncoding RNA (lncRNA), prognostic signature, immunotherapy

## Abstract

**Background:**

Breast cancer (BC) is considered to be one of the primary causes of cancer deaths in women. Cuproptosis was suggested to play an important role in tumor proliferation and tumor immune microenvironment. Therefore, an investigation was conducted to identify the relationship between cuproptosis-related long non-coding RNAs (lncRNAs) and BC prognosis.

**Method:**

Based on The Cancer Genome Atlas (TCGA), nine cuproptosis-related lncRNAs were identified by Pearson’s analysis and Cox regression analysis to create a cuproptosis-related lncRNA signature. Subsequently, patients with BC were divided into high-risk and low-risk groups. The Kaplan–Meier curves and a time-dependent receiver operating characteristic (ROC) analysis were employed to elucidate the predictive capability of the signature. After that, the Kyoto Encyclopedia of Genes and Genomes (KEGG) pathway analysis was conducted by Gene Set Enrichment Analysis (GSEA), and the lncRNA–mRNA co-expression network was established by Cytoscape software. Furthermore, the ESTIMATE score was calculated, and the immune cell type component analysis was conducted. Eventually, immunotherapy response analysis was applied to identify the predictive power of cuproptosis-related lncRNAs to tumor immunotherapy response, including immune checkpoint gene expression levels, tumor mutational burden (TMB), and microsatellite instability (MSI).

**Results:**

Patients with BC in the low-risk groups showed better clinical outcomes. The KEGG pathways in the high-risk groups were mainly enriched in immune response and immune cell activation. Furthermore, the ESTIMATE scores were higher in the low-risk groups, and their immune cell infiltrations were dramatically different from those of the high-risk groups. The low-risk groups were shown to have higher infiltration levels of CD8+ T cells and TMB-high status, resulting in better response to immunotherapies.

**Conclusion:**

The findings of this study revealed that the nine-cuproptosis-related lncRNA risk score was an independent prognostic factor for BC. This signature was a potential predictor for BC immunotherapy response. What we found will provide novel insight into immunotherapeutic treatment strategies in BC.

## 1 Introduction

Breast cancer is considered to be one of the primary causes of cancer deaths in women (1). Based on the statistics from the SEER database (https://seer.cancer.gov/), breast cancer (BC) accounts for 15% of all new cancer cases and 7.1% of all cancer deaths in the United States in 2022. Notably, the 5-year relative survival rate for patients with BC was 90.6%, steadily rising each year from 2012 to 2018. Despite the rapid development of diagnostic and therapeutic approaches, different molecular subtypes of BC respond differently to treatment due to the highly heterogeneous nature of breast carcinoma. Hence, it is important to find novel therapeutic targets and reliable prognostic indicators to achieve individual precision treatment.

Recently, tumor metastasis and drug resistance in BC have attracted a great deal of academic attention, which associate with the tumor immune microenvironment. Long non-coding RNA (lncRNA) is one of the vital regulators in the immune system and plays different roles in certain stages of cancer immunity, for instance, antigen presentation, immune cell activation, and immune responses (2–4). According to previous studies, lncRNAs were identified as high -potential prognostic predictors and therapeutic targets for BC (5).

The energy required for cell proliferation and growth is derived from cellular metabolism, which is therefore the basis for all life activities (6). A wide range of complex metabolic enzymes may produce an abundance of small molecules of metabolites during cellular metabolism. Copper is a mineral nutrient involved in cell proliferation and death pathways (7). In recent years, cuproptosis has been identified as a novel mechanism of cell death mediated by intracellular free copper, which is different from pyroptosis, apoptosis, necroptosis, autophagy, and ferroptosis (8). Peter Tsvetkov and colleagues indicated that cuproptosis occurs by means of the direct binding of copper to lipoylated components of the tricarboxylic acid (TCA) cycle. This results in lipoylated protein aggregation and subsequent iron–sulfur cluster protein loss, which leads to proteotoxic stress and ultimately cell death (9). Several previous studies found that copper has a vital role in various malignant tumors, such as endometrial cancer (10), glioma (11), head and neck carcinoma (12), and triple-negative breast cancer (TNBC) (13). These findings give an insight that copper toxicity is possible to be applied as an anti-tumor therapy to certain tumor patients. Cuproptosis-sensitive patients with BC may benefit from copper ionophore treatment.

Three anti-cuproptosis genes (MTF1, GLS, and CDKN2A) and seven pro-cuproptosis genes (FDX1, LIAS, LIPT1, DLD, DLAT, PDHA1, and PDHB) were extracted from the paper published by Peter Tsvetkov and colleagues (9). In the current study, we explore the correlation between the cuproptosis-related lncRNAs and clinical outcomes of patients with BC. Based on The Cancer Genome Atlas (TCGA) (http://cancergenome.nih.gov/) database, the prognostic lncRNAs of patients with BC related to cuproptosis were identified and analyzed. Subsequently, a cuproptosis-related lncRNA signature was constructed with the potential ability to predict the prognosis in patients with BC.

## 2 Methods and materials

### 2.1 Workflow

**Figure 1:**
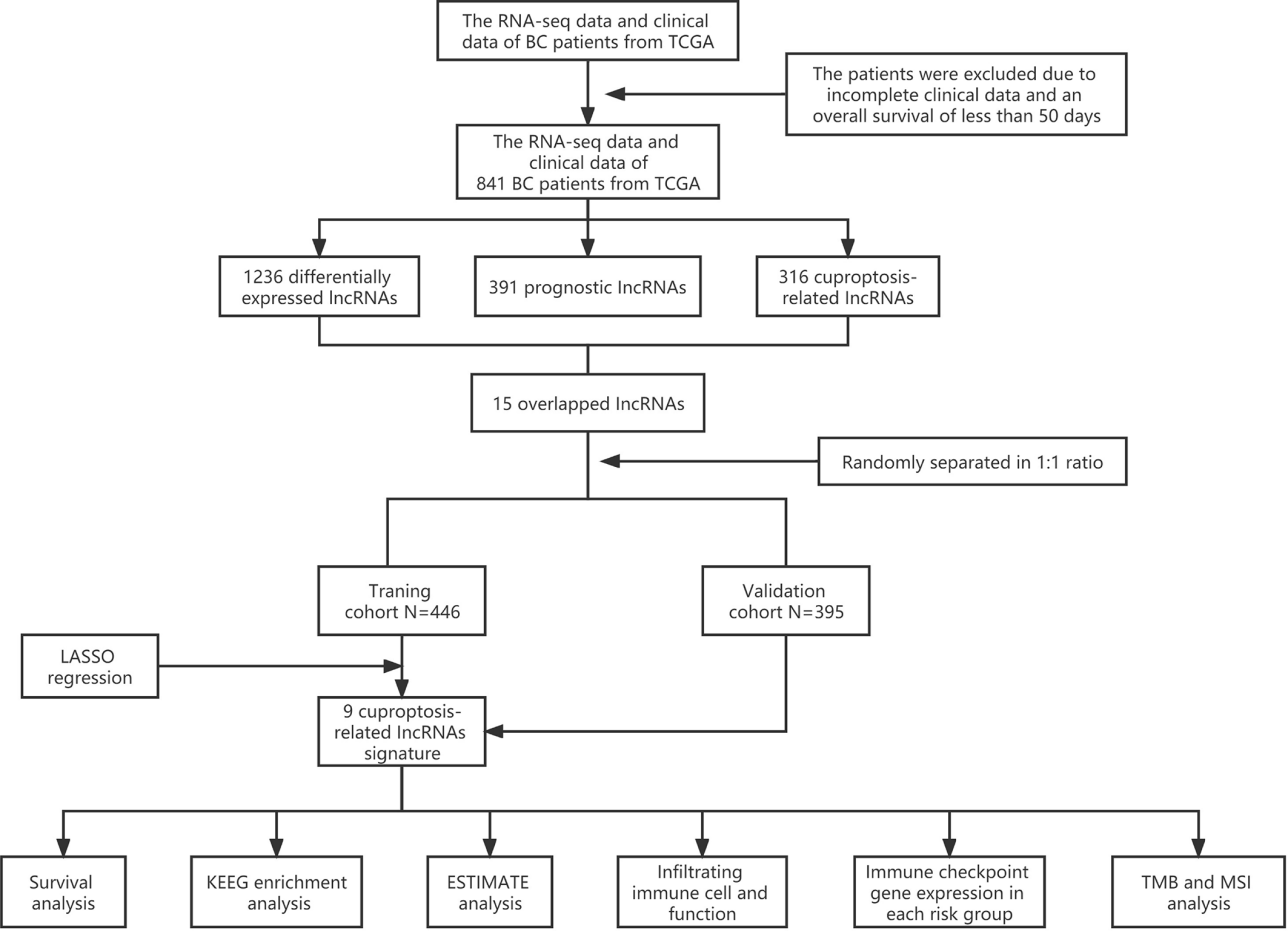
Analysis flow chart.

Sequential methods of several steps were used to construct a cuproptosis-related lncRNA signature and study the potential correlation between these cuproptosis-related lncRNAs and the clinical outcomes of patients with BC (**Figure 1**).

### 2.2 Data acquisition

RNA-sequencing expression data and corresponding clinical information of patients with BC were extracted from TCGA (http://cancergenome.nih.gov/) database, and subsequent batch normalization was carried out. Inclusion criteria were as follows: 1) BC samples with RNA-sequencing expression data and complete corresponding information and 2) BC samples with more than 50 days of follow-up. Exclusion criteria were as follows: 1) BC samples with incomplete clinical data and 2) BC samples with overall survival (OS) time of less than 50 days. Consequently, 841 samples were recruited and randomly separated into training and validation cohorts at a 1:1 ratio for follow-up studies. The data from TCGA are publicly available, and this study followed TCGA data access policies and publication guidelines. The 10 cuproptosis-related genes were extracted from the paper published by Peter Tsvetkov and colleagues (9).

### 2.3 Identification of cuproptosis-related lncRNAs

There are 3,158 lncRNAs included in our study, of which lncRNAs with a mean expression value of less than 1 were filtered out. After that, Pearson’s correlation analysis was employed to identify the cuproptosis-related lncRNAs based on the standard that p< 0.01 and |R| > 0.4.

### 2.4 Construction of a prognostic cuproptosis-associated lncRNA risk model

The differentially expressed lncRNAs were selected between breast tumor and normal samples by use of the “edge R” package and visualized *via* volcano plot. The cutoff criteria for differentially expressed lncRNAs were set as |log2fold change (FC)| > 1 and false discovery rate (FDR)- adjusted p< 0.05. Subsequently, the prognosis-associated lncRNAs were identified by univariate Cox regression analysis. The candidate lncRNAs were selected from the intersection of prognostic lncRNAs, cuproptosis-related lncRNAs, and differentially expressed lncRNAs. After that, the least absolute shrinkage and selection operator (LASSO) Cox regression model was established to reduce redundant lncRNAs and avoid model over-fitting. As a result, nine optimal prognostic cuproptosis-related lncRNAs were selected to create the risk model. Based on this prognostic signature, the individual risk score was calculated by the normalized expression levels of cuproptosis-related lncRNAs and corresponding regression coefficients. The calculation formula is as follows: Risk score = ∑ (*Expi* ∗*Coei*)*ni* = 1 (*N* = 9 , *Expi* denotes the expression level for each lncRNA, and *Coei* denotes the corresponding Cox regression coefficient). Consequently, the patients with BC in the training and validation cohorts were divided into the high-risk and low-risk groups based on the median risk score of the training cohort. Subsequently, the survival analysis and time-dependent receiver operating characteristic (ROC) curve analysis were conducted to evaluate the forecast accuracy of this prognostic signature. Finally, the principal component analysis (PCA) and t-distributed stochastic neighbor embedding (t-SNE) analysis were conducted based on the risk model by using the “prcomp” function of “stats” and “Rtsne” packages in R software.

### 2.5 Validation of the cuproptosis-associated lncRNA signature by clinicopathological characteristics

In order to identify the independent factor associated with the prognosis of patients with BC, the univariate Cox regression and multivariate Cox regression analysis were conducted among clinicopathological characteristics and the risk score on the basis of the cuproptosis-associated lncRNA signature.

### 2.6 Establishment of the lncRNA–mRNA co-expression network and functional enrichment analysis

The mRNA–lncRNA co-expression network was established by Cytoscape software to identify the correlation between cuproptosis-associated lncRNAs and the corresponding mRNAs, which was visualized by the Sankey diagram. The Gene Ontology (GO) functional enrichment analysis and two pathway analyses (Kyoto Encyclopedia of Genes and Genomes (KEGG) and REACTOME analyses) were performed between the high-risk and low-risk groups by use of Gene Set Enrichment Analysis (GSEA) (version 4.1.0, p< 0.05, FDR< 0.25).

### 2.7 Relevance assessment of risk score and tumor immune environment (TIME) characterization

Estimation of Stromal and Immune cells in Malignant Tumor tissues using expression (ESTIMATE) algorithm was applied to access the proportion of the immune-stromal component in TIME by using the “estimate R package”. Stromal Score, Immune Score, and ESTIMATE Score were calculated to imply the ratios of the corresponding compositions in the TIME.

### 2.8 Immune cell type component analysis

CIBERSORT (http://cibersort.stanford.edu/) was employed to calculate the proportion of 22 marked immune cell subtypes in the high- and low- risk groups by RNA-seq expression profile, of which the annotated gene expression features were visualized by LM22. Subsequently, the fraction of tumor- infiltrating immune cell (TIIC) type components in each sample was calculated, and the features of TIIC among different risk groups were distinguished by Wilcoxon’s test. p< 0.05 was considered statistically significant.

### 2.9 Immunotherapy response analysis

The correlation between the cuproptosis-related lncRNA risk score and the expression levels of genes related to immune checkpoint were analyzed by application of “ggplot2, GGPUBR, and ggExtra R packages”. Moreover, the “maftools R package” was employed to analyze the tumor mutational burden (TMB), which was defined as the number of somatic mutations per-mega base of the genomic sequence. Additionally, the transcription expression levels of critical mismatch repair (MMR) genes were counted and analyzed in the high-risk and low-risk groups, including MSH2, MSH6, MLH1, and PMS2. p< 0.05 was considered statistically significant.

### 2.10 Statistical analysis

All statistical analyses were performed *via* R software (version 4.1.0) (https://www.r-project.org/). The relevance of cuproptosis-related genes and corresponding lncRNAs was analyzed by Pearson’s correlation analysis. The categorical variables were analyzed by chi-square or Fisher’s test, whereas the continuous data were analyzed by the Wilcoxon test. The Kaplan–Meier curve was employed to assess the survival data. The univariate and multivariate Cox regression analyses were applied to estimate the independent prognostic elements. p< 0.05 was regarded as statistically significant. All methods were carried out in accordance with relevant guidelines and regulations.

## 3 Results

### 3.1 Data acquisition and processing

**Figure 2:**
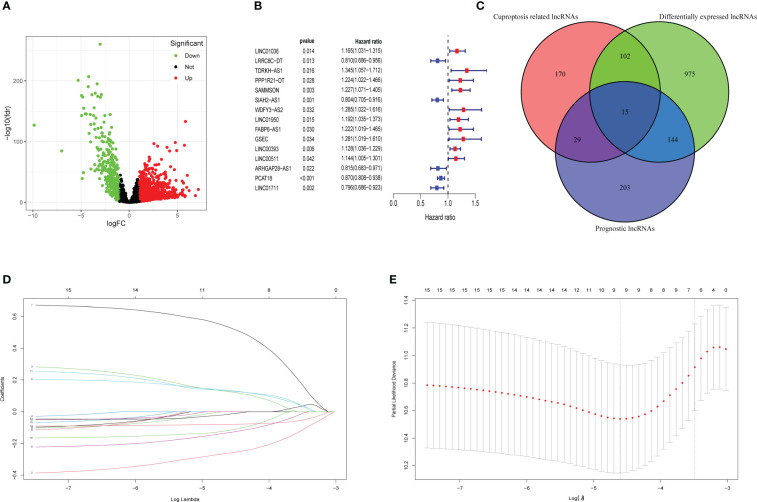
Collection of prognostic differential expression cuproptosis-related lncRNAs. **(A)** Volcano plot of differentially expressed lncRNAs. The downregulated lncRNAs are indicated by green spots, while the upregulated ones are indicated by red spots. **(B)** The HR (95% CI) and p-value of 15 collected prognosis- related lncRNAs. **(C)** The intersections of differentially expressed lncRNAs, prognosis-related lncRNAs, and cuproptosis-related lncRNAs were selected as the candidates. **(D)** LASSO coefficient profiles of 15 lncRNAs with p< 0.01. **(E)** Fivefold cross-validation results identified the optimal value of the penalty parameter λ. LASSO, least absolute shrinkage and selection operator; lncRNA, long non-coding RNA.

A total of 3,158 lncRNAs were selected from 112 normal mammary tissues and 841 BC tissues through RNA-seq data analysis. Subsequently, the basic characteristic information of patients with BC in the training cohort and validation cohort was downloaded from TCGA database and shown in **Table 1**. Ten cuproptosis-related genes (MTF1, GLS, CDKN2A, FDX1, LIAS, LIPT1, DLD, DLAT, PDHA1, and PDHB) were extracted from previous publications and ascertained in TCGA database. Moreover, the expression levels of 316 lncRNAs were identified as correlated with cuproptosis by Pearson’s correlation analysis (**Supplementary Table 1**). After that, 1,236 differentially expressed lncRNAs were identified and presented in the volcano map, of which 340 were downregulated and 896 were upregulated (**Figure 2A**, **Supplementary Table 2**). Then, 391 lncRNAs associated with the prognosis of patients with BC were screened out and provided in **Supplementary Table 3**, of which 15 candidate lncRNAs were shown in **Figure 2B**. The intersections of differentially expressed lncRNAs, cuproptosis-related lncRNAs, and prognostic lncRNAs were selected as candidate lncRNAs by the Venn diagram for LASSO regression analysis (**Figure 2C**). The LASSO coefficient profiles of the 15 lncRNAs were provided (**Figure 2D**), and fivefold cross-validation results were generated to confirm the best values of the penalty parameter λ (λ = 0.01001032) (**Figure 2E**). Consequently, nine cuproptosis-associated lncRNAs were obtained to construct the risk model: LRRC8C-DT, TDRKH-AS1, SAMMSON, SIAH2-AS1, WDFY3-AS2, LINC00393, ARHGAP28-AS1, PCAT18, and LINC01711. The expression levels of the selected nine lncRNAs in different tumor sizes and tumor stages (American Joint Committee on Cancer (AJCC) stages) were analyzed in patients with BC (**Supplementary Figure 1**).

**Table 1:** Clinicopathological features of patients with BC in training and validation groups.

Variables	Training cohort (n = 446)		Validation cohort (n = 395)		P-value
	No.	%	No.	%	
**Survival**	–	–	–	–	0.399
Alive	394	88.3	357	90. 4	–
Dead	52	11.7	38	9.6	–
**Age**	–	–	–	–	0.112
<=60	240	53.8	235	59.5	–
>60	206	46.2	160	40.5	–
**T**	–	–	–	–	0.401
T1	129	28.9	105	26.6	–
T2	241	54.0	232	58.7	–
T3	59	13.2	49	12.4	–
T4	17	3.8	9	2.3	–
**N**	–	–	–	–	0.709
N0	198	44.4	191	48.4	–
N1	160	35.9	137	34.7	–
N2	51	11.4	36	9.1	–
N3	32	7.2	28	7.1	–
Nx	5	1.1	3	0.8	–
**M**	–	–	–	–	0.496
M0	368	82.5	336	85.1	–
M1	9	2.0	9	2.3	–
Mx	69	15.5	50	12.7	–
**Stage**	–	–	–	–	0.317
Stage I	82	18.4	75	19.0	–
Stage II	243	54.5	233	59.0	–
Stage III	112	25.1	78	19.7	–
Stage IV	9	2.0	9	2.3	–

BC, breast cancer.

### 3.2 Construction of cuproptosis-related lncRNA risk model

**Figure 3:**
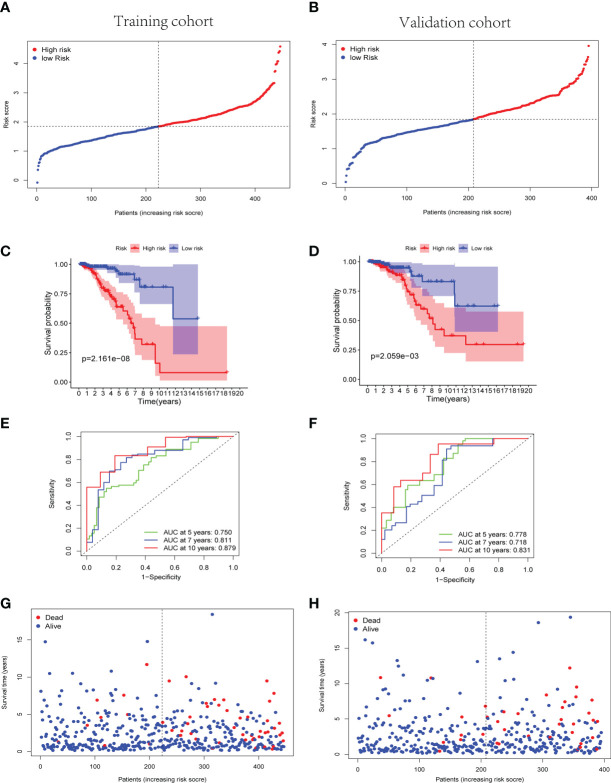
Predictive effectiveness evaluation of the cuproptosis-related lncRNA signature. **(A, B)** The distribution and median risk score in training group **(A)** and validation group **(B)**; the cutoff value of high- and low- risk sets was set as the median risk score of training cohort. **(C, D)** Kaplan–Meier survival curves for training **(C)** and validation **(D)** groups suggested that the OS of the high-risk sets was lower than that of the low-risk sets (p = 2.161E−08 and p = 2.059E−03). **(E, F)** ROC curve analysis for the accuracy of the risk model to forecast clinical outcomes of patients with BC at 5, 7, and 10 years in training **(E)** and validation **(F)** groups. **(G, H)** The distributions of survival time status in training **(G)** and validation **(H)** sets. lncRNA, long non-coding RNA; OS, overall survival; ROC, receiver operating characteristic; BC, breast cancer.

The cuproptosis-associated lncRNA signature was constructed based on the expression of the nine selected lncRNAs and their regression coefficients, as follows: risk score = (−0.261 × expression level of LRRC8C-DT) + (0.126 × expression level of TDRKH-AS1) + (0.135 × expression level of SAMMSON) + (−0.132 × expression level of SIAH2-AS1) + (0.533 × expression level of WDFY3-AS2) + (0.131 × expression level of LINC00393) + (−0.016 × expression level of ARHGAP28-AS1) + (−0.080 × expression level of PCAT18) + (−0.118 × expression level of LINC01711). Accordingly, the patients with BC in both the training and validation cohorts were divided into the high-risk and low-risk groups based on the median risk score of the training cohort (**Figures 3A, B**).

Subsequently, the Kaplan–Meier curves suggested better OS of the low-risk group patients with BC in training, validation, and whole TCGA cohorts (p< 0.01) (**Figures 3C, D** and **Supplementary Figure 2C**). Simultaneously, the Kaplan–Meier curves of the progress-free survival (PFS) in the training and validation cohorts were provided in **Supplementary Figure 3**. In the training cohort, a time-dependent ROC analysis indicated that the prognostic risk model was promising and efficient to predict the prognosis of patients with BC *via* the area under the curve (AUC) (AUC = 0.750, 0.811, and 0.879 at 5, 7, and 10 years, respectively, **Figure 3E**). Similarly, the time-dependent ROC analysis was applied in the validation cohort and the whole TCGA cohort to verify the robust predictive efficiency of the cuproptosis-related lncRNA signature (validation cohort, AUC = 0.778, 0.718, and 0.831 at 5, 7, and 10 years, respectively, **Figure 3F**; whole cohort, AUC = 0.759, 0.758, and 0.848 at 5, 7, and 10 years, respectively, **Supplementary Figure 2D**). Remarkably, patients with higher risk scores manifested a higher probability of death than those with lower risk scores (**Figures 3G, H**). The results of PCA and t-SNE analysis revealed that the patients with BC in different risk groups were broadly classified in the contrary directions (**Supplementary Figure 4**).

### 3.3 Identification of independent prognostic risk factors

**Figure 4:**
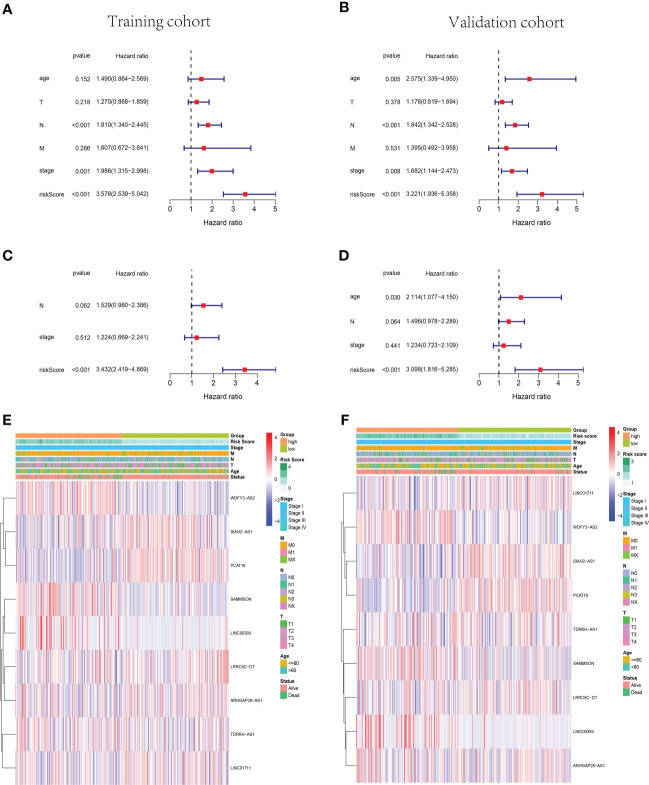
The cuproptosis-related lncRNA risk score was identified as an independent prognostic factor for patients with BC. Univariate and multivariate Cox regression analyses of the signature in the training **(A, C)** and validation **(B, D)** sets. Clustering analysis heatmaps indicate the expression levels of the nine identified cuproptosis-related lncRNAs and the clinicopathological characters of the respective patients with BC in training **(E)** and validation **(F)** groups (stage, AJCC tumor stage I/II/III/IV). lncRNA, long non-coding RNA; BC, breast cancer; AJCC, American Joint Committee on Cancer.

The univariate and multivariate Cox regression analyses were performed in the training cohort and validation cohort to identify the independent prognostic risk factors of patients with BC. In the training cohort, the univariate Cox regression analysis revealed that the lymph node status (N), AJCC stage, and risk score were independent prognostic factors (p< 0.001, HR = 1.810, 95% CI: 1.340–2.445; p = 0.001, HR = 1.986, 95% CI: 1.315–2.998; p< 0.001, HR = 3.578, 95% CI: 2.539–5.042, **Figure 4A**). In the validation cohort, not only were the N status, AJCC stage, and risk score independent risk factors but also age (p< 0.001, HR = 1.842, 95% CI: 1.342–2.528; p = 0.008, HR = 1.682, 95% CI: 1.144–2.473; p< 0.001, HR = 3.221, 95% CI: 1.936–5.358; p = 0.005, HR = 2.575, 95% CI: 1.339–4.950, **Figure 4B**). Moreover, the multivariate Cox regression analysis manifested that the risk score was an independent predictor for patients with BC in the training (p< 0.001, HR = 3.432, 95% CI: 2.419–4.869) and validation (p< 0.001, HR = 3.098, 95% CI: 1.816–5.285) cohorts (**Figures 4C, D**). Subsequently, the heatmaps were drawn to show the differences between the nine cuproptosis-related lncRNA expression levels and the clinical characteristics (age,<60/≥60 years; tumor stage, AJCC I/II/III/IV; vital status of patients; risk scores) in the training and validation groups (**Figures 4E, F**).

### 3.4 Construction of lncRNA–mRNA co-expression network and functional enrichment analysis

**Figure 5:**
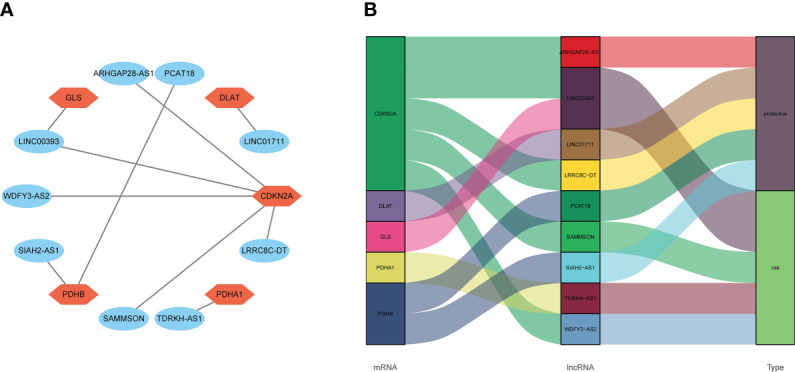
The relevance of cuproptosis lncRNAs and cuproptosis-related mRNAs. **(A)** Construction of a lncRNA–mRNA co-expression network. **(B)** The relationship among five cuproptosis-related mRNAs, nine cuproptosis-related lncRNAs, and the risk types (risk or protective). lncRNA, long non-coding RNA.

**Figure 6:**
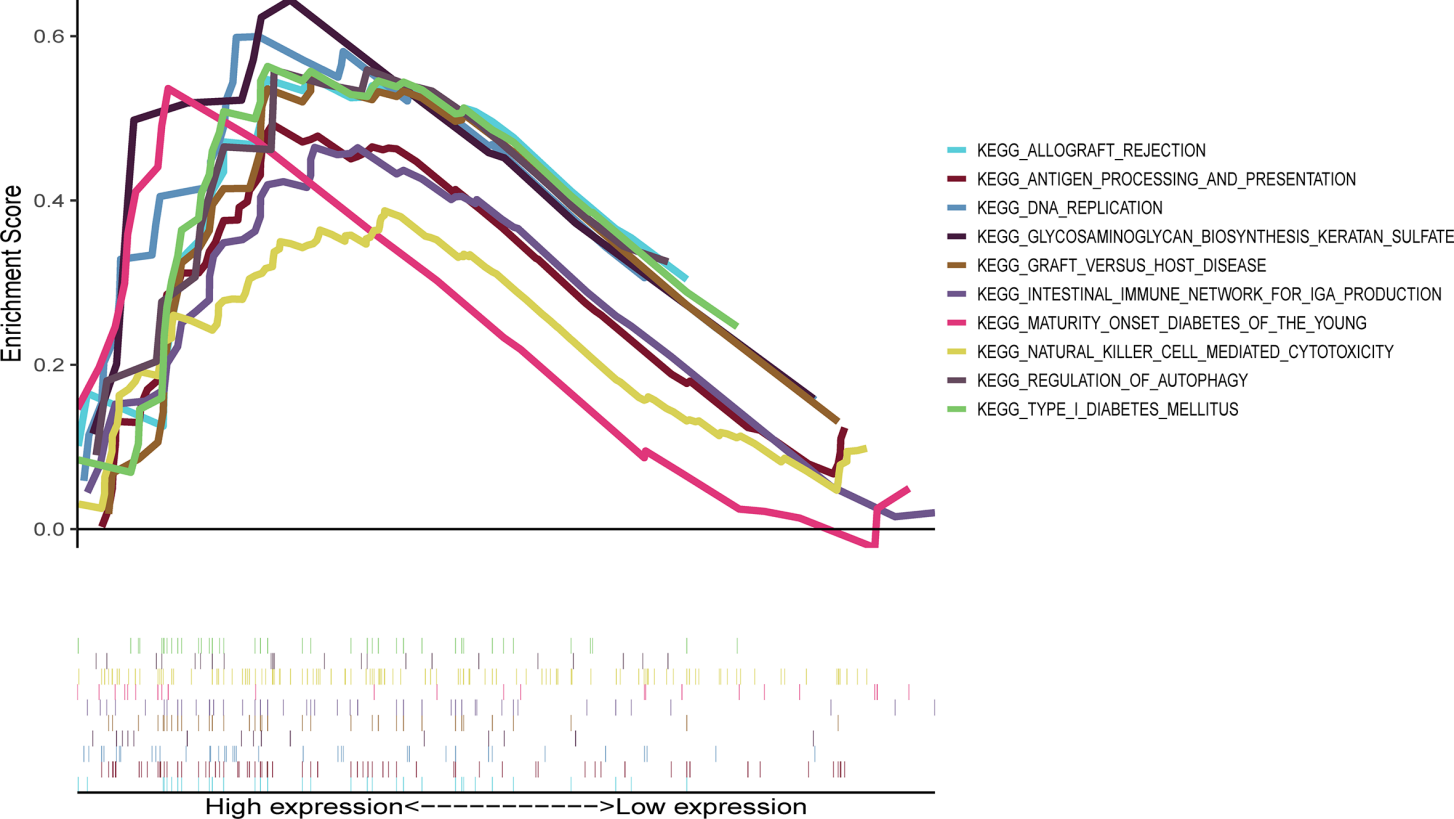
Representative results of KEGG enrichment analysis in the whole cohort. The KEGG analysis was conducted by GSEA, indicating that the top 10 active pathways in high-risk group were allograft rejection, antigen processing and presentation, DNA replication, glycosaminoglycan biosynthesis keratan sulfate, graft versus host disease, intestinal immune network for IgA production, maturity- onset diabetes of the young, natural killer cell- mediated cytotoxicity, regulation of autophagy, and type I diabetes mellitus. KEGG, Kyoto Encyclopedia of Genes and Genomes; GSEA, Gene Set Enrichment Analysis.

Ten lncRNA–mRNA pairs were enrolled in the lncRNA–mRNA co-expression network to further investigate the potential function of the nine cuproptosis-associated lncRNAs in breast malignant tumors (**Figure 5A**). The Sankey diagram showed not only the correlation between nine cuproptosis-related lncRNAs and targeted mRNAs but also the relevance between cuproptosis-related lncRNAs and risk types (**Figure 5B**). GSEA was performed to investigate the biological functions of the prognostic signature. In the KEGG analysis, the top 10 active pathways in the high-risk group were allograft rejection, antigen processing and presentation, DNA replication, glycosaminoglycan biosynthesis keratan sulfate, graft versus host disease, the intestinal immune network for IgA production, maturity- onset diabetes of the young, natural killer cell- mediated cytotoxicity, regulation of autophagy, and type I diabetes mellitus (**Figure 6**). The results of GO and REACTOME analyses are provided in **Supplementary Figure 5**.

### 3.5 The relationship between ESTIMATE score and cuproptosis-associated lncRNA signature

**Figure 7:**
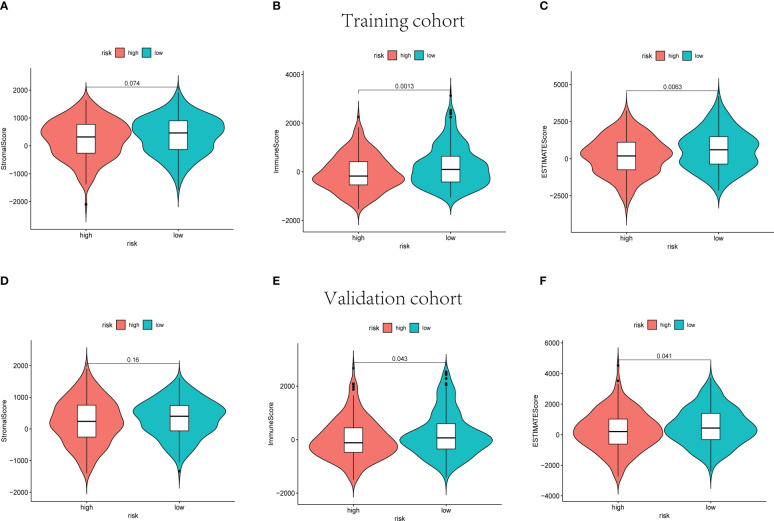
The stromal scores, immune scores, and ESTIMATE scores of high-risk and low-risk sets in training **(A–C)** and validation **(D–F)** groups.

The ESTIMATE score of each sample was calculated to investigate the tumor microenvironment (TME) landscape and the overall degree of immune infiltration. As a result, the high-risk groups showed lower immune and ESTIMATE scores than the low-risk groups in training, validation, and whole cohorts (p< 0.05, **Figure 7**, **Supplementary Figures 6A–C**).

### 3.6 Infiltrating immune cell distribution

**Figure 8:**
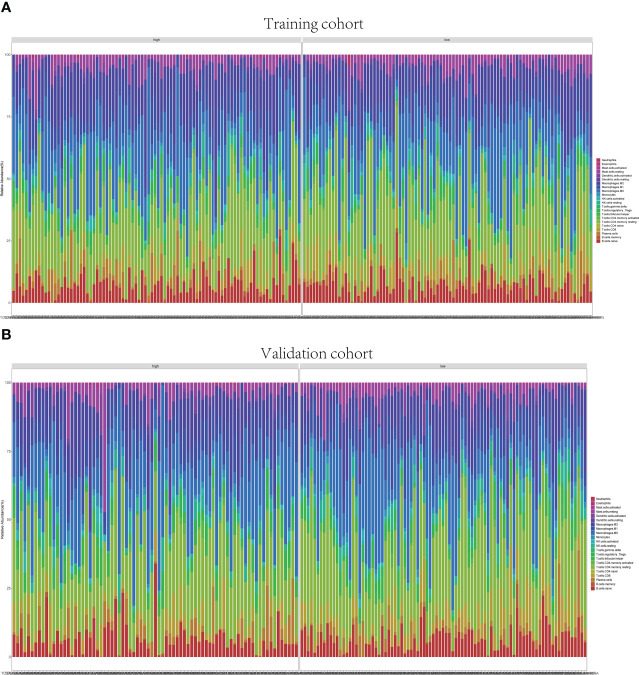
Immune infiltrations of training and validation groups. Relative proportion of immune infiltration in training group **(A)** and validation group **(B)**.

**Figure 9:**
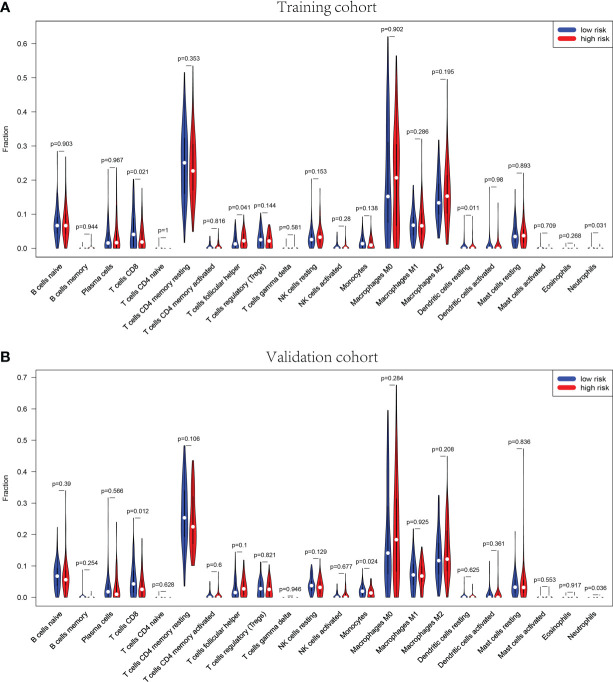
Correlation of distinct different immune cells between high- and low-risk sets in training **(A)** and validation **(B)** sets, p< 0.05. The high-risk sets are presented in red, and the low-risk sets are presented in blue.

The GSEA suggested that the immune cell activation and immune-related pathways were highly enriched in the high-risk group. Therefore, the TIIC proportions were calculated, and 22 kinds of TIIC profiles were established by the CIBERSORT algorithm (**Figure 8**). As shown in **Figure 9A**, CD8+ T cells (p = 0.021) and dendritic resting cells (p = 0.011) were downregulated while follicular help T cells (p = 0.041) were upregulated in the high-risk group of the training cohort. As shown in **Figure 9B**, CD8+ T cells (p = 0.012) and monocytes (p = 0.024) were downregulated in the high-risk group of the validation cohort. As shown in **Supplementary Figure 6D**, CD8+ T cells (p = 0.001) were downregulated in the high-risk group of the whole cohort. Consequently, the research on targeting cuproptosis-associated lncRNAs could be a groundbreaking discovery for the immunotherapy of cancer patients in the future.

### 3.7 Immunotherapy response prediction

**Figure 10:**
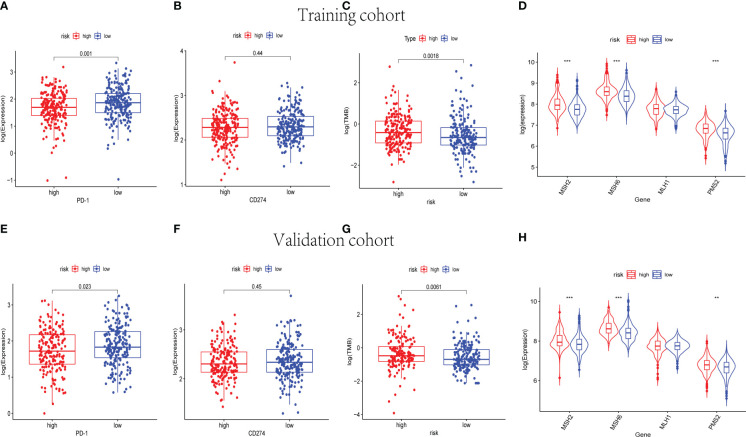
The immunotherapy response prediction analysis for the nine-cuproptosis-related lncRNA risk model. The expression levels of immune checkpoint- related genes PD-1 and PDL-1 in training **(A, B)** and validation **(E, F)** groups (p< 0.05). The differences in TMB between the high- and low- risk sets in training **(C)** and validation **(G)** groups (p< 0.05). The expression of MMR genes of BC samples in training **(D)** and validation **(H)** sets, including MSH2, MSH6, MLH1, and PMS2. TMB, tumor mutational burden; MMR, mismatch repair; BC, breast cancer. ** indicated P < 0.01 and *** indicated P < 0.001.

Immune checkpoint blockade (ICB) molecules, TMB, and MMR in neoplasm tissues were considered potential predictors for immunotherapy responses. The expression levels of ICB-related genes were estimated in the training and validation cohorts, including PD-1 and CD274 (**Figures 10A, B, E, F**). The expression level of PD-1 was upregulated in the low-risk groups of the training and validation cohorts (p< 0.05). As shown in **Figures 10C, G**, the low-risk groups were found significantly lower TMB in both the training and validation cohorts. Moreover, MSH2, MSH6, and PMS2 were observed to be expressed at low levels in the low-risk groups instead of the high-risk groups (p< 0.001) (**Figures 10D, H**). The results of the whole cohort were in line with those of the training and validation cohorts (**Supplementary Figures 6E–H**). Hence, based on the nine-cuproptosis-related lncRNA signature, the microsatellites were considered more stable in the high-risk group. The patients with lower risk scores were considered to respond better to immunotherapy.

## 4 Discussion

BC is a highly heterogeneous group of malignancies. Despite the development of multiple therapies, patients with breast carcinoma with distant metastases and drug resistance still have a high mortality rate (14). In recent years, more and more lncRNAs were identified as the biomarkers for BC early diagnosis and treatment. Ji Wang et al. found that lncRNA H19 in BC could induce autophagy activation to further contribute to tamoxifen resistance (15). In the study conducted by Laura and colleagues, lncRNA GATA3-AS1 was identified as associated with resistance to neoadjuvant chemotherapy in locally advanced patients with BC (16). In addition, immunotherapy for BC has received increasing attention, such as tumor-targeting antibodies, immune checkpoint blockade, adoptive T- cell therapy, and a combination of immunotherapies and conventional chemotherapies (17).

Cuproptosis is defined as a kind of regulated cell death that is distinct from other known death mechanisms. According to previous studies, cuproptosis takes place through the direct binding of copper to lipoylated components of the TCA cycle to further induce the lipoylated protein aggregation and iron–sulfur cluster protein loss, which results in proteotoxic stress and cell death (9, 18, 19). Previous reports have suggested that copper chelation is an effective form of therapy for Wilson’s disease, a genetic disorder of copper homeostasis (20). Ping Zhou et al. confirmed that copper chelation is an effective strategy for BC treatment by anti-angiogenesis. Thereby, copper chelation RPTDH/R848 nanoparticles were synthesized and applied as a therapeutic agent against metastatic BC *via* a combination of immune activation and anti-angiogenesis (21). In addition, copper- associated anti-tumor therapies were reported in several cancers, including gastric cancer (22), colorectal cancer (23, 24), and lung cancer (25). Therefore, copper ionophore treatment is possible to be a novel therapy for tumors.

In the current study, nine prognostic lncRNAs related to cuproptosis were identified to construct a risk model. According to the risk model, patients with BC were separated into the high-risk and low-risk groups. Notably, a pronounced distinction in prognosis between high- and low- risk sets was indicated by survival analysis. Patients with BC in the low-risk group were shown to have better OS. Subsequently, a lncRNA–mRNA co-expression network was established. ARHGAP28-AS1, LINC01711, LRRC8C-DT, PCAT18, and SIAH2-AS1 were shown as protective lncRNAs in patients with BC, whereas TDRKH-AS1, SAMMSON, WDFY3-AS2, and LINC00393 were identified as risk factors. Consistent with this observation, Zhang et al. found that PCAT18 was expressed at low levels in TNBC and could play a protective element in metastatic TNBC (26). Moreover, several previous studies have indicated that PCAT18 could inhibit the proliferation, migration, and invasion of gastric cancer (GC) cells, which could provide a theoretical basis for GC therapy (27–29). Also of interest, Xing et al. proposed that overexpression of SAMMSON could promote the proliferation of TNBC cells by interacting with p53 (30). Furthermore, the study conducted by Charlotte Orre and colleagues revealed the role of SAMMSON in the metabolic adaptations leading to the development of chemoresistance in BC cells (31). In some other studies, SAMMSON was identified as a key lncRNA in the progression of malignant tumors (32–34). In addition, WDFY3-AS2 was identified as a potential prognostic factor for patients with TNBC (35). TDRKH-AS1 was reported to promote the proliferation and invasion of colorectal cancer and hepatocellular carcinoma cells (36, 37).

Additionally, the KEGG analysis was applied and revealed that the high-risk groups were enriched in cellular metabolic pathways and immune cell activation. In the training and validation cohorts, the high-risk groups were shown lower immune and ESTIMATE scores. After that, the immune cell infiltration in different risk groups was analyzed. Of note, CD8+ T cells were obviously downregulated in the high-risk groups in addition to other immune cells. Subsequently, the immune checkpoint- related genes, MMR genes, and TMB were analyzed to detect the relationship between the cuproptosis-related lncRNA risk model and immunotherapy response. The expression level of PD-1 was upregulated in the low-risk groups. However, the TMB and the expression levels of MMR genes were indicated to be dramatically lower in the low-risk groups. Some previous reports suggested that a high infiltration ratio of CD8+ T cells was associated with enhanced sensitivity to ICB in breast carcinoma (38). These results were consistent with our observation. Therefore, patients with BC with lower cuproptosis-related lncRNA risk scores had higher rates of CD8+ T- cell infiltration, resulting in better responses to immunotherapies. Despite such mechanisms having yet to be validated *in vivo* and elucidated, the relevance among cuproptosis, immune responses, and TIME was significant for BC treatment in the future. Interestingly, our findings indicated that the high-risk groups have higher expression levels of MMR genes and TMB. Combined with previous reports, it is possible that patients with BC with higher cuproptosis-related lncRNA risk scores benefit from immunotherapies (39, 40). The immune checkpoint inhibitors (ICIs) related to gene PD-L1 early played a potential predictor of response to immunotherapy. However, various clinical trials have shed light on the limitations to regard PD-L1 expression as a predictive biomarker, namely, heterogeneous, dynamic, incapable of distinguishing adaptive and constitutive patterns of expression, and ignoring variant features of the TIME (41). By contrast, TMB-high status, despite varying from carcinoma types, is much more frequently encountered than PD-L1, expanding the potential population of patients eligible for ICIs (40–42). However, the results of the PD-L1 expression analysis were shown a statistically insignificant difference. In our study, the sample size included was not large enough, and the systematic bias caused by this may be one of the reasons for the statistically insignificant difference in PD-L1 expression results. Moreover, the association among the cuproptosis-related genes and PD-L1 is inconsistent. According to the previous study, PD-L1 expression was positively correlated with some cuproptosis-related genes (CDKN2A, FDX1, LIPT1, and MTF1) but negatively correlated with other cuproptosis-related genes (ATP7B, DLST, and PDHA1) (43). The molecular mechanisms between cuproptosis-related lncRNAs and PD-L1 were still unclear. The risk model established in this study was used to assess the prognosis of patients with BC by integrating the expression of cuproptosis-associated lncRNAs. Admittedly, this model is not sufficient for predicting treatment response to PD-L1 in patients with BC. This is a shortcoming of our study. Therefore, further research into the undying association between the CD274 expression and cuproptosis-related lncRNAs expression of BC is required. In addition, immune cell infiltration is associated with the response to immunotherapies. Some evidence suggested that PD-L1+ breast tumors had greater CD8+ T-cell infiltration than PD-L1 breast tumors (44). In the current study, CD8+ T-cell infiltration is significantly higher in the low-risk groups. Also, the expression level of PD-1 is significantly higher in the low-risk group. Combined with the results of microsatellite instability (MSI) analysis, the patients with BC in the low-risk groups were considered to respond better to immunotherapies.

To our knowledge, this is the first study to investigate the relevance among cuproptosis-related lncRNAs, TIME, and prognosis of patients with BC. However, there are also some limitations in the current study. Firstly, given that all the data to establish the prognostic risk model were extracted from a single public database TCGA, further biological mechanisms of cuproptosis-related lncRNAs are needed apart from the statistical evidence provided. Secondly, yet even though the nine cuproptosis-related lncRNAs showed good performance in predicting BC prognosis, there are still some other vital genes and lncRNAs with predictive values that were ignored in the current study. It is urgent to conduct experiments *in vivo* and *in vitro* to investigate the mechanisms of cuproptosis in BC and the interaction between cuproptosis-related lncRNAs and immune cell infiltrations. Collectively, the results of our study provide the foothold for exploring the predictive biomarkers for patients with BC, which may make contributions to elucidating the biological mechanisms of cuproptosis-related lncRNAs. In the future, the immunotherapy response prediction in BC may be enhanced by inferring cuproptosis features from the cuproptosis-related lncRNAs data.

## 5 Conclusion

The findings of our research provide a promising approach to facilitate the prediction of individualized survival in patients with BC and may be helpful to elucidate the mechanism of cuproptosis-related lncRNAs in BC progression. Moreover, the predictive model is beneficial in screening for clinical characteristics of patients with BC who respond better to immunotherapeutic treatments.

## Data availability statement

The original contributions presented in the study are included in the article/**Supplementary Material**. Further inquiries can be directed to the corresponding author.

## Ethics statement

The data of participants in this study were downloaded from TCGA database. TCGA belongs to public databases. Ethical approval was obtained from tThe patients involved in the database have obtained ethical approval (https://www.cancer.gov/about-nci/organization/ccg/research/structural-genomics/tcga).

## Author contributions

Acquisition of data (databases acquiring and data processing, etc.): QG and PQ; Analysis and interpretation of data (e.g., statistical analysis, biostatistics, computational analysis): QG, PQ, and KP; Writing, review, and/or revision of the manuscript: QG, PQ, and JL; Administrative, technical, or material support (i.e., reporting or organizing data, constructing databases): PQ and QG; Study supervision: JL. All authors contributed to the article and approved the submitted version.

## Supplementary material

Supplementary Figure 1 The expression levels of the 9 cuproptosis-related lncRNAs in different tumor size and tumor stages for patients with BC.

Supplementary Figure 2 The distribution and median risk score in whole cohort, and the cut-off value of high and low risk sets was set as the median risk score of training cohort **(A, B)**. Kaplan–Meier survival curves for whole cohort **(C)**, suggested that the OS of the high-risk sets was lower than that of the low-risk sets (P=8.061E-10). ROC curve analysis for the accuracy of the risk model to forecast clinical outcomes of patients with BC at 5, 7 and 10 years in the whole cohort **(D)**. The results of PCA **(E)** and tSNE analysis **(F)** in whole cohort.

Supplementary Figure 3 The Kaplan-Meier analysis of PFS in training and validation cohorts. Kaplan–Meier survival curves for training **(A)** and validation **(B)** groups indicated that the PFS of the high-risk sets was lower than that of the low-risk sets (P=1.774E-01 and P=8.528E-02).

Supplementary Figure 4 PCA plot and tSNE plot of the training and validation groups. The patients with high risk were denoted by red points, and the patients with low risk were denoted by blue points.

Supplementary Figure 5 Representative results of GO enrichment analysis and REACTOME pathway analysis in the whole cohort.

Supplementary Figure 6 The stromal scores, immune scores, and ESTIMATE scores of high-risk and low-risk sets in the whole cohort **(A–C)**. The correlation of distinct different immune cells between high- and low-risk sets in the whole cohort **(D)**, P<0.05. The high-risk sets are presented with red and the low-risk sets are presented with blue. The expression levels of immune checkpoint related genes PD-1 and PDL-1 in the whole cohort **(E, F)**. The differences of TMB between the high- and low- risk sets in the whole cohort **(G)**. The expression of MMR genes of BC samples in the whole cohort **(H)**.

## References

[1] Bray F Ferlay J Soerjomataram I Siegel R Torre L and Jemal A . Global cancer statistics 2018: Globocan estimates of incidence and mortality worldwide for 36 cancers in 185 countries. CA Cancer J Clin (2018) 68(6):394–424. doi: 10.3322/caac.21492 30207593

[2] Ma W Zhao F Yu X Guan S Suo H Tao Z . Immune-related lncrnas as predictors of survival in breast cancer: A prognostic signature. J Transl Med (2020) 18(1):442. doi: 10.1186/s12967-020-02522-6 33225954 PMC7681988

[3] Denaro N Merlano M Lo Nigro C . Long noncoding rnas as regulators of cancer immunity. Mol Oncol (2019) 13(1):61–73. doi: 10.1002/1878-0261.12413 30499165 PMC6322193

[4] Zhang L Xu X Su X . Noncoding rnas in cancer immunity: Functions, regulatory mechanisms, and clinical application. Mol Cancer (2020) 19(1):48. doi: 10.1186/s12943-020-01154-0 32122338 PMC7050126

[5] Kim J Piao H Kim B Yao F Han Z Wang Y . Long noncoding rna Malat1 suppresses breast cancer metastasis. Nat Genet (2018) 50(12):1705–15. doi: 10.1038/s41588-018-0252-3 PMC6265076 30349115

[6] Li L Goel A Wang X . Novel paradigms of mitochondrial biology and function: Potential clinical significance in the era of precision medicine. Cell Biol Toxicol (2022) 38(3):371–5. doi: 10.1007/s10565-022-09721-5 35618927

[7] Ruiz L Libedinsky A Elorza A . Role of copper on mitochondrial function and metabolism. Front Mol Biosci (2021) 8:711227. doi: 10.3389/fmolb.2021.711227 34504870 PMC8421569

[8] Ge E Bush A Casini A Cobine P Cross J DeNicola G . Connecting copper and cancer: From transition metal signalling to metalloplasia. Nat Rev Cancer (2022) 22(2):102–13. doi: 10.1038/s41568-021-00417-2 PMC8810673 34764459

[9] Tsvetkov P Coy S Petrova B Dreishpoon M Verma A Abdusamad M . Copper induces cell death by targeting lipoylated tca cycle proteins. Sci (New York NY) (2022) 375(6586):1254–61. doi: 10.1126/science.abf0529 PMC9273333 35298263

[10] Atakul T Altinkaya S Abas B Yenisey C . Serum copper and zinc levels in patients with endometrial cancer. Biol Trace Elem Res (2020) 195(1):46–54. doi: 10.1007/s12011-019-01844-x 31399869

[11] Li Y Fu S Wang L Wang F Wang N Cao Q . Copper improves the anti-angiogenic activity of disulfiram through the Egfr/Src/Vegf pathway in gliomas. Cancer Lett (2015) 369(1):86–96. doi: 10.1016/j.canlet.2015.07.029 26254539

[12] Ressnerova A Raudenska M Holubova M Svobodova M Polanska H Babula P . Zinc and copper homeostasis in head and neck cancer: Review and meta-analysis. Curr Med Chem (2016) 23(13):1304–30. doi: 10.2174/0929867323666160405111543 27048341

[13] Cui L Gouw A LaGory E Guo S Attarwala N Tang Y . Mitochondrial copper depletion suppresses triple-negative breast cancer in mice. Nat Biotechnol (2021) 39(3):357–67. doi: 10.1038/s41587-020-0707-9 PMC7956242 33077961

[14] Harbeck N Penault-Llorca F Cortes J Gnant M Houssami N Poortmans P . Breast cancer. Nat Rev Dis Primers (2019) 5(1):66. doi: 10.1038/s41572-019-0111-2 31548545

[15] Wang J Xie S Yang J Xiong H Jia Y Zhou Y . The long noncoding rna H19 promotes tamoxifen resistance in breast cancer *Via* autophagy. J Hematol Oncol (2019) 12(1):81. doi: 10.1186/s13045-019-0747-0 31340867 PMC6657081

[16] Contreras-Espinosa L Alcaraz N de la Rosa-Velázquez I Díaz-Chávez J Cabrera-Galeana P Rebollar-Vega R . Transcriptome analysis identifies Gata3-As1 as a long noncoding rna associated with resistance to neoadjuvant chemotherapy in locally advanced breast cancer patients. J Mol diagnostics JMD (2021) 23(10):1306–23. doi: 10.1016/j.jmoldx.2021.07.014 34358678

[17] Barzaman K Moradi-Kalbolandi S Hosseinzadeh A Kazemi M Khorramdelazad H Safari E . Breast cancer immunotherapy: Current and novel approaches. Int Immunopharmacol (2021) 98:107886. doi: 10.1016/j.intimp.2021.107886 34153663

[18] Oliveri V . Selective targeting of cancer cells by copper ionophores: An overview. Front Mol Biosci (2022) 9:841814. doi: 10.3389/fmolb.2022.841814 35309510 PMC8931543

[19] Tang D Chen X Kroemer G . Cuproptosis: A copper-triggered modality of mitochondrial cell death. Cell Res (2022) 32(5):417–8. doi: 10.1038/s41422-022-00653-7 PMC9061796 35354936

[20] Aggarwal A Bhatt M . Advances in treatment of Wilson disease. Tremor other hyperkinetic movements (New York NY) (2018) 8:525. doi: 10.7916/d841881d PMC5840318 29520330

[21] Zhou P Qin J Zhou C Wan G Liu Y Zhang M . Multifunctional nanoparticles based on a polymeric copper chelator for combination treatment of metastatic breast cancer. Biomaterials (2019) 195:86–99. doi: 10.1016/j.biomaterials.2019.01.007 30623789

[22] Liu Y Guan X Wang M Wang N Chen Y Li B . Disulfiram/Copper induces antitumor activity against gastric cancer *Via* the Ros/Mapk and Npl4 pathways. Bioengineered (2022) 13(3):6579–89. doi: 10.1080/21655979.2022.2038434 PMC9278967 35290151

[23] Aubert L Nandagopal N Steinhart Z Lavoie G Nourreddine S Berman J . Copper bioavailability is a kras-specific vulnerability in colorectal cancer. Nat Commun (2020) 11(1):3701. doi: 10.1038/s41467-020-17549-y 32709883 PMC7381612

[24] Al-Zharani M Qurtam A Daoush W Eisa M Aljarba N Alkahtani S . Antitumor effect of copper nanoparticles on human breast and colon malignancies. Environ Sci pollut Res Int (2021) 28(2):1587–95. doi: 10.1007/s11356-020-09843-5 32851522

[25] Tsang T Posimo J Gudiel A Cicchini M Feldser D Brady D . Copper is an essential regulator of the autophagic kinases Ulk1/2 to drive lung adenocarcinoma. Nat Cell Biol (2020) 22(4):412–24. doi: 10.1038/s41556-020-0481-4 PMC7610258 32203415

[26] Zhang J Liu D Deng G Wang Q Li L Zhang J . Lncrna prostate cancer-associated transcript 18 upregulates activating transcription factor 7 to prevent metastasis of triple-negative breast cancer *via* sponging mir-103a-3p. Bioengineered (2021) 12(2):12070–86. doi: 10.1080/21655979.2021.2003928 PMC8809992 34787047

[27] Zhu L Zhang C Xue J He X Yin D Zhu Q . Ezh2-mediated epigenetic suppression of lncrna Pcat18 predicts a poor prognosis and regulates the expression of P16 by interacting with mir-570a-3p in gastric cancer. J Cancer (2021) 12(23):7069–78. doi: 10.7150/jca.63415 PMC8558664 34729108

[28] Dou J Tu D Zhao H Zhang X . Lncrna Pcat18/Mir-301a/Tp53inp1 axis is involved in gastric cancer cell viability, migration and invasion. J Biochem (2020) 168(5):547–55. doi: 10.1093/jb/mvaa079 32687182

[29] Zhang X Mao H Zhang S Sun L Zhang W Chen Q . Lncrna Pcat18 inhibits proliferation, migration and invasion of gastric cancer cells through mir-135b suppression to promote Cldn11 expression. Life Sci (2020) 249:117478. doi: 10.1016/j.lfs.2020.117478 32119960

[30] Xing Z Zhang M Liu J Liu G Feng K Wang X . Overexpression of incrna sammson promotes triple-negative breast cancer cell proliferation by interacting with P53. Crit Rev Eukaryot Gene Expr (2021) 31(6):1–8. doi: 10.1615/CritRevEukaryotGeneExpr.2021039534 34936287

[31] Orre C Dieu X Guillon J Gueguen N Ahmadpour S Dumas J . The long non-coding rna sammson is a regulator of chemosensitivity and metabolic orientation in mcf-7 doxorubicin-resistant breast cancer cells. Biology (2021) 10(11):1156. doi: 10.3390/biology10111156 34827149 PMC8615054

[32] Ni H Wang K Xie P Zuo J Liu W Liu C . Lncrna sammson knockdown inhibits the malignancy of glioblastoma cells by inactivation of the Pi3k/Akt pathway. Cell Mol Neurobiol (2021) 41(1):79–90. doi: 10.1007/s10571-020-00833-2 32236901 PMC11448660

[33] Yang S Cai H Hu B Tu J . Lncrna sammson negatively regulates mir-9-3p in hepatocellular carcinoma cells and has prognostic values. Biosci Rep (2019) 39(7):BSR20190615. doi: 10.1042/bsr20190615 31164410 PMC6609599

[34] Sun S Lin S Cao H Xiao Z . Values of long noncoding rna sammson in the clinicopathologic features and the prognostic implications of human gastric cancer. Eur Rev Med Pharmacol Sci (2020) 24(11):6080–7. doi: 10.26355/eurrev_202006_21503 32572923

[35] Rodrigues de Bastos D Nagai M . In silico analyses identify lncrnas: Wdfy3-As2, bdnf-as and Afap1-As1 as potential prognostic factors for patients with triple-negative breast tumors. PloS One (2020) 15(5):e0232284. doi: 10.1371/journal.pone.0232284 32401758 PMC7219740

[36] Jiao Y Zhou J Jin Y Yang Y Song M Zhang L . Tdrkh-As1long non-coding rna promotes colorectal cancer cell proliferation and invasion through the B-catenin activated signaling pathway. Front Oncol (2020) 10:639. doi: 10.3389/fonc.2020.00639 32670860 PMC7326065

[37] Bu X Ma L Liu S Wen D Kan A Xu Y . A novel qualitative signature based on lncrna pairs for prognosis prediction in hepatocellular carcinoma. Cancer Cell Int (2022) 22(1):95. doi: 10.1186/s12935-022-02507-z 35193591 PMC8862507

[38] Jenkins L Jungwirth U Avgustinova A Iravani M Mills A Haider S . Cancer-associated fibroblasts suppress Cd8+ T cell infiltration and confer resistance to immune checkpoint blockade. Cancer Res (2022) 82(16):2904–17. doi: 10.1158/0008-5472.can-21-4141 PMC9379365 35749591

[39] Chumsri S Sokol E Soyano-Muller A Parrondo R Reynolds G Nassar A . Durable complete response with immune checkpoint inhibitor in breast cancer with high tumor mutational burden and apobec signature. J Natl Compr Cancer Network JNCCN (2020) 18(5):517–21. doi: 10.6004/jnccn.2020.7543 32380464

[40] Fan S Gao X Qin Q Li H Yuan Z Zhao S . Association between tumor mutation burden and immune infiltration in ovarian cancer. Int Immunopharmacol (2020) 89:107126. doi: 10.1016/j.intimp.2020.107126 33189611

[41] Passaro A Stenzinger A Peters S . Tumor mutational burden as a pan-cancer biomarker for immunotherapy: The limits and potential for convergence. Cancer Cell (2020) 38(5):624–5. doi: 10.1016/j.ccell.2020.10.019 33171127

[42] Zang Y Dai C Xu X Cai X Wang G Wei J . Comprehensive analysis of potential immunotherapy genomic biomarkers in 1000 Chinese patients with cancer. Cancer Med (2019) 8(10):4699–708. doi: 10.1002/cam4.2381 PMC6712454 31270941

[43] Lv H Liu X Zeng X Liu Y Zhang C Zhang Q . Comprehensive analysis of cuproptosis-related genes in immune infiltration and prognosis in melanoma. Front Pharmacol (2022) 13:930041. doi: 10.3389/fphar.2022.930041 35837286 PMC9273972

[44] Mittendorf EA Philips AV Meric-Bernstam F Qiao N Wu Y Harrington S . Pd-L1 expression in triple-negative breast cancer. Cancer Immunol Res (2014) 2(4):361–70. doi: 10.1158/2326-6066.CIR-13-0127 PMC4000553 24764583

