# Supplementary material for: Comprehensive analysis of cuproptosis-related long non-coding RNA signature and personalized therapeutic strategy of breast cancer patients
Source: Front Oncol. 2022 Dec 22;12:1081089. doi: 10.3389/fonc.2022.1081089 (PMC9815178; doi:10.3389/fonc.2022.1081089)
Supplement: Supplementary file 1 [file DataSheet_1.pdf]

id	B cells naive	B cells memory	Plasma cells	T cells CD8	T cells CD4 naive
TCGA-A2-A259	0.1306584	0	0	0.01249958	0
TCGA-LL-A5YL	0.1206136	0	0.00623823	0.09991668	0
TCGA-AC-A6IV	0.0861181	0	0	0.04084921	0
TCGA-D8-A1X7	0.0658515	0	0	0	0
TCGA-AO-A0J2	0.0237042	0	0.00588157	0.03023945	0
TCGA-A2-A04Y	0.0758111	0	0.08114937	0.07187853	0
TCGA-BH-A0B0	0.0917598	0	0.00618201	0.08401124	0
TCGA-EW-A1P1	0.0019411	0	0	0.06658578	0
TCGA-OL-A66J	0.0742472	0	0.02407583	0.04741561	0
TCGA-A8-A08A	0.1033496	0	0	0.02145192	0
TCGA-AO-A03O	0.0178578	0	0.04817039	0	0
TCGA-AR-A2LQ	0.0001812	0	0.0552109	0	0
TCGA-AN-A0AM	0.1132179	0	0.07418819	0.01714326	0
TCGA-A2-A0SY	0.1539524	0	0.05747799	0.06037867	0
TCGA-AN-A0XO	0.0072707	0	0.00207926	0	0
TCGA-BH-A0BJ	0.1041936	0	0.04715778	0.04068491	0
TCGA-BH-A0HA	0.0897104	0	0.05074191	0.03681669	0
TCGA-D8-A142	0.0472173	0	0.20370396	0.06256105	0
TCGA-E9-A3HO	0.0094431	0	0	0.09514933	0
TCGA-A2-A0T0	0.0523747	0	0	0.01399727	0
TCGA-B6-A408	0.0973046	0	0.07355411	0.01398605	0
TCGA-BH-A0EB	0.0914572	0	0.05149044	0.02785224	0
TCGA-GI-A2C9	0.0308632	0	0	0	0
TCGA-BH-A0H7	0.0849839	0	0.00579249	0.02120532	0
TCGA-AR-A24U	0.0646133	0	0.04923042	0.01225682	0
TCGA-E2-A14O	0.0292988	0	0.02216928	0.04316651	0
TCGA-E2-A10F	0.0694551	0	0	0.0412192	0
TCGA-A7-A26H	0.0670337	0	0.01045933	0.01410861	0
TCGA-BH-A42U	0.130416	0	0.01688363	0.06790427	0
TCGA-AN-A0FX	0.1398259	0	0.03641252	0.03899356	0
TCGA-EW-A1OZ	0.0566576	0	0	0.01538945	0
TCGA-A7-A0DA	0.0469431	0	0.01041516	0	0
TCGA-5L-AAT0	0.0967567	0	0.00738965	0.0981826	0
TCGA-BH-A0BQ	0.068816	0	0.04549125	0.08457208	0
TCGA-A8-A07C	0.0099005	0	0.06708752	0.0576974	0
TCGA-BH-A1FB	0.1349695	0	0.02442675	0.06649263	0
TCGA-AN-A0FL	0.0733814	0	0.00505769	0	0
TCGA-B6-A1KF	0.0007641	0.004089494	0.0243853	0.00713253	0
TCGA-A8-A09A	0.0120295	0	0	0.08984491	0
TCGA-XX-A89A	0.0533578	0	0.00878141	0.04128442	0
TCGA-A2-A4S3	0.0624904	0	0.01082796	0.08033569	0
TCGA-A8-A09M	0.0049172	0	0.08342706	0.06207335	0
TCGA-E9-A243	0.0585237	0	0.04818937	0.07003778	0
TCGA-EW-A1P4	0.0253807	0	0.01837392	0.17659669	0
TCGA-A7-A2KD	0.095136	0	0	0.00877952	0
TCGA-E2-A1AZ	0.1035016	0	0.00602687	0.03437055	0
TCGA-EW-A2FV	0.0153041	0	0	0.01821941	0
TCGA-LL-A5YP	0.0321467	0	0.08435167	0.03281076	0
TCGA-BH-A0C3	0.0939268	0	0	0.04491439	0
TCGA-BH-A0DG	0.1060811	0	0.0296825	0.00014276	0
TCGA-A7-A6VW	0.0440402	0	0.03808884	0.00667494	0
TCGA-E9-A1R4	0.0095997	0	0	0.02810206	0
TCGA-A7-A3J1	0.1182882	0	0.02293778	0.08567531	0
TCGA-GM-A2DA	0.0805803	0	0.0431139	0.16565845	0
TCGA-AQ-A04H	0.0901655	0	0.02036961	0.00501776	0
TCGA-C8-A8HR	0.1012755	0	0.00230609	0.09983798	0
TCGA-A2-A1G1	0.0585813	0	0.02673692	0.03916909	0

TCGA-A2-A0YI	0.0881116	0	0.03569206	0.03467264	0
TCGA-D8-A1JG	0.0688795	0	0.11707944	0.03101418	0
TCGA-BH-A0B4	0.0823778	0	0.00527662	0.03761682	0
TCGA-A8-A082	0.000465	0.003665137	0.00167301	0	0
TCGA-OL-A5RU	0.0935674	0	0.031343	0.04271765	0
TCGA-E9-A1RF	0.0110283	0	0	0.04778986	0
TCGA-E2-A1IK	0.0120927	0	0.05838951	0	0
TCGA-A2-A0D4	0.0508674	0	0.01005027	0	0
TCGA-A8-A06X	0.062075	0	0.01849865	0.03123427	0
TCGA-AC-A23H	0.0498388	0	0.04011958	0	0
TCGA-E9-A1R3	0.0348472	0	0	0.011827	0
TCGA-D8-A1JC	0.1049866	0	0.02813592	0	0
TCGA-E9-A1N5	0.0701546	0	0.00808598	0.08995943	0
TCGA-E9-A24A	0.047981	0	0.00530813	0	0
TCGA-HN-A2NL	0.0623774	0	0.03962744	0.11024203	0
TCGA-AN-A0FZ	0.121534	0	0.03648321	0	0
TCGA-E2-A14P	0.0628013	0	0.0597451	0.04237475	0
TCGA-AR-A256	0.012167	0	0	0	0
TCGA-BH-A18I	0.0385726	0	0	0.04079941	0
TCGA-AR-A2LH	0.0674366	0	0.01746255	0.04826949	0
TCGA-D8-A27R	0.0159921	0	0.10267638	0.11138788	0
TCGA-AO-A03M	0.141809	0	0.04168314	0	0
TCGA-A8-A07W	0.060997	0	0.00122001	0.02255446	0
TCGA-LD-A66U	0.0084636	0	0.05433209	0.16657094	0
TCGA-E9-A22G	0.055448	0	0.00842982	0.01760634	0
TCGA-A2-A04P	0.1046489	0	0.05375412	0.0463379	0
TCGA-E9-A227	0.0599485	0	0.02557043	0.08837996	0
TCGA-AC-A2FF	0.1451324	0	0.06962471	0.07184128	0
TCGA-B6-A0IN	0.1187171	0	0.02004576	0.00649306	0
TCGA-BH-A8FZ	0.0982646	0	0.00706224	0.00137072	0
TCGA-BH-A203	0.0152591	0	0.02781402	0.04425297	0
TCGA-AO-A12C	0.1007751	0	0.07740693	0	0
TCGA-B6-A1KC	0.0700558	0	0.03390345	0.01333173	0
TCGA-A2-A3XX	0.0968028	0	0.08858178	0.05417939	0
TCGA-OL-A5D7	0.0910629	0	0.00095106	0.12747716	0
TCGA-E9-A1RG	0.0707625	0	0.01765671	0.06121603	0
TCGA-A2-A0YF	0	0.003692762	0.02219024	0	0
TCGA-E9-A1NA	0.0330993	0	0	0.00886619	0
TCGA-A7-A0CG	0.1099671	0	0.0400521	0.05370648	0
TCGA-EW-A6SD	0.0560873	0	0.09113095	0.06487449	0
TCGA-C8-A12U	0.0286664	0	0.01608507	0.18828365	0
TCGA-AQ-A54O	0.038898	0	0	0.02188631	0
TCGA-A2-A3XW	0.0145601	0	0.00349711	0.0266294	0
TCGA-BH-A6R8	0.1007624	0	0.02513564	0.01492774	0
TCGA-BH-A0EE	0.0751019	0	0.06740139	0.03308001	0
TCGA-A8-A08L	0.094811	0	0	0.07141641	0
TCGA-GM-A2DL	0.0275725	0	0.03435843	0	0
TCGA-E9-A1NI	0.0498013	0	0	0	0
TCGA-LL-A50Y	0.0803443	0	0.01889487	0.08591867	0
TCGA-BH-A0BP	0.0024923	0.007294673	0.00676157	0	0
TCGA-AR-A1AS	0.0001663	0.008885538	0	0	0
TCGA-BH-A0RX	0.0913055	0	0.02237172	0.09417761	0
TCGA-AR-A1AK	0.1045761	0	0.00150364	0.05056447	0
TCGA-B6-A1KN	0.1822209	0	0.01883669	0.15721893	0.007625008
TCGA-AC-A5XS	0.0654336	0	0.036276	0.06974052	0
TCGA-A2-A0T3	0.075371	0	0.00391251	0.06887209	0
TCGA-AR-A2LO	0.1458318	0	0.05247251	0.11656098	0
TCGA-E2-A1L7	0.062384	0	0.01694492	0.17188986	0

TCGA-D8-A1XM	0.0832935	0	0.00301451	0.05401557	0
TCGA-AO-A0JE	0.09378	0	0.0501987	0.05944628	0
TCGA-AC-A6IX	0.2094099	0	0.0103982	0.07412315	0
TCGA-BH-A1EU	0.1541005	0	0.00026996	0.04475329	0
TCGA-BH-A0E6	0.1058897	0.005587499	0.1790684	0.04620467	0
TCGA-A2-A0CX	0.0701974	0	0.01488934	0.00228526	0
TCGA-3C-AALI	0.0816631	0	0.00880108	0.10749473	0
TCGA-A8-A07P	0.0682121	0	0.11057298	0.01878622	0
TCGA-A8-A09I	0.0099293	0	0	0.05560092	0
TCGA-A2-A04T	0.1107625	0	0.01141977	0.07218574	0
TCGA-E2-A152	0.0597091	0	0.07690098	0	0
TCGA-AC-A3W6	0.0726907	0	0.00535975	0.09319028	0
TCGA-BH-A0BG	0.1270271	0	0.00812946	0.123299	0
TCGA-A8-A096	0.0393379	0	0.00363401	0.0334849	0
TCGA-AR-A2LL	0.0305354	0	0.0201826	0	0
TCGA-A2-A0D0	0.0482907	0	0	0.01067629	0
TCGA-AN-A0AL	0.1494266	0	0.08864772	0	0
TCGA-AO-A0JL	0.126749	0	0.04716371	0.01418416	0
TCGA-C8-A12P	0.0615256	0	0.04815835	0	0
TCGA-E2-A153	0	0.005106021	0	0	0
TCGA-BH-A1FN	0.0814958	0	0.00555405	0	0
TCGA-AC-A3YI	0.0572637	0	0.04144351	0.00489833	0
TCGA-AR-A5QM	0.0902691	0	0.02038724	0.01294233	0
TCGA-C8-A275	0.043809	0	0.02505267	0	0
TCGA-AC-A62Y	0.0266069	0	0	0	0
TCGA-A2-A0EV	0.1181848	0	0.03914031	0.01220724	0
TCGA-C8-A1HG	0.0081876	0	0.14582523	0.11153396	0
TCGA-AO-A12D	0.048556	0	0.0691632	0.06050489	0
TCGA-GM-A2DD	0.0614402	0	0.00218475	0.08373279	0
TCGA-C8-A132	0.1508574	0	0.00851068	0.06858369	0
TCGA-B6-A402	0.0010473	0.009383695	0.00403815	0	0
TCGA-C8-A26Y	0.11098	0	0	0.04536113	0
TCGA-AN-A0XU	0.1132776	0	0.06897306	0.0767478	0
TCGA-LL-A5YO	0.1182499	0	0	0.14999133	0
TCGA-LL-A7T0	0.0457943	0	0.10653212	0	0
TCGA-AN-A046	0.0310957	0	0	0.08919571	0
TCGA-E2-A9RU	0	0.001772036	0.01206345	0.02095817	0
TCGA-E2-A150	0.0793924	0	0	0	0
TCGA-A1-A0SI	0.0500222	0	0.02664042	0.00897877	0
TCGA-Z7-A8R5	0.04581	0	0	0.08348115	0
TCGA-AR-A0TW	0.0179151	0	0.10646663	0.11016684	0
TCGA-AN-A0FN	0.1340756	0	0.01664256	0.03940707	0
TCGA-EW-A1P8	0.0323581	0	0.00447037	0.02851083	0
TCGA-E2-A14N	0.0669156	0	0	0.05381	0
TCGA-BH-A0GY	0.0410566	0	0	0	0
TCGA-BH-A0DQ	0.0558653	0	0.03429943	0	0
TCGA-AR-A1AI	0.0540731	0	0.07441676	0.04953131	0
TCGA-PL-A8LV	0.1263762	0	0.10448823	0.06997026	0
TCGA-A1-A0SP	0.0723149	0	0.02431997	0.02177854	0
TCGA-AO-A0JI	0.1076498	0	0.09481521	0.03025395	0
TCGA-A2-A0YH	0.0088577	0	0.06157323	0.03350006	0
TCGA-A7-A4SA	0.1177686	0	0.0042555	0.11798405	0
TCGA-BH-A0B2	0.1209036	0	0	0.02274055	0
TCGA-AR-A0TX	0.0935294	0	0.01701828	0	0
TCGA-AC-A2FO	0.1472969	0	0.02360931	0.06794385	0
TCGA-GM-A2DH	0.1664629	0	0	0.01551357	0
TCGA-AC-A3BB	0.1148047	0	0.05939201	0.14440218	0
TCGA-OK-A5Q2	0.2400674	0	0.00874337	0.0741996	0

TCGA-AO-A0J6	0	0	0.0024511	0.11891961	0
TCGA-E2-A3DX	0.1144809	0	0.0204416	0.16214069	0
TCGA-A8-A0A6	0.0994965	0	0.05448518	0.0236389	0
TCGA-GI-A2C8	0.0705364	0	0	0	0
TCGA-C8-A12Q	0.0498203	0	0	0	0
TCGA-C8-A1HF	0.0079707	0	0.06678926	0.07773535	0
TCGA-EW-A1J6	0.2103409	0	0.02570776	0	0
TCGA-A2-A04V	0.0174726	0	0	0.0246658	0
TCGA-AC-A2FE	0.0613716	0	0.0079496	0.1546163	0
TCGA-A8-A075	0	0	0.13426366	0	0
TCGA-AO-A03T	0.099669	0	0.08812879	0.03589384	0
TCGA-E2-A1IF	0.0977861	0	0.01675111	0.03870864	0
TCGA-D8-A141	0.0975392	0	0.0009123	0.07314879	0
TCGA-AC-A23G	0.0443818	0	0	0.03457459	0
TCGA-A2-A0T4	0.1073375	0	0	0.03155318	0
TCGA-AO-A0J8	0.084144	0	0	0.016168	0
TCGA-E9-A22A	0.0616369	0	0.03023055	0.05623679	0
TCGA-E2-A14T	0.0510205	0	0.01877566	0.02012884	0
TCGA-BH-A0HB	0.0539374	0	0.00398191	0.06090279	0
TCGA-A2-A0EN	0.1232466	0	0	0.07436968	0
TCGA-BH-A1FC	0.0481103	0	0.20694457	0.02915136	0
TCGA-AR-A252	0.0211214	0	0.08491098	0.05095737	0
TCGA-AC-A5XU	0.0455494	0	0.18037191	0.01252527	0
TCGA-E2-A15A	0.1800632	0	0	0	0
TCGA-B6-A0WS	0.0862211	0	0.02962785	0.02454307	0
TCGA-E9-A1NE	0.0911628	0	0.01014198	0.10549545	0
TCGA-A2-A0D2	0.0911066	0	0.02943069	0.06495529	0
TCGA-A2-A0CM	0.0154802	0	0	0.03719453	0
TCGA-A8-A08Z	0.0318847	0	0.00020088	0.01538228	0
TCGA-AC-A8OQ	0.0499368	0	0.18551582	0.01135856	0
TCGA-A8-A07I	0.0341197	0	0.06081551	0.01145013	0
TCGA-A2-A25D	0.1263995	0	0	0.0300271	0
TCGA-AO-A0J4	0.133907	0	0.01824719	0.00787116	0
TCGA-BH-A0AY	0.0447086	0	0.04138151	0.05731821	0
TCGA-AN-A0XW	0.0668066	0	0	0	0
TCGA-E2-A1LA	0.0422433	0	0.01905684	0.11779435	0
TCGA-AR-A1AT	0.0712509	0	0.01057782	0.08453653	0
TCGA-AC-A3OD	0.0562798	0	0.03190931	0.00782438	0
TCGA-E2-A14Z	0.0832064	0	0	0.02057193	0
TCGA-BH-A1FU	0.1001116	0	0	0.0103822	0
TCGA-AR-A0U0	0.0572918	0	0.0752297	0.03391323	0
TCGA-LL-A740	0.037058	0	0.00429499	0	0
TCGA-A8-A09X	0.1164683	0	0.04096315	0.04946289	0
TCGA-E2-A1LE	0.081141	0	0	0.09241036	0
TCGA-A2-A0YK	0.1423119	0	0.01160481	0.13325624	0
TCGA-BH-A1F6	0.0574623	0	0.03646213	0.03786225	0
TCGA-E2-A573	0.0985567	0	0.08889483	0	0
TCGA-AR-A0TP	0.0003451	0.005370548	0	0	0
TCGA-LL-A7SZ	0.0227727	0	0.31696012	0.08824312	0
TCGA-AO-A03R	0.0349745	0.003387035	0	0.10186717	0.031313185
TCGA-BH-A0DH	0.0211895	0	0.23206286	0	0
TCGA-EW-A2FW	0.1005076	0	0.14537319	0	0
TCGA-E9-A3X8	0.0115498	0	0.15900652	0.15769977	0
TCGA-XX-A899	0.1083643	0.00895671	0	0.0660237	0
TCGA-A2-A04X	0.0212268	0	0.05568138	0.01497868	0
TCGA-BH-A18K	0.0809822	0	0.00979892	0.0127597	0
TCGA-A8-A097	0.091411	0	0	0	0
TCGA-A7-A4SD	0.0449356	0	0.10277006	0.05680936	0

TCGA-B6-A0IA	0.1313438	0	0	0	0
TCGA-E2-A15M	0.0661712	0	0.01211668	0.00906298	0
TCGA-BH-A0DI	0.0781981	0	0.05816878	0.00125264	0
TCGA-BH-A0DZ	0.0301379	0	0.12259584	0.02468999	0
TCGA-A2-A3Y0	0.0063357	0.004619021	0.00588628	0.17489357	0
TCGA-BH-A1EY	0.0527304	0	0	0.02704491	0
TCGA-GM-A2DN	0.0448116	0	0.09139534	0.05840537	0
TCGA-A2-A0T1	0.0509355	0	0.05780374	0.02837819	0
TCGA-BH-A18J	0.0422539	0	0	0	0
TCGA-A8-A09R	0.0670902	0	0.00594897	0.04747947	0
TCGA-LL-A73Z	0.075445	0	0.00120673	0.05099327	0
TCGA-A8-A09D	0.078563	0	0.0084341	0.00279292	0
TCGA-E2-A1B1	0.0934911	0	0	0.03779452	0
TCGA-EW-A2FR	0.0593825	0	0.04663867	0	0
TCGA-BH-A18H	0.112218	0	0.03431049	0.11345842	0
TCGA-C8-A1HM	0.0731914	0	0.03776334	0.13102009	0
TCGA-AR-A0TQ	0.0610852	0	0.01810404	0.08500185	0
TCGA-AO-A1KQ	0.0794394	0	0.01228832	0.16523952	0
TCGA-C8-A12W	0.0687681	0	0.16342567	0.01250983	0
TCGA-BH-A0BL	0.0966864	0	0.00684183	0.05152798	0
TCGA-D8-A1XL	0.0455174	0	0.00566595	0.01373678	0
TCGA-AO-A0J9	0.0569357	0	1.50E-05	0.10133134	0
TCGA-E2-A1LH	0.0432602	0	0.05927666	0.07592028	0
TCGA-AN-A0FV	0.0239002	0	0	0	0
TCGA-D8-A1J8	0.0469336	0	0	0.04441439	0
TCGA-AR-A1AQ	0.0265996	0	0.10310596	0.02174807	0
TCGA-LL-A6FR	0.0011366	0.007395906	0.00207865	0	0
TCGA-BH-A0B7	0.071791	0	0.0085818	0.05302089	0
TCGA-AO-A129	0.0967346	0	0.03310371	0.02541273	0
TCGA-AR-A0TY	0.1177066	0	0.09399503	0.02786601	0
TCGA-BH-A1F0	0.0612605	0	0.03218184	0.02766022	0
TCGA-AN-A0AT	0.1020281	0	0.02438641	0.02285599	0
TCGA-BH-A0BR	0.0475726	0	0	0.02476565	0
TCGA-E9-A22E	0.0738724	0	0.00527914	0.05114364	0
TCGA-AR-A1AX	0.1424093	0	0	0.05418016	0
TCGA-PL-A8LZ	0.0718765	0	0	0	0
TCGA-AR-A1AJ	0.0149217	0.010636907	0.00537223	0.00569243	0
TCGA-A8-A08X	0.0565455	0	0.23703966	0	0
TCGA-BH-A0BF	0.1083349	0	0.08935417	0.04197013	0
TCGA-A2-A0SW	0.0660928	0	0.19351146	0.11139385	0
TCGA-A7-A6VY	0.0458181	0	0.08802682	0	0
TCGA-E2-A159	0.0595363	0	0.05274477	0.09681241	0
TCGA-E9-A1RB	0.0900752	0	0	0.00283833	0
TCGA-A8-A06Z	0.0432337	0	0.05059699	0	0
TCGA-BH-A18V	0.061615	0	0	0.02354974	0
TCGA-5L-AAT1	0.0647405	0	0.022474	0.13896296	0
TCGA-D8-A1JL	0.0343223	0	0	0	0
TCGA-GM-A3XL	0.0148739	0	0.08421564	0.01721726	0
TCGA-A7-A4SE	0.0411493	0	0.00043583	0	0
TCGA-BH-A0HQ	0.053657	0	0.04788805	0	0
TCGA-AR-A254	0.0773943	0	0.02935733	0.03921219	0
TCGA-D8-A1Y3	0.0428783	0	0	0.01912804	0
TCGA-E9-A248	0.0335454	0	0.15042132	0.05391526	0
TCGA-EW-A1IZ	0.1185974	0	0.0349817	0.25263232	0
TCGA-E2-A1II	0.0231498	0	0	0.06209879	0
TCGA-PE-A5DE	0.0937484	0	0.01788321	0.15211382	0
TCGA-D8-A1JJ	0.0563167	0	0.07055045	0	0
TCGA-BH-A0B3	0.0428579	0	0.15850934	0.06280569	0

TCGA-D8-A1JB	0.1658611	0	0	0.07752938	0
TCGA-EW-A1P5	0.0077005	0	0	0	0
TCGA-AQ-A04J	0.2105296	0	0.01047234	0.02700498	0
TCGA-E2-A1IH	0.025262	0.022151814	0	0.10438688	0
TCGA-D8-A73U	0.0540648	0	0.04666958	0.0969093	0
TCGA-BH-A0B9	0.0710711	0	0.00645864	0.11389521	0
TCGA-EW-A3E8	0.0926669	0	0.02226219	0.06735837	0
TCGA-AC-A3W5	0.0745301	0	0.00827511	0.03662486	0
TCGA-AR-A0TT	0.0826348	0	0.05894982	0.00700027	0
TCGA-C8-A278	0.0970355	0	0.171537	0.01974325	0
TCGA-BH-A2L8	0.0893208	0	0.01287111	0.18772229	0
TCGA-E2-A108	0.0898406	0	0	0.15101041	0
TCGA-D8-A27M	0.1286811	0	0.0599046	0.04580871	0
TCGA-BH-A0DS	0.0875018	0	0.01798521	0.07666229	0
TCGA-B6-A0IJ	0.0320877	0	0.05770916	0.04288863	0
TCGA-AC-A3HN	0.0864581	0	0.02085026	0.03306565	0
TCGA-D8-A27F	0.0533584	0	0.00967134	0.00915936	0
TCGA-AN-A0AS	0	0.005494097	0.00191451	0	0
TCGA-AN-A0FD	0.0041427	0	0.00947125	0.01003702	0
TCGA-E9-A1QZ	0.0136796	0.042642222	0	0.04943111	0
TCGA-AN-A0AJ	0.0462552	0	0.01031048	0.09455271	0
TCGA-AC-A2FB	0.1067801	0	0.01242724	0.09769588	0
TCGA-GM-A2DF	0.062261	0	0.1130823	0.04216629	0
TCGA-A8-A094	0.0856861	0	0.05359068	0.03118149	0
TCGA-AC-A62V	0.0301462	0	0	0.01930052	0
TCGA-AO-A0JM	0.0970558	0	0.08442046	0	0
TCGA-EW-A3U0	0.0764632	0	0.02890528	0.06347834	0
TCGA-C8-A12O	0.0797514	0	0.0301493	0.00997306	0
TCGA-AQ-A7U7	0.1225576	0	0.02769687	0.0562008	0
TCGA-D8-A1XT	0.0528556	0	0	0	0
TCGA-A2-A3XZ	0.2172126	0.019454623	0	0.06793981	0
TCGA-BH-A0C0	0.0467593	0	0	0.0740196	0
TCGA-BH-A0W7	0.0361005	0	0.02945006	0.04286975	0
TCGA-D8-A1XQ	0.0330319	0	0.10759984	0.02987773	0
TCGA-EW-A1IY	0.0275477	0	0.00298479	0	0
TCGA-AR-A250	0.0535132	0	0.00861184	0	0
TCGA-AC-A6IW	0.0021789	0	0	0	0
TCGA-AR-A24S	0.0485612	0	0	0	0
TCGA-3C-AALJ	0.0835601	0	0.01416754	0.02910764	0
TCGA-BH-A0DK	0.0878586	0	0.02490064	0.10011708	0
TCGA-GM-A2DK	0.0563054	0	0.00683877	0.02413111	0
TCGA-A8-A07J	0.0637683	0	0	0	0
TCGA-A8-A08F	0.0061062	0	0	0.03904952	0
TCGA-E9-A1N6	0.017117	0	0	0.0235849	0
TCGA-A2-A0YM	0.020871	0	0	0.02704788	0
TCGA-GM-A2DB	0.1179167	0	0.01330576	0.07016013	0
TCGA-GM-A2DI	0.0938846	0	0	0.15661556	0
TCGA-BH-A0BC	0.088606	0	0	0.04737435	0
TCGA-AC-A23E	0.0702164	0	0.02067901	0	0
TCGA-D8-A27N	0.0618351	0	0.04634953	0.008115	0
TCGA-A8-A08I	0.0682235	0	0.02221996	0.09146538	0
TCGA-C8-A138	0.0628436	0	0.03663689	0.04949596	0
TCGA-LD-A74U	0.0564065	0	0	0.00490019	0
TCGA-A2-A0EQ	0.0797071	0	0.04989663	0.02481851	0
TCGA-OL-A5S0	0.0454579	0	0.00936348	0.01933648	0
TCGA-AN-A0XN	0.0022796	0.001320216	0	0	0
TCGA-AC-A5EH	0.0379413	0	0	0.01513581	0
TCGA-AO-A128	0.0368213	0	0	0.11687067	0

TCGA-E9-A2JT	0.332557	0.081539334	0	0.14606638	0
TCGA-A8-A07U	0.1044075	0	0.06200985	0.04319489	0
TCGA-A2-A3XT	0.0251772	0	0	0	0
TCGA-E2-A1B6	0.0903459	0.041688605	0.0137075	0.14666441	0
TCGA-BH-A0BZ	0.0573562	0	0.04361739	0.00607	0
TCGA-AR-A1AO	0.01818	0	0	0	0
TCGA-EW-A6SB	0.0229035	0.005056688	0.23150617	0.07307841	0
TCGA-E9-A1NC	0.0469678	0	0.05570775	0	0
TCGA-E9-A228	0.0013233	0.002404962	0	0	0
TCGA-AN-A0FF	0.0339665	0	0.02438665	0	0.018697548
TCGA-E9-A22D	0.065815	0	0.01822498	0.16510492	0
TCGA-A2-A0SX	0.0288556	0	0.05011893	0.01215966	0
TCGA-EW-A1P7	0.1782949	0.087556933	0.01482782	0.11681596	0
TCGA-D8-A1Y2	0.0745892	0	0.00217178	0.00796141	0
TCGA-E2-A10A	0.0340456	0	0.03999983	0	0
TCGA-BH-A0E1	0.0698388	0	0.045855	0.01300639	0
TCGA-A8-A08R	0.0188619	0	0.03961606	0	0
TCGA-EW-A1OV	0.1305648	0	0.02060668	0.13993275	0
TCGA-A8-A08P	0.02812	0	0	0.02104742	0
TCGA-LL-A73Y	0.0683038	0	0.00433986	0	0
TCGA-BH-A0B6	0.0443058	0	0.02889826	0.15852094	0
TCGA-AR-A24Q	0.0526789	0	0.08463925	0.02112135	0
TCGA-A7-A56D	0.0006242	0	0.07652046	0.00927352	0
TCGA-AR-A0TS	0.2332897	0	0.01532132	0.02405686	0
TCGA-B6-A401	0.0458061	0.006627656	0	0	0
TCGA-E2-A1B5	0.1113824	0	0.02033794	0.11590787	0
TCGA-B6-A400	0.0065811	0	0	0	0
TCGA-A2-A3XY	0.0523504	0	0	0	0
TCGA-C8-A27B	0.0410135	0	0.02606231	0.03919694	0
TCGA-C8-A12V	0.082808	0	0.02452979	0.08323416	0
TCGA-BH-A0BW	0.0692707	0	0.01524692	0.0109465	0
TCGA-A2-A25F	0.052641	0	0	0.03922843	0
TCGA-C8-A26X	0.0575535	0	0.11902017	0	0
TCGA-BH-A0W5	0.254664	0	0.00702083	0.04808569	0
TCGA-E9-A1NG	0.0315256	0	0.00237572	0	0
TCGA-S3-AA17	0.0890448	0	0.06186706	0.01708257	0
TCGA-A2-A0CR	0.083495	0	0.00342095	0.18774804	0
TCGA-E2-A14R	0.0519673	0	0.07807142	0.05268509	0
TCGA-AO-A0JC	0.2848126	0.016225167	0	0.12835374	0
TCGA-A7-A6VV	0.0467538	0	0.01097064	0	0
TCGA-B6-A3ZX	0.339772	0	0.00893385	0.10957416	0
TCGA-BH-A0AV	0.0543369	0	0.02254811	0	0
TCGA-AR-A0TU	0.0130152	0	0	0.06712245	0
TCGA-A8-A090	0.0379924	0	0.00757538	0.00841313	0
TCGA-C8-A134	0.0759181	0	0.00727947	0.0415222	0
TCGA-BH-A0DX	0.0210228	0	0.0002952	0	0
TCGA-LL-A441	0.2686383	0.027412373	0	0.01433316	0
TCGA-B6-A0I2	0.0589686	0	0.0341413	0.1552258	0
TCGA-WT-AB41	0.0760169	0	0.04309597	0.15786062	0
TCGA-JL-A3YW	0.0434877	0	0.00341596	0	0
TCGA-A8-A07O	0.0479217	0	0.00117017	0.02748855	0
TCGA-A8-A08J	0.0651778	0	0.11825257	0.01537213	0
TCGA-OL-A5RY	0.1721076	0	0.02554924	0.07039505	0
TCGA-E9-A3QA	0.0741779	0	0	0.05409731	0
TCGA-AR-A1AR	0.0131368	0	0	0	0
TCGA-A2-A04Q	0.0804139	0	0.04851762	0.10660553	0
TCGA-E9-A1N4	0.0527895	0	0.00072745	0.00614951	0
TCGA-GM-A2DO	0.2231369	0	0.01716904	0.12695411	0

TCGA-B6-A0RT	0.0729783	0	0.00091336	0.10773926	0
TCGA-E9-A1NF	0.0482641	0	0.00145204	0	0
TCGA-C8-A12K	0.0298702	0	0.01113039	0.07601056	0
TCGA-BH-A209	0.0830187	0	0.02565495	0.08938891	0
TCGA-BH-A8G0	0.139494	0.017693032	0	0.00304391	0
TCGA-A2-A0CZ	0.0410292	0	0.05016039	0.0327846	0
TCGA-A2-A0ST	0.0679182	0.001831622	0.04366982	0.09827819	0
TCGA-LL-A6FQ	0.0431769	0	0.004828	0.01990408	0
TCGA-LL-A8F5	0.0471416	0	0.23964585	0	0
TCGA-A2-A0CL	0.1153181	0	0.00693814	0.08572203	0
TCGA-AQ-A0Y5	0.069076	0	0.10834784	0	0
TCGA-E9-A5FK	0.1218379	0	0.01932069	0.19888241	0
TCGA-A8-A0A1	0.0455984	0	0	0.01670894	0
TCGA-OL-A66P	0.0256584	0	0.18308254	0.20171319	0
TCGA-LL-A5YN	0.1498061	0	0	0.11057385	0
TCGA-OL-A66I	0.0028455	0	0.01025893	0.20284548	0
TCGA-LL-A5YM	0.0006643	0	0.11350666	0.00621809	0
TCGA-LD-A9QF	0.0480573	0	0	0.07032341	0
TCGA-BH-A0AW	0.0759989	0	0.02184995	0.08108276	0
TCGA-A8-A06R	0	0	0.13166197	0	0
TCGA-AR-A0U4	0	0	0.00183501	0.01123401	0
TCGA-E2-A1L9	0.0014265	0.005979901	0	0	0
TCGA-BH-A5J0	0.0547375	0	0.01569824	0.04702143	0
TCGA-E9-A1ND	0.0091778	0	0	0.02555058	0
TCGA-AO-A03N	0.1124278	0	0.04055922	0	0
TCGA-AR-A1AN	0.025369	0	0.00236944	0.05270769	0
TCGA-E2-A1L6	0.0234843	0	0.00212813	0	0
TCGA-LL-A440	0.0350988	0	0	0.00623813	0
TCGA-S3-AA15	0.1446641	0	0.24325992	0.05253274	0
TCGA-AR-A1AW	0.0860818	0	0.08800047	0.03314035	0
TCGA-D8-A13Z	0.0828189	0	0	0	0
TCGA-BH-A0HF	0.0687389	0	0.01212788	0	0
TCGA-BH-A0H5	0.0657402	0	0.02415786	0.08389558	0
TCGA-A8-A08H	0.0269271	0	0	0.01772302	0
TCGA-A8-A0A7	0.0709679	0	0.06951039	0.09154533	0
TCGA-A2-A0EP	0.092007	0	0	0.03216269	0
TCGA-AR-A2LJ	0.0215761	0	0.00073548	0.02863394	0
TCGA-A7-A13H	0.056479	0	0.01370093	0.01539741	0
TCGA-EW-A1PB	0.0552629	0	0	0.0760916	0
TCGA-S3-AA10	0.0708483	0	0.03701382	0.09768513	0

T cells CD4 memory resting	T cells CD4 memory activated	T cells follicular helper
0.297944733	0.019112609	0
0.223788619	0	0.030086359
0.371995809	0	0
0.186434995	0	0.030356892
0.227499495	0	0.118785901
0.323420968	0	0.009296051
0.232657731	0.012081963	0.016932654
0.339908971	0	0.053798354
0.102828821	0	0.029211344
0.176179534	0	0.070606966
0.353373061	0.004749285	0.020674642
0.252636752	0	0
0.248626566	0	0.047334554
0.345045195	0.002536583	0
0.20017986	0	0.010365036
0.26425548	0	0
0.335217657	0	0.038825497
0.127962772	0	0.023290463
0.214967792	0	0.0412968
0.273556942	0	0.04685336
0.225735956	0	0.031197068
0.251051519	0.004758978	0.014289513
0.191383469	0	0.075053631
0.199360999	0	0.018341195
0.402114558	0.005181243	0
0.146083053	0	0.026161303
0.31799458	0	0.008879814
0.312891301	0	0.004566775
0.31115454	0	0.005366575
0.17634867	0	0.015785369
0.229110661	0.003066393	0.013265357
0.188307441	0	0.020014805
0.300288575	0	0.004335119
0.270991035	0.016816905	0
0.326202228	0.008181628	0.039669702
0.37756071	0.018494939	0
0.200613076	0	0.008801499
0.1985739	0	0.043656687
0.234891581	0	0
0.395188497	0.00387837	0
0.25086333	0.006114541	0.047752945
0.286882171	0	0
0.345896194	0.0242574	0
0.218380545	0.058555106	0.009904823
0.257932223	0	0.044298792
0.415734119	0.032736963	0.030093425
0.455606555	0	0.050304388
0.25964741	0	0.042198917
0.399291608	0	0
0.339453457	0	0.020036177
0.099489241	0	0.054798099
0.310339626	0.015921938	0.010829469
0.418318241	0.009328635	0
0.366460967	0	0.005364358
0.206711079	0	0.022741527
0.2434559	0	0.022874809
0.270886011	0.043195227	0.024375939

0.288799662	0.009084401	0.005918973
0.303000368	0.01978362	0.000315782
0.30834972	0	0.010447874
0.31572948	0.000518992	0.012922411
0.335543427	0	0
0.318333366	0	0.052052394
0.329918832	0	0.006896513
0.39065315	0	0.053040151
0.153972323	0.001140756	0.078685557
0.080957145	0	0.039472677
0.278059295	0	0.008489079
0.222309088	0	0.042295832
0.261251072	0	0.014518184
0.225290294	0	0.00617164
0.220274134	0	0.048332693
0.066251836	0	0.02842003
0.313977901	0	0.040960326
0.299591565	0	0.058130742
0.361091438	0	0.005020731
0.482859991	0	0
0.219245737	0	0
0.262160463	0.00604789	0.066623004
0.185085878	0	0.022906131
0.271941918	0	0.037306776
0.17845843	0.01146998	0.083156729
0.326004723	0	0.038751962
0.391053493	0.011933076	0
0.349635242	0.006867769	0
0.301479387	0.007852587	0.009431255
0.235269508	0.019484101	0.026209895
0.203101947	0	0.032564375
0.055941512	0	0.017453394
0.095275334	0	0.011173972
0.149608461	0.013083045	0.028431275
0.253718011	0.022200462	0.061850031
0.127554464	0	0.022786769
0.197183814	0	0.033673387
0.212352623	0	0.005052895
0.473548948	0.010403961	0
0.224736519	0.019553774	0.040945688
0.194143136	0.059156273	0.044998448
0.075661017	0	0.119724239
0.210656467	0	0.008086895
0.18437981	0	0.05663543
0.232321395	0	0.040855194
0.344384099	0	0.030215336
0.186737974	0	0.018257312
0.137685525	0	0.029913708
0.16105387	0	0.016463489
0.175976655	0	0.03911066
0.325286224	0	0.008291245
0.224891965	0.062791781	0.026778601
0.317238953	0.004186833	0.053045194
0.193863253	0	0
0.342553923	0	0.000481323
0.193175313	0	0.068940084
0.365195133	0.003612242	0.024903065
0.3208427	0.010483835	0.019148283

0.336127542	0.012890631	0
0.265390163	0.020734797	0.04188408
0.389710261	0	0
0.404031323	0.000703392	0
0.068034796	0	0.033301346
0.307563756	0	0.037372311
0.308153148	0	0.04021079
0.191093778	0.00615088	0
0.267332202	0	0.104093249
0.301384948	0	0.087940582
0.190098316	0	0.001878903
0.361699581	0	0.008224861
0.225205367	0.023771452	0.058520402
0.369560744	0.001965881	0.022073163
0.12205917	0	0.025514198
0.314585271	0.012005462	0.081899069
0.295634468	0	0.005685994
0.103856582	0	0.053643435
0.282201847	0	0.021279347
0.230262531	0	0.014258388
0.117417497	0	0.070027201
0.09433704	0	0.007835824
0.174306712	0	0.009030516
0.318855647	0.01446942	0.049044695
0.170725394	0	0.021507472
0.232136228	0	0.021731997
0.286441466	0.035041768	0.02741144
0.359182708	0	0.018670813
0.408917015	0.032456596	0
0.331299662	0	0
0.159923571	0	0.060045924
0.119767991	0	0.063379787
0.099475095	9.86E-05	0.082115232
0.166034007	0	0.025159604
0.171913694	0	0.03014024
0.312515385	0.030184992	0.078647122
0.168800013	0	0.039114852
0.236001175	0	0.061212537
0.232275331	0	0.083346075
0.158786022	0	0.018630506
0.25620312	0.010523907	0.058480531
0.395206639	0.013358383	0.040124978
0.194325145	0	0.033988974
0.43344312	0.032820675	0.018721629
0.217357319	0	0.016844448
0.094224687	0	0.016956581
0.33725886	0.022534036	0.034170737
0.20219388	0	0.003038203
0.236326333	0	0.006248276
0.18690316	0	0
0.244647371	0.001826231	0.002531567
0.422541982	0.007672126	0
0.301604339	0	0.017630156
0.300305181	0.020941488	0.009227847
0.42415181	0.010787856	0
0.357169973	0	0.058357042
0.232671899	0	0.052562726
0.404834113	0.004528694	0

0.13759177	0	0.118463787
0.439658332	0.008415076	0
0.422268853	0	0.013797502
0.125537229	0	0.011821959
0.17613832	0	0.054581496
0.369307435	0.042684531	0.039135991
0.26551174	0.002445936	0.075671377
0.191536819	0	0.007954562
0.426193478	0.004994275	0.001600194
0.224228703	0	0
0.231442755	0	0.012429158
0.158382719	0	0.000335692
0.39072717	0	0.013600798
0.096821627	0	0.003428722
0.160136486	0	0.024204625
0.180069938	0	0.029845638
0.31254843	0.003766155	0.016383058
0.066865901	0	0.007686151
0.343590116	0.010713012	0.001495395
0.393807419	0	0.008838787
0.247167294	0.081404854	0.068572878
0.500010497	0.003364985	0
0.144568013	0	0.016931332
0.288247135	0	0.0057378
0.320256395	0	0.016784954
0.527800055	0.026744165	0
0.270881661	0.031156635	0
0.375405636	0.00372861	0.055682334
0.227755902	0	0
0.233840272	0.001909849	0.022405815
0.181933924	0	0.014758808
0.402475056	0.001260867	0.048506877
0.373800533	0	0.031339651
0.240746324	0	0.007544733
0.364215345	0.003195717	0.070107972
0.192636168	0.037078	0.043259753
0.348157542	0.003203955	0.00675478
0.568645102	0.011033417	0
0.333017148	0.018679505	0.04020215
0.376363904	0.010406804	0
0.233607548	0.023209381	0.048342214
0.151677157	0	0.024621985
0.310856446	0.011424642	9.26E-05
0.30653316	0	0.020252209
0.406409693	0.022728145	0.004022692
0.210654557	0.006419365	0.014349096
0.202363469	0	0.065953862
0.153126083	0	0.007185516
0.190622651	0.009119358	0.018267882
0.31232176	0	0
0.143385467	0	0
0.129927403	0	0.012912134
0.170264182	0	0.023746986
0.355955222	0.017694273	0
0.35824499	0	0.03253993
0.108003302	0	0.067617405
0.175679364	0	0.019660593
0.245513934	0.007339885	0.04763148

0.049000295	0	0.09851809
0.535052357	0.019743609	0
0.170811999	0	0.008340603
0.156026551	0.007336877	0.014008098
0.210912335	0	0.076845116
0.386073362	0.00365048	0.000283409
0.342640666	0.017637334	0
0.262139383	0.006477109	0.010871373
0.098486452	0	0.002353928
0.11788713	0.003760532	0.061796123
0.228840743	0	0.02353558
0.196791773	0	0.01056173
0.308876069	0.000220801	0.003938757
0.169375528	0	0.025746738
0.334701239	0.020431139	0
0.219161493	0.065723893	0.049721765
0.318658228	0.023103685	0
0.200391636	0.006243456	0.027531574
0.12666483	9.96E-05	0
0.290768486	0.040273885	0.037000511
0.378408598	0.000130226	0.047483631
0.220777875	0	0.003854613
0.253267977	0	0.068834371
0.042994718	0	0.0590058
0.300237458	0	0.108768164
0.252733573	0.010978132	0.069027699
0.116994613	0	0.02272966
0.392237975	0.020434453	0
0.324393208	0	0.041392575
0.130962897	0	0
0.315756226	0.02965282	0
0.238011475	0.002837141	0.021777337
0.188526562	0.001243692	0.024840846
0.182629938	0	0.011353067
0.279881442	0.007351169	0
0.315623676	0	0.080208156
0.346644062	0.02718593	0.03877617
0.205897504	0	0.007120539
0.264833425	0.013315041	0.038705771
0.236842263	0.00239562	0.008754139
0.151636064	0.00968126	0.064901883
0.310926031	0.034472924	0.002028424
0.113707919	0	0.063453235
0.152106512	0	0
0.283307751	0	0.059210646
0.223052889	0	0.029399003
0.329844112	0.003383321	0.076877981
0.264341901	0.020720985	0.06661648
0.224948162	0	0.048185001
0.085921354	0	0.001585539
0.306351972	0	0
0.155369433	0	0.059039011
0.300648869	0.024708482	0
0.219371725	0.001626404	0.056852294
0.386192228	0.028534423	0.07203907
0.369878971	0.006396652	0.00919181
0.105230577	0	0.019309482
0.1317405	0	0.04336346

0.254362797	0.005875333	0.056940907
0.2056651	0	0.057312011
0.428703411	0.009578188	0
0.308982083	0.002173257	0.053295789
0.308587108	0	0.019235252
0.233131057	0.016185505	0.067711454
0.335987934	0	0.003463053
0.204043244	0	0.027005356
0.264511706	0.00832585	0.019365416
0.20761635	0.018217226	0.020949852
0.26357636	0.027777556	0.040987381
0.259105433	0.009933384	0.048154994
0.224083954	0	0.054951275
0.172837988	0	0.014805241
0.188525428	0	0.028674201
0.153692517	0	0.033961702
0.200412307	0	0
0.159388546	0	0.020876849
0.170110275	0	0.038890017
0.5158513	0.000247306	0
0.228249594	0.036029406	0.047016192
0.406218776	0.052255971	0
0.179964938	0	0.034309302
0.19046456	0.018570612	0.01896144
0.069115661	0	0.000496191
0.097655233	0	0.045972894
0.286701973	0.007917392	0.015249318
0.146462601	0	0.047168932
0.417000572	0	0.038715601
0.123936043	0	0.04402074
0.261203433	0.006146872	0.029542778
0.208250165	0.005707879	0.065470209
0.272917677	0.001719391	0
0.218028113	0.011342723	0.018122364
0.515863537	0	0.04376497
0.260030147	0.00027217	0.031183361
0.276112426	0	0.059894118
0.259722295	0.008956474	0
0.291843253	0	0
0.345453684	0.013503262	0
0.187360099	0	0.017795577
0.137856097	0	0.009351341
0.23586293	0	0.001443335
0.163962573	0	0.046668117
0.184151521	0	0
0.237871887	0	0.00773632
0.34358387	0.045114834	0
0.166192984	0	0.016624862
0.145715544	0	0.02397154
0.176312476	0	0.003958631
0.179138258	0.002937371	0.010125777
0.129915875	0	0.039851826
0.099177946	0	0.005535305
0.3051072	0.006577306	0.034522863
0.143468158	0	0.026929477
0.13658874	0	0
0.172316647	0	0.043953916
0.17174808	0.051178574	0.054202973

0.250171907	0.011294674	0.013450336
0.256659509	0.023748842	0.046234018
0.143154112	0	0.017981152
0.29348415	0.041504394	0.014254499
0.355477029	0	0.011445237
0.105513475	0	0.014872694
0.200290735	0.009253788	0.086252262
0.288456824	0	0.027496561
0.191820538	0	0.006299835
0.115101132	0	0.047173885
0.228722609	0.08624477	0.022614006
0.351495429	0	0.01463696
0.266854778	0.013772988	0
0.157155462	0	0.00787648
0.159693614	0	0.018782543
0.13381617	0	0
0.223782475	0.003168604	0.003752747
0.321251517	0.016567205	0.015881297
0.327909586	0	0
0.274781521	0	0.014631791
0.220145963	0	0.013508253
0.224508262	0.002008274	0.012361506
0.03542456	0	0.016819551
0.382155901	0.003999397	0
0.114466182	0	0.144544839
0.436820463	0.012717336	0
0.141598514	0	0.034210839
0.273227889	0	0.033707775
0.212580664	0.026516782	0.033590239
0.234449054	0.012872687	0.056619452
0.253289245	0.019898793	0.038828473
0.435585287	0.033415725	0
0.188560092	0.007609314	0.033611224
0.372865072	0.015654189	0
0.204861735	0	0.004124472
0.316184034	0.00997464	0.002141362
0.284200232	0	0.017642044
0.123401146	0.001818231	0.096560184
0.361923227	0.023811105	0
0.11842266	0	0.027174271
0.215095028	0.004725657	0.054737937
0.119277266	0.00116626	0.003331965
0.205442155	0.042028935	0.069996208
0.117159879	0	0.022743676
0.142182807	0.012776731	0.03463973
0.107044731	0	0.008730457
0.203263354	0.009547454	0
0.123720223	0.00059848	0.084940476
0.172632116	0	0.055813886
0.15837084	0	0.03620466
0.160464768	0	0
0.100833917	0	0.01173248
0.356032688	0.016326641	0
0.225357196	0.027697917	0.000498564
0.367624915	0.002076699	0.004123943
0.243391143	0.030221236	0.027042562
0.254124768	0.015491248	0
0.330515891	0.017435003	0

0.389840317	0.016901518	0
0.148916187	0	0.010448989
0.172364866	0	0.030401317
0.417869756	0.041862049	0
0.159029665	0	0
0.177158542	0	0.011304846
0.268581285	0.005489192	0.045165231
0.086105737	0	0.013094335
0.170004514	0.004633501	0.045138359
0.448235759	0.020587402	0
0.139034032	0	2.82E-05
0.203053793	0	0.023142543
0.148141396	0	0.052285359
0.140794095	0	0.028264963
0.253238529	0	0.055610586
0.314072874	0.028360347	0
0.016855736	0	0.005663536
0.160261023	0	0.039546688
0.367721751	0.006939521	0
0.212750653	0	0
0.126527082	0.006418853	0.006669898
0.150767825	0	0.023411469
0.198298573	0	0.020389897
0.280742462	0.003952005	0.103401282
0.044278255	0	0.097094712
0.160380242	0	0.001366128
0.261329541	0	0.049606869
0.117232788	0	0
0.212160486	0.041622899	0
0.306246766	0.029262581	0
0.182354339	0	0.033257052
0.133301117	0	0.024019011
0.48094828	0.014969064	0
0.156450197	0	0
0.310709096	0.01277584	0
0.249365957	0.011947587	0.017141856
0.147210616	0	0.00274726
0.127900901	0	0.006141595
0.150401242	0	0.060010882
0.216805691	0.013472862	0.107213613

T cells regulatory (Tregs	T cells gamma delta	NK cells resting	NK cells activated	Monocytes
0	0	0.05102397	0	0.055028805
0.03249374	0	0	0.029527213	0.052850893
0	0	0.049296379	0	0.034398607
0	0	0.029019117	0	0
0.028064992	0	0.052381661	0.062110606	0.047798487
0.006398515	0	0.02956345	0	0.011883108
0.036976933	0	0.029660903	0	0.022423527
0	0	0.033953494	0	0.055285216
0.009639832	0	0.028973247	0.003983467	0
0.031371023	0	0.030747137	0	0.007100376
0.040987335	0	0.022396872	0.00343907	0.024373653
0.0009058	0	0.062117616	0	0.019572522
0.058090948	0	0.031005989	0.017421505	0.015028489
0.000750211	0	0.039445648	0	0.034230831
0.062445182	0	0.040418213	0.018513356	0
0.030360897	0	0.032100477	0	0.004400828
0.038401407	0	0.017091967	0.01319496	0.016004649
0.029007325	0	0	0.026088625	0.018668075
0.073864845	0	0.007315264	0.05363936	0.076183843
0.060305903	0	0.047810337	0	0
0.075082816	0	0.031936146	0	0.007658824
0.027571131	0	0.037302249	0	0.07297701
0.025326149	0	0.001540812	0.009507498	0.00068772
0.026994018	0	0.020443817	0	0
0	0	0.058102672	0	0.026521429
0.02445828	0	0.002658123	0.019375253	0
0.013103533	0	0.024860282	0	0.014946674
0	0	0.035789344	0	0.00887387
0.038227052	0	0.014536332	0.023565802	0.018310267
0.039254301	0	0.032303449	0	0
0.036891988	0	0.042716668	0	0.002197287
0.060161922	0	0.061151119	0.013795939	0.026072143
0.056693395	0	0.046540768	0	0.013310994
0	0	0.038193105	0	0.008481182
0	0	0.002841726	0.018288688	0.03456331
0	0	0.054883267	0	0.027017025
0.01208664	0	0.042035302	0	0.009278284
0.046843215	0	0.111386228	0	0
0.000818267	0	0	0	0.038463976
0	0	0.040246509	0	0.021211923
0.082814721	0	0.037380484	0	0.003605226
0.077084648	0	0	0.013251205	0.033798481
0.003326546	0	0.010726585	0.000807709	0.007280781
0	0	0.081135747	0.033214173	0.024447263
0.075188299	0	0.044263083	0	0.022469513
0	0	0.053516552	0	0.027517792
0	0	0.017581114	0	0.046947246
0	0	0.042659164	0	0.041843421
0	0	0.05106083	0	0.039786037
0	0	0.078175814	0	0.019391322
0.043983283	0	0	0.047693258	0.015447404
0.069695828	0	0.065414954	0	0.028673361
0	0	0.072975486	0	0.019398539
0.002176536	0	0	0.02200058	0.040432136
0.022302589	0	0.050628207	0	0
0.026105562	0	0.036193383	0	0.013354942
0.011785816	0	0.045312222	0	0.01977088

0.00859124	0	0.040928414	0	0.000706503
0	0	0.054385762	0	0.007290558
0.039025121	0	0.03979886	6.96E-05	0.020617411
0.04609127	0	0.203739796	0	0.050663476
0.10241367	0	0.104464934	0	0.055368796
0	0	0.035512845	0.003515248	0.034239363
0.056949091	0	0.055085331	0	0.038066101
0	0	0.079698344	0.00489259	0.054892072
0.031478193	0	0.023644531	0.005013872	0
0.034286538	0	0	0.023862111	0
0	0	0.035795796	0	0.022719522
0.044266473	0	0.029775064	0	0
0.049245788	0	0.0120006	0.002471581	0.011542119
0	0	0.039434891	0	0.03753512
0.053809989	0	0	0.019144297	0
0.058101439	0	0.006420189	0.006651708	0
0.010412548	0	0.030336812	0	0.037448389
0	0	0.020733717	0	0.001105782
0.006014515	0	0.019106822	0.016309393	0.032653897
0.021846854	0	0.061943324	0	0.092135895
0.005813463	0	0	0.001707248	0.068485715
0.019033453	0	0.045925246	0.003976418	0.023203188
0.017926366	0	0.021014294	0.005383959	0.011771169
0.104183866	0	0	0.034544311	0.045056331
0.025728663	0	0.045857628	0	0.018071396
0.033482812	0	0.038398391	0	0.016375395
0	0	0.018905327	0.00173996	0.044129679
0	0	0.026902046	0	0.015690359
0.053940797	0	0.063609466	0	0.030097553
0.010456476	0	0.035918801	0	0.041614962
0.001288658	0	0.030368452	0.016800843	0.043550892
0.018229098	0	0.004008376	0	0
0.016552483	0	0	0.010714902	0
0.003556474	0	0.069593633	0	0
0.076849909	0	0.073168027	0	0.024985343
0.084733542	0	0.000208693	0.024708568	0.029091642
0.053436816	0	0.090067441	0	0
0.004119089	0	0.024908872	0	0.118608849
0	0	0.035921251	0	0.02048698
0.030006475	0	0.048781617	0	0.014307517
0.0281945	0	0.068147223	0	0.038718536
0.056088556	0	0.019402298	0	0
0.104376202	0	0.032767088	0.017543029	0.033570356
0.081518782	0	0.031975407	0.009155238	0.024575607
0.049383284	0	0.067509678	0.009304099	0.013939267
0.024644894	0	0.057799499	0.00128081	0.040719937
0.031791945	0	0.01499165	0.007276913	0.001979277
0.017278199	0	0	0.019198203	0
0.025309228	0	0.017366229	0.004154188	0.006825359
0.026264839	0	0.06536625	0	0
0.055513898	0	0.098041359	0	0
0.013180599	0	0.055213855	0.022662862	0.023942179
0.003027093	0	0.018915606	0	0.0926971
0	0	0.046350464	0	0.031825088
0	0	0.020492701	0	0.01011984
0	0	0.043257824	0	0.05156975
0	0	0.040729397	0	0.027477096
0.015542795	0	0.019039595	0	0.012197423

0	0	0.03265007	0	0.025542604
0.066556877	0	0.02496047	0	0.029986444
0	0	0.045521192	0	0.024291605
0	0	0.016841463	0	0.012603963
0.069689526	0	0.003802193	0	0
0.007797834	0	0.025792775	0	0
0.072378356	0	0.013952521	0	0.022486094
0	0	0.010182811	0.003029818	0.050698524
0.000404726	0	0.004515546	0.018029022	0.024433037
0.001760012	0	0.014123691	0.017136611	0.035647395
0.032029016	0	0.009519964	0.02665924	0
0.052437753	0	0.059403871	0	0.017612888
0.052258635	0	0.031079658	0	0.015985306
0.008011252	0	0.065303123	0	0.100709618
0.034984415	0	0	0.018878274	0.013689123
0.026995141	0	0	0.020361114	0.009496243
0.001958355	0	0.06861046	0	0.007418382
0.033080753	0	0	0.006789064	0
0.017654801	0	0.015055104	0	0
0.044258739	0	0.052302798	0.005703572	0.027623785
0.031594725	0	0.027041386	0	0
0.033066869	0	0.020058595	0	0.021531601
0.025190722	0	0.023437454	0	0
0.001096895	0	0.029928677	0.010638013	0.020296629
0.018891059	0	0.034874147	0	0
0.023731109	0	0.016001174	0	0
0.013455929	0	0.010673945	0	0.015307425
0.000864161	0	0.058859518	0	0.026056674
0	0	0.04557462	0	0.016930818
0.039919684	0	0.072387448	0	0.040610066
0.077463157	0	0.123340136	0	0
0.051335986	0	0.043145837	0	0
0.014172719	0	0.05969044	0.018397998	0
0.112692698	0	0.028082202	0	0.015911796
0.064328782	0	0.002717484	0.036514539	0.012655822
0.005534939	0	0.093820444	0	0.043743213
0	0	0.011700506	0.011108478	0.009829541
0.061274606	0	0.060706989	0	0.006082534
0.020658885	0	0.004388236	0.035383159	0.026187428
0.008463932	0	0.062044707	0	0.063450152
0.001554411	0	0.0325232	0	0.037991163
0.001962494	0.019982421	0.014985004	0	0
0.034277053	0	0.018016327	0	0.095821539
0	0	0.044400528	0	0.016671349
0	0	0.043589274	0	0
0.031248252	0	0.02495808	0	0
0	0	0.040470097	0.009237523	0.043720678
0.054241955	0	0.043412666	0.006227019	0.019223225
0.020524879	0	0	0.033238874	0.045287765
0	0	0	0.024580787	0
0.012978493	0	0.040973835	0	0.031135138
0.00238425	0	0.056382491	0	0.024501745
0.005784233	0	0.047022917	0	0
0.052390424	0	0.075873678	0	0.020568345
0	0	0.027374154	0	0.030700102
0.030039334	0	0.058874138	0.010480519	0.034971189
0.047007208	0	0.032759148	0	0.018620861
0.003886794	0	0.03696405	0	0.013120238

0.018443449	0	0.084655642	0.043306658	0.018349906
0	0	0	0	0.018679597
0.015683414	0	0.022118529	0	0.00814678
0.017047924	0	0.035084202	0	0
0.020868234	0	0.024025286	0	0
0.033231494	0	0.007315071	0.010284153	0.019321783
0.005357434	0	0.016280835	0.019539633	0.021741891
0.065420627	0	0	0.075822085	0.037273599
0.017898725	0	0.051492388	0	0.039565109
0.000908415	0	0.010301576	0.031057083	0
0.03554194	0	0.013385203	0.030455769	0.025169404
0.050986911	0	0.009820438	0.005654688	0
0.030331947	0	0.043436998	0	0.018061805
0.030921668	0	0.006954978	0	0
0.027251925	0	0	0.01560476	0
0.049174667	0	0.015534537	0.008033126	0.003663385
0.025876226	0	0.037180738	0	0.001853577
0.008991943	0	0.005204784	0	0
0.053221113	0	0.034860249	0	0.030575212
0.03647911	0	0.067033699	0	0.02670165
0.004389217	0	0.034138783	0.012120166	0.015218821
0	0	0.045969686	0.003525043	0.053978178
0.050975572	0	0	0.033798031	0
0.037469885	0	0.175894804	0	0.046063097
0.050113817	0	0.03846915	0	0.017259662
0	0	0.02396902	0	0.024536834
0	0	0.082418774	0	0.034047773
0.055907426	0	0.041648272	0.009087802	0.035185165
0	0	0.031958959	0	0.019737378
0.08970147	0	0.063851933	0	0.035657255
0.057363651	0	0.020434666	0	0
0.006108735	0	0.083731403	0	0.040403151
0.01897342	0	0.064805855	0	0.023195418
0.021358979	0	0.000675433	0.015864735	0.015990068
0.049627834	0	0.06073916	0	0.019233526
0.046910932	0	0.018306004	0.014557972	0.009095606
0.004400319	0	0.050827717	0	0.01287571
0	0	0.033105698	0	0.021322561
0.025018066	0	0.076293561	0	0.060079886
0	0	0.066754674	0	0.019245848
0.000176486	0	0.097604018	0	0.013224807
0.041299361	0	0.019322403	0.001351654	0.012731327
0.062262038	0	0.030848682	0.00089555	0.01641933
0.057478701	0	0.033118854	0	0
0	0	0.031058412	0	0.01531054
0.019521356	0	0.058298686	0	0.020636663
0.006161997	0	0.03476508	0.027922718	0
0.0402211	0	0.095609859	0	0
0.052288279	0	0.046506579	0	0.012861039
0.00827443	0	0.021366946	0.015753563	0.07739999
0.086153459	0	0.015514137	0.021854202	0.000684173
0.018197068	0	0.034045393	0	0
0.069792214	0	0.02076059	0.017033227	0.024166449
0	0	0.041934379	0	0.035255663
0.015541159	0	0.041154893	0.010604398	0.029940522
0.021599502	0	0.021121459	0	0
0.029140696	0	0.012360514	0.000884737	0
0.005069063	0	0.071280052	0	0.006038066

0.021502626	0.039928538	0.007371397	0	0
0	0	0.032935897	0	0.028577662
0.042469447	0	0.016269072	0	0
0.025739992	0	0.019972041	0	0
0.104246519	0	0.028125759	0.037597205	0.018500005
0	0	0.039174343	0	0.020790629
0.001449297	0	0.032600819	0	0.008632151
0.05542847	0	0.027976089	0.01518866	0.030590826
0.03559392	0	0.007646207	0	0
0.042526467	0	0.03047684	0	0
0.054588012	0	0.019508881	0.015865502	0.026591804
0.027293714	0	0.034715088	0	0
0.003735999	0	0.04221495	0	0.032865528
0.028606064	0	0.005554914	0.010255945	0
0.017283332	0	0.065760719	0	0.006011103
0.050975363	0	0.056998011	0.021895434	0.012704074
0	0	0.04226126	0	0.010678614
0.046540695	0	0.035068948	0	0
0.041289402	0	0.024189559	0	0
0.004618735	0	0.060098192	0	0.02490048
0.047813836	0	0.043022923	0	0.048933157
0.038675329	0	0.008123799	0.017108043	0.010364083
0.022530692	0	0	0.034385685	0.016782924
0.026430258	0	0	0.019947063	0
0	0	0.007972767	0.043177106	0.018865727
0.014510147	0	0.023430062	0.0208886	0.022103036
0.057558306	0	0.085632179	0	0
0	0	0.043792503	0	0.000393184
0.055689307	0	0.05863808	0	0.043482334
0.007730236	0	0.018627531	0	0
0	0	0.057475971	0	0.059586378
0.026399801	0	0.036892098	0	0
0.035838153	0	0.042299635	0	0
0.059718066	0	0.035963356	0	0
0.039257841	0	0.103056762	0	0.049769424
0.032153201	0	0.040117832	0.018445475	0.019252643
0	0	0.080531084	0	0.013187648
0.055071671	0	0.033684229	0	0.015123883
0.046074269	0	0.026626627	0.001759831	0.014697962
0.034903244	0	0.041749829	0	0.026719071
0.014903051	0	0.067817477	0	0
0	0	0.058912159	0	0.008937848
0.024019853	0	0.001938969	0.00494832	0
0.067017471	0	0.028084787	0	0
0.059464054	0	0.022972497	0.007966149	0.035766445
0.064482248	0	0.064552502	0	0.018847378
0.004900874	0	0.038796224	0	0.008363804
0.00708215	0	0.037812638	0.047936403	0.023306597
0.014640068	0	0.042668829	0	0.000396478
0.025812142	0	0.004108494	0.009337693	0
0	0	0.048335553	0	0.019248321
0.011545087	0	0.005837945	0.0014787	0.026593272
0	0	0.020364754	0.030580412	0.014855854
0.035316784	0	0	0.042868359	0.019611481
0.064623447	0	0.016924187	0.007263633	0.014209008
0.024333306	0	0.08610335	0	0.038592089
0.027344695	0	0	0.003113739	0
0.04983725	0	0.044745063	0	0.006634223

0.02802577	0	0.101038654	0	0.032567626
0.004867109	0	0.015981414	0.009118725	0.021241184
0	0	0.070453783	0	0.030843671
0.006157204	0	0.031067235	0	0.036000018
0.049534819	0	0.070377867	0	0.02047526
0.066965946	0	0.014873146	0	0.010135001
0.055515267	0	0.067282719	0	0.051146175
0.071243769	0	0.02514003	0.000114408	0.000117166
0.052146387	0	0.049463642	0	0.007788345
0.000770093	0	0.027486916	0	0.002200119
0.014992315	0	0.016818176	0	0.026027329
0.011241706	0	0.010693801	0.014324114	0.033994862
0.000159822	0	0.075727856	0	0.024822695
0.022655898	0	0.01723066	0	0
0.070607116	0	0.019110276	0.005292348	0.01528127
0.029595474	0	0.026928376	0.005848242	0
0	0	0.057156949	0	0.010711545
0.079530986	0	0.041822353	0.016174146	0
0.055045929	0	0	0.06517164	0
0	0	0.038095634	0.006015391	0.038399071
0.032446685	0	0.039528965	0	0.013265149
0	0	0.016681699	0	0.020444526
0.040989354	0	0.017034173	0	0.008999809
0.009194446	0	0.021335081	0	0.002856876
0.029850509	0	0.028909732	0	0
0.057147013	0	0.006916312	0	0
0.05035236	0	0.057244011	0.011697253	0.05342913
0.043430697	0	0.040501844	0	0
0.024123874	0	0.031628517	0	0.016829071
0.022960045	0	0.030722053	0	0
0.075133049	0	0.032645278	0.003323159	0.019209486
0.070686326	0	0.01535137	0.035994667	0.032030265
0.020469421	0	0.054997881	0	0.022674218
0.012868345	0	0.035387494	0	0.002994604
0	0	0.029446219	0	0.020522536
0.028592768	0	0.032482624	0	0
0.032752384	0	0	0.036973561	0.017728926
0	0	0.050139641	0	0.011066912
0.037397122	0	0.107852911	0	0.0338463
0.027831048	0	0.039758946	0	0.012153549
0.024155962	0	0.017228869	0.004605259	0
0.031212308	0	0.017124275	0	0.009549022
0.020376514	0	0	0.03022403	0.056583261
0.016396972	0	0	0	0.002254576
0	0	0.038236423	0	0.026235578
0.020385195	0	0.042322999	0	0
0	0	0.048990599	0	0.026124602
0.052649753	0	0.010762904	0	0
0.004407927	0	0	0.021827559	0
0.032786595	0	0.012528422	0	0
0.038122854	0	0.014166817	0.010938441	0
0.060039877	0	0	0.043309313	0.005181215
0.027315423	0	0.0268661	0	0
0.040222391	0	0.046317739	0.010910106	0
0.031575333	0	0.009904348	0.004182025	0.005410026
0.025837119	0	0.031958536	0.005265183	0
0.021461598	0	0.037386161	0	0
0.031949496	0	0.094397629	0	0.009721023

0.005672872	0	0.022263048	0	0.00936656
0.045434186	0	0.046335796	0.005269216	0.02330411
0.037992617	0	0.014552583	0	0.01367556
0.033729065	0	0.075154858	0	0.016659468
0.036316005	0	0.014890101	0.021897936	0.021689007
0.025820943	0	0.022449579	0	0
0.058067443	0	0	0.032289425	0.002955559
0.062654767	0	0.02153622	0.004987703	0.0104238
0.016338669	0	0.06794234	0	0
0.03315192	0	0.028941608	0	0.018201568
0.041651693	0	0.058868995	0	0.019140428
0.044742508	0	0.025721899	0.0018518	0.032470914
0.001620656	0	0.045361469	0	0.007682085
0.024408688	0	0.024323506	0	0
0.024463911	0	0.012727723	0	0
0	0	0.026313976	0.004970276	0.001165258
0.048328807	0	0.025267625	0.001325216	0
0.011930137	0	0.029305309	0	0.016542608
0.014727472	0	0	0.023437382	0.023647841
0.020376187	0	0.02025917	0.011240561	0
0.09202972	0	0.020834192	0.045080495	0.060130981
0.003865845	0	0	0.007997661	0
0.034503951	0.004386742	0	0.033773315	0.021018586
0.000727137	0	0.071540364	0	0.034786325
0.041856182	0	0.077481647	0	0
0.051668082	0	0.04033758	0	0.020637
0.040932466	0	0.018194708	0	0
0.016197117	0	0.078184199	0	0
0.009015244	0	0.053644008	0	0.012282859
0.054405976	0	0.055941619	0	0.009410924
0.011432682	0	0.099897882	0	0.023329187
0.00086794	0	0.040958403	0	0.013822008
0.009030938	0	0.015967816	0.015821567	0
0	0	0.03016802	0	0.007042493
0.011173969	0	0.016523222	0.025620891	0.021445447
0.009843626	0	0.099391002	0	0.044047223
0.069548755	0	0	0.033448312	0.030385022
0.000790752	0	0.005181124	0.016883609	0
0.008050752	0	0.034344515	0	0.014101416
0.037854076	0	0.028231669	0	0
0.056735476	0	0.031654656	0	0.011432704
0.015711799	0	0.032328135	0	0
0.021384272	0	0.028191866	0.01052173	0.011158983
0.033944701	0	0.038125303	0	0
0.045626404	0	0.048843729	0	0.004078864
0.011991059	0	0.021061401	0	0
0.045973443	0	0.120395816	0	0
0.054321887	0	0.036805204	0.045226418	0.011571185
0.084988634	0	0.010295619	0.000568624	0.010140772
0.037517945	0	0.034648653	0	0
0.049297474	0	0.047333299	0	0.006948211
0.021782778	0	0.002760869	0.004545991	0
0	0	0.010878693	0.001699255	0.020606309
0.069537213	0	0.072521261	0	0.017620371
0	0	0.060990317	0	0.034794001
0.043332631	0	0.086236464	0	0.015383786
0	0	0.032556695	0	0
0	0	0.032333807	0	0.006866132

0.0623324	0	0.074026585	0	0.032356838
0.031110544	0	0.021214476	0	0
0.066776274	0	0.032299905	0.03255477	0.013328863
0	0	0.048579309	0	0.01506912
0.003247916	0	0.040417273	0	0
0.012147335	0	0.0185209	0.005742011	0
0.058081755	0	0.024060943	0	0.011620614
0.03331837	0	0.021363449	0	0.063665461
0.048475863	0	0.028936282	0	0
0.010357318	0	0.080044672	0	0.03209817
0.019954117	0	0.015512904	0.013267018	0
0.127470478	0	0.019945082	0.017627918	0.022897861
0.044573325	0	0.010958688	0	0.006782245
0.029747248	0	0.025887465	0.028843425	0.01360179
0.097368169	0	0.00064821	0.019573723	0.015915783
0.069785656	0	0.016698851	0.00933355	0.016559514
0.026163496	0.028051716	0.01024652	0	0
0.067940403	0	0.023811779	0	0
0.055392775	0	0.036865537	0	0.023428019
0.062683618	0	0.016286541	0.037831188	0.007788954
0	0	0.020477124	0	0.067709935
0.046182658	0	0.046900001	0	0
0.063947726	0	0.021675393	0.000937413	0.027291673
0.01163697	0	0.021460565	0.037243398	0.011103195
0.034172136	0	0	0.016170065	0
0.001336098	0	0.022396196	0.003417758	0
0	0	0.075784293	0	0
0.014271168	0	0.025452973	0	0
0	0	0.062600404	0	0.015092573
0.010079665	0	0.080465383	0	0.026574286
0.025968396	0	0.040848567	0	0
0.023625645	0	0.007195005	0.004182623	0
0.004446823	0	0.08361894	0	0.036701662
0.001322562	0	0.021316802	0.006181012	0.121639374
0.058221564	0	0.033373637	0	0.01849142
0.020583	0	0.044754435	0	0.050346792
0.063967427	0	0	0.025897367	0.028396418
0.05041551	0	0.007627614	0	0.006201652
0.017316713	0	0.051212918	0.002141489	0.008226096
0.035840489	0	0.038166316	0.005172802	0.015145373

Macrophages M0	Macrophages M1	Macrophages M2	Dendritic cells resting
0.026282906	0.03988693	0.106343537	0.001186772
0	0.04632231	0.297627792	0
0.067358288	0.058996609	0.222029016	0
0.288231333	0.017396105	0.321635178	0
0.126599845	0.099236214	0.066206454	0.00094632
0.01384261	0.043728355	0.303021207	0
0.156222736	0.062060013	0.13346125	0
0.283619955	0.071263931	0.076210898	0
0.340206936	0.040979317	0.260528704	0
0.171770741	0.061140456	0.319310006	0
0.108562149	0.118125579	0.189401408	0
0.318205223	0.009407225	0.222730682	0
0.066986211	0.144169853	0.166756667	0
0.020321848	0.062802231	0.153582141	0.00807116
0.496836982	0	0.012425787	0
0.18037635	0.040052344	0.194716091	0
0	0.116611319	0.164774082	0.007361358
0.273409824	0.046453645	0.081748245	0
0.117134589	0.124780274	0.060277607	0.007252525
0.265781555	0.082688805	0.145381993	0
0.152980659	0.029690429	0.219387477	0
0	0.072233608	0.2668882	0.043431057
0.206314751	0.05123291	0.275594315	0.028963772
0.270790085	0.072904577	0.202847492	0
0.132174126	0.080359231	0.121461487	0
0.463870115	0.05844469	0.128010415	0
0.147665931	0.066661231	0.221296298	0.005639146
0.235929849	0.055999676	0.236451013	0
0.174322591	0.043990723	0.130476376	0
0.29328018	0.094904169	0.117527974	0
0.237410425	0.04608108	0.266659296	0
0.432375423	0	0.056034104	0
0.046516642	0.071404331	0.209303422	0
0.151207717	0.059587404	0.163404734	0
0.053957502	0.078576085	0.217121302	0
0.004868328	0.067262076	0.12395827	0.041982511
0.341422949	0.018480257	0.194335906	0
0.513636265	0	0.031832981	0
0.209717142	0.133254934	0.074972524	0.021687624
0.056752164	0.047345737	0.305224251	0
0.217963585	0.058124363	0.117216765	0
0.051448091	0.078727226	0.155690662	0.0138144
0.074596922	0.125610797	0.188095643	0
0.057583478	0.077988459	0.128734764	0.028648484
0.115000865	0.073461961	0.228947779	0
0.022585794	0.124000544	0.105472299	0
0.071107223	0.09945267	0.142256254	0.00370235
0.158602818	0.041317662	0.219322406	0.013475134
0.031156563	0.103195592	0.131471058	0.019588406
0.127729082	0.078606914	0.170747796	0
0.175722703	0	0.248684489	0
0.072342861	0.075835485	0.226374944	0.010039839
0.022808954	0.073035596	0.129521982	0
0.042836987	0.135902088	0.003526018	0.007847914
0.312040893	0.035665338	0.193612685	0
0	0.101678315	0.341199806	0
0.078220322	0.157804497	0.158810015	0.003702172

0.152113354	0.063689304	0.25572197	0.001085037
0.066710565	0.11977358	0.185252323	0
0.079992996	0.105683967	0.191664325	0
0.076222708	0	0.194326783	0.009493116
0.107469083	0.09769781	0.012223466	0.008928034
0.070312383	0.133746199	0.217416024	0.017060599
0.21178942	0.075718679	0.100018743	0.003910859
0.10448514	0.005710182	0.035740191	0
0.265926428	0.058144734	0.2384569	0
0.44734203	0.04671238	0.196168588	0
0.147004697	0.007341091	0.249196381	0.065315579
0.305296921	0.064470346	0.115526337	0
0.199484084	0.054259043	0.187960075	0
0.014761089	0.067872127	0.531836332	0
0.229135862	0.094824128	0.106995781	0
0.360380597	0.033114464	0.276030501	0
0.14095582	0.121774987	0.113891137	0
0.32979457	0.026513929	0.11855514	0
0.097906798	0.130215667	0.081871945	0.01167981
0.015815529	0.070211877	0.091013645	0
0	0.045185626	0.316520599	0
0.080989833	0.098610594	0.121094306	0
0.296330487	0.063700075	0.245438147	0
0.041192453	0.072174174	0.101301732	0.001910824
0.265474662	0.062523324	0.061564601	0
0	0.11881332	0.149793146	0
0	0.037844417	0.188428329	0.05131213
0	0.035688965	0.15982975	0.026099782
0.128634259	0.046051361	0.141019348	0.056424288
0.187324496	0.016488426	0.153670537	0.024781395
0.174818373	0.081718009	0.180199478	0
0.389359338	0.031543852	0.22194826	0
0.174805649	0.036678577	0.437214857	0
0.280590897	0.067887309	0.129385136	0
0.066989585	0.107278305	0.081784411	0
0.078838899	0.087649327	0.307092757	0.02425687
0.35758285	0	0.162675479	0.001425991
0.13520539	0.036828248	0.336420583	0
0	0.055357614	0.136596176	0.007583006
0.091777186	0.112915077	0.160682808	0.008101328
0.062468974	0.100709531	0.090839721	0.000174547
0.370786861	0.058710184	0.205061739	0
0.188516942	0.011487082	0.221356943	0.047593776
0.160553323	0.049241533	0.211480665	0
0.06102485	0.141619122	0.094875783	0
0.092933467	0.083201217	0.136417431	0
0.211981419	0.057262079	0.325059564	0.013887184
0.359411573	0.012940121	0.300062415	0
0.131121738	0.023283127	0.31503458	0.044423927
0.444554141	0.015176759	0.168517754	0
0.349434591	0	0.109565816	0
0.115861509	0.1073502	0.076807929	0
0	0.078868019	0.239968446	0
0.088004309	0.044671572	0.037400729	0
0	0.072758973	0.333505149	0
0.417517407	0.020932198	0.022745813	0
0	0.066687487	0.143986365	0
0.075076532	0.082367042	0.180984924	0.004176787

0.018585228	0.058942477	0.279949096	0.04090579
0	0.062235785	0.212792504	0
0	0.033674808	0.170663488	0
0.077007126	0.047072502	0.192010074	0.004054361
0.437571226	0.039356508	0.011494169	0
0.249075778	0.069302316	0.177564651	0
0.200329914	0.096245137	0.004395622	0
0.029080297	0.031351571	0.250906402	0.009015116
0.034647367	0.132296923	0.273831848	0.003172213
0.122609268	0.076706695	0.109334659	0.001272715
0.240101932	0.031850997	0.25970661	0
0	0.09897551	0.177216462	0.002407584
0.035690752	0.088625497	0.148941377	0
0	0.063130315	0.260549378	0.017756824
0.270197915	0.005814922	0.267042706	0.032332962
0.306185194	0.110326914	0.059178568	0
0.151120964	0.040126112	0.128977665	0
0.391520338	0.060554307	0.139618201	0
0.132500501	0.107303574	0.23795518	0
0.378199302	0	0.150281216	0
0.450801716	0.064385815	0.139940896	0
0.442034982	0.027293685	0.146313888	0
0.389708986	0.020585092	0.111681066	0
0.127497396	0.114099306	0.144055593	0
0.423677376	0.024433507	0.264936735	0
0.298916083	0.02376023	0.144203945	0
0.049484892	0.086941903	0.158575358	0
0.066620873	0.085602855	0.150483543	0
0.073963595	0.102586855	0.120391723	0.007751914
0.187524203	0.050150493	0.048208682	0
0.555694643	0.008585214	0.000478207	0
0.330682903	0.025887598	0.205163862	0
0.185898456	0.122057801	0.140430242	0.00164988
0.16337428	0.115242359	0.07158827	0
0.295404004	0.063245899	0.121483696	0
0.053607277	0.090511602	0.127349564	0.014904101
0.334868992	0.105678542	0.227229664	0
0.308540804	0.08923449	0.097554481	0
0.277330157	0.100369059	0.063998588	0
0.146762888	0.017127251	0.317715083	0
0.052720463	0.122736561	0.132736262	0.003624071
0.032698589	0.084057908	0.173709617	0
0.085035685	0.049553296	0.317735664	0.094025633
0.085006307	0.149854449	0.078553085	0
0.395138538	0.073994297	0.134314773	0
0.373493569	0.043131346	0.220716741	0
0.099844771	0.082215365	0.095476369	0.009174787
0.17048835	0.152977262	0.040100875	0.007261829
0.24021725	0.051186997	0.156672524	0.03258407
0.003216833	0.03279386	0.046580839	0
0.086140049	0.055011914	0.275685509	0
0	0.101309296	0.10916392	0.004742882
0.186750784	0.038781368	0.224121005	0
0.083139579	0.079483976	0.178432406	0.002266567
0	0.055442192	0.171775254	0.023297767
0.09692118	0.072184981	0.053268397	0
0.045568467	0.048954853	0.17003161	0
0.049847377	0.040492186	0.100002137	0.011869301

0.046070581	0.101287698	0.119378672	0
0.023877794	0.044633944	0.109842294	0
0.034393323	0.097649641	0.158500881	0.006144981
0.333462425	0.033889931	0.322389731	0
0.37198189	0.074895472	0.173043659	0
0.030805201	0.120150655	0.117918667	0.004911703
0.012508307	0.113303245	0.175244007	0
0.26893967	0.016535641	0.168719572	0
0	0.071500654	0.122521382	0.004367008
0.21342404	0.095120477	0.15374478	0.016770508
0.204566679	0.103313556	0.096818345	0
0.409890517	0.016012137	0.16094646	0
0.142428174	0.078991035	0.090626659	0
0.553419982	0.023342458	0.171227017	0
0.194001631	0.057666314	0.203366276	0
0.409889839	0.039309214	0.095404152	0
0.089193845	0.078897451	0.222651641	0
0.620741986	0.024466513	0.142296036	0
0.123097461	0.065113728	0.186813171	0.002368474
0.021193378	0.067973707	0.141799253	0.009586489
0.042723653	0.110791144	0.050529137	0
0.034267129	0.055233994	0.099378952	0.006311956
0.214959342	0.042797429	0.1973913	0
0.029233372	0.109449795	0.125802405	0.002038554
0.200700544	0.09100819	0.086808175	0
0	0.083437152	0.093628716	0
0.197923644	0.067114583	0.108701868	0
0.1666339	0.137671626	0.053085985	0
0.240598194	0.033188669	0.344461901	0
0.169599215	0.074647289	0.061575687	0
0.257336128	0.074104784	0.243963359	0
0.006895659	0.100198515	0.092331255	0
0.146524185	0.149810732	0.022625604	0
0.072549967	0.053137726	0.293088707	0
0.016996034	0.119697676	0.147272821	0.004384926
0.150254496	0.073668693	0.161103056	0.022598655
0.12794815	0.0772925	0.179171945	0
0.008402579	0.075067592	0.115282723	0.019524811
0.063462423	0.070743741	0.107121808	0.004265785
0.229995998	0.065173798	0.101651178	0
0.09750088	0.18617162	0.101253599	0
0.37140885	0.006672143	0.233176046	0.004178958
0.036753089	0.060834804	0.20447457	0
0.183643417	0.067001906	0.148205075	0
0	0.074058995	0.119953328	0.01987184
0.260850404	0.096176185	0.181306978	0
0.29899152	0.077178901	0.075013787	0
0.675698227	0	0.021373849	0
0.03025652	0.025163589	0.183797934	0
0.066291858	0.150697989	0.070076406	0.018595823
0.310311105	0.031148133	0.080068214	0
0.200526135	0.036463493	0.240895194	0
0.065474444	0.103953522	0.096779021	0.015744242
0.089412481	0.044144065	0.133381899	0
0.112935571	0.086178306	0.116899984	0
0.308171791	0.051672531	0.274430568	0.00562057
0.325851223	0.085371633	0.217361531	0
0.165638468	0.156622993	0.034737978	0

0.409213549	0	0.12015702	0
0.028130452	0.052721683	0.192552908	0.004952215
0.310842873	0.069853275	0.213721473	0
0.137891353	0.099962678	0.316962765	0
0.112681295	0.097263328	0.105855268	0
0.129125147	0.065860613	0.191440472	0.030320942
0.246454955	0.064297299	0.061912104	0.01191764
0.212895176	0.088711512	0.120821359	0
0.399161303	0.038609359	0.357931046	0
0.272721093	0.109084263	0.208166489	0
0.04024101	0.21829155	0.186217665	0
0.319514189	0.049951477	0.238876984	0
0.227611531	0.108994076	0.097750708	0
0.339571199	0.061926349	0.188647789	0
0.163358672	0.077105912	0.035361672	0
0.004981602	0.113412081	0.119561719	0
0.276734442	0.065374638	0.084728397	0
0.222084757	0.047689911	0.141782024	0
0.299928111	0.041577327	0.195980174	0
0.063376261	0.160751262	0.106667126	0.022876298
0.042411198	0.138187304	0.155327623	0.000843261
0.193154266	0.038591039	0.2545842	0.017495089
0.15722423	0.149275775	0.074337549	0.003013492
0.434956259	0.013364576	0.276066282	0
0.07621416	0.146839584	0.128415788	0
0.197600259	0.087576411	0.096918565	0.002118924
0.564764483	0	0.122165431	0
0.227837233	0.08484249	0.088907951	0
0.153566623	0.131246783	0.012046134	0
0.240434588	0.016487937	0.264362448	0
0.066175203	0.10237074	0.181850851	0
0.335871501	0.039352785	0.138340786	0
0.341911933	0.043365513	0.191333387	0
0.235739118	0.089418235	0.210183401	0
0.023943239	0.140523913	0.037724627	0.001063661
0.060343776	0.179719031	0.103128105	0
0.068087847	0.111060019	0.251640059	0
0.137682392	0.028600034	0.152314265	0
0.042909237	0.080356904	0.134015077	0
0.075759405	0.063517646	0.101661175	0
0.287408766	0.136522856	0.13328372	0
0.113138708	0.117872701	0.121339095	0.003165185
0.524680598	0.044018283	0.095022939	0
0.23370904	0.041225883	0.352545532	0
0.183650356	0.131292072	0.085484105	0
0	0.104650716	0.243642894	0.002842911
0.254008567	0.105612415	0.093430217	0
0.089046125	0.118876153	0.145822117	0.004482522
0.460058036	0.098282077	0.061462981	0
0.460883398	0.011630633	0.124675373	0
0.174801193	0.103333316	0.179928076	0.002020245
0.025935944	0.055103367	0.495868613	0
0.139235486	0.08645571	0.070610705	0
0.030093917	0.099380258	0.048904112	0.000975285
0.051138402	0.149409869	0.082105319	0.006132202
0.035416264	0.108416194	0.050741012	0.003711521
0.422296024	0.068962838	0.211429875	0
0.098784203	0.173225664	0.17078998	0

0.046569607	0.14841219	0.059334863	0.000820165
0.2892783	0.073098503	0.172368574	0
0.088736691	0.047696062	0.065251683	0
0.259574663	0.092872643	0.049047016	0.001619437
0.094860296	0.057319096	0.149686334	0
0.097190675	0.144961747	0.084340483	0.049138305
0.090228075	0.107608397	0.023833649	0.005623079
0.294967107	0.069346067	0.13830803	0
0.213274017	0.074201201	0.128945181	0
0.120786675	0.06923358	0.172886057	0.008523122
0.020376332	0.087522183	0.180132698	0.000413643
0.095926789	0.080327157	0.105298694	0.000369372
0.020556844	0.157412165	0.172861824	0
0.256990926	0.047923385	0.265195932	0.005532837
0.271297058	0.103276778	0.139656868	0.001786266
0.290145786	0.043115702	0.18012098	0
0.495583395	0.017769008	0.112177827	0
0.548095389	0	0.034257352	0
0.334163209	0.132013631	0.098500355	0.012848312
0.05335548	0.101498726	0.048460815	0.007177908
0.177202962	0.078792671	0.171288756	0
0.000190182	0.097481982	0.085829678	0
0.280938238	0.093822075	0.126432553	0
0.195097125	0.145040237	0.173548812	0
0.30108501	0.001404425	0.449091459	0
0.370034585	0.026720118	0.165409853	0
0.16021381	0.101929764	0.04497469	0
0.334344055	0.051627046	0.169518895	0
0.028519488	0.134315692	0.078208318	0
0.300038732	0.128743387	0.265603721	0
0.026540022	0.045392247	0.164977272	0.000305379
0.096289002	0.130514146	0.128944387	0.001821462
0.268697262	0.08679203	0.082881237	0.003215825
0.185145625	0.122411583	0.222423745	0
0.012808087	0.182495337	0.074587333	0.021347678
0.230143265	0.051924887	0.264099178	0
0.027458768	0.155071067	0.212977838	0.116752967
0.239968794	0.067771662	0.186386169	0.097074874
0.312725664	0.048222721	0.04127671	0
0.094218495	0.100398408	0.142149641	0
0.314762541	0.026954468	0.29880532	0
0.272906365	0.030349098	0.296693177	0.044172918
0.180773372	0.082774651	0.273323208	0.003065745
0.394201652	0.031903188	0.26712699	0
0.474759513	0.060618763	0.04170539	0
0.236196662	0.161835426	0.073778437	0
0.079947066	0.090380774	0.094463421	0
0.271168354	0.071213864	0.245754665	0
0.168264043	0.01142667	0.449273407	0
0.343627792	0.048969502	0.209480356	0
0.36275295	0.094572715	0.071203218	0
0.303850853	0.062762817	0.082358852	0
0.472218547	0.021361048	0.20741475	0
0.186033481	0.121652886	0.068167439	0
0.413666132	0.126132617	0.129908767	0
0.511988222	0.003464053	0.196784629	0
0.351366449	0.050834157	0.214366276	0
0.042593864	0.218316865	0.123805485	0

0.032684867	0.045951574	0.039512218	0
0.079256407	0.174622833	0.040113494	0
0.580205743	0.0727022	0.08331187	0
0.080348495	0.088478957	0.046752975	0
0.114001778	0.171430422	0.087844977	0.002859189
0.64355073	0.043898779	0.096366137	0
0.083238231	0.064243286	0.082942906	0.042359224
0.217400076	0.146123049	0.069014513	0
0.526723269	0.015098013	0.092335532	0.006884749
0.40461721	0.018637547	0.168302201	0
0.054522658	0.08565238	0.103944231	0.006179637
0.303374344	0.083017699	0.034471686	0
0.12602479	0.062191949	0.069874201	0
0.452992121	0.038313039	0.194248716	0
0.431043737	0.031009547	0.232280686	0
0.409435221	0	0.197215857	0
0.387402328	0.113321941	0.087598424	0
0.107100878	0.096089468	0.079723023	0
0.13508106	0.141173982	0.179889799	0.004736823
0.488375239	0.012026538	0.024785585	0
0.095007572	0.097538587	0.052859983	0.002961173
0.379536045	0.057960515	0.087449451	0
0.396487175	0	0.201106228	0
0.058428154	0.0831489	0.089616301	0
0.50528657	0	0.040335527	0
0.024713989	0.064293602	0.072643198	0
0.471210568	0	0.16402773	0
0.329192628	0.133862826	0.061552478	0
0.30485478	0.164300585	0.055037866	0.002750716
0.143539499	0.142511653	0.083684245	0.001308786
0.057471525	0.234732577	0.118342064	0
0.161789097	0.091360451	0.108876495	0
0.282507994	0.091261823	0.133978215	0
0.148086667	0.025069653	0.076382662	0
0.217015761	0	0.379543223	0
0.141840674	0.08314824	0.09882835	0
0.090550701	0.07660043	0.092158679	0.002952167
0.348904525	0.128956842	0.066831371	0
0.028177639	0.031112139	0.069087728	0
0.524110336	0.063833747	0.121584507	0
0.070607046	0.031505952	0.062006741	0
0.595286465	0.009831493	0.125427353	0
0.106609237	0.14647506	0.155486682	0
0.418355022	0.06022214	0.216428611	0
0.319965037	0.159476403	0.079698429	0
0.563742781	0.03233097	0.164519732	0
0.220030991	0.054659622	0.024385798	0
0.12589474	0.185115995	0.038680057	0
0.069693817	0.031697222	0.213090631	0
0.444473293	0.080453212	0.149826427	0
0.532279414	0.023140419	0.064471918	0
0.272416915	0.052388747	0.301594375	0
0.015673102	0.063507897	0.211118547	0.010948134
0.153999636	0.162516205	0.064853099	0
0.314389681	0.095726492	0.10062151	0
0.116994165	0.095803872	0.076369758	0.006402452
0.374634909	0.057736495	0.146916968	0
0.12219593	0.077061256	0.040400947	0

0.050713884	0.13865075	0.030481291	0.003009631
0.384885557	0.046788498	0.28351707	0
0.15266245	0.233180355	0.123270495	0
0.048965184	0.101126428	0.092454684	0.002631513
0.43987656	0.010815002	0.146805129	0
0.402572313	0.036108225	0.092665003	0
0.102628611	0.15892446	0.034849957	0.02846749
0.348943819	0.025169957	0.315989066	0
0.270221333	0.063806734	0.078691987	0
0.01906347	0.10832589	0.064017963	0
0.258924635	0.041999558	0.279927942	0
0.004899808	0.057715294	0.131222593	0
0.474050385	0.076389462	0.073716757	0
0.060187654	0.043767686	0.180020153	0
0.09612309	0.055624645	0.123863536	0
0.101919482	0.114439478	0.035152228	0.039936596
0.412858117	0.051350721	0.201078747	0
0.421523762	0.057326703	0.106122243	0
0.093856835	0.092093938	0.109722054	0
0.280730741	0.106599301	0.086081343	0.003683231
0.583056859	0.133968371	0.029836237	0.004636875
0.43896292	0.007902207	0.210741624	0
0.177228783	0.087581288	0.235546829	0.005500981
0.093201203	0.238305478	0.089138563	0.003500172
0.414622218	0.018448821	0.16338901	0
0.514252982	0.011484015	0.193036373	0
0.422581421	0	0.106746976	0
0.444388352	0.017552118	0.262848281	0
0.067521384	0.040334319	0.10807124	0
0.132674716	0.088330951	0.103946075	0
0.372965786	0.044110347	0.169148738	0
0.476805591	0.052519217	0.15519531	0
0.078582472	0.090234783	0.031192281	0.00117542
0.152544775	0.013885739	0.393815281	0.005046446
0.100025699	0.091079788	0.096233565	0.002067818
0.303635848	0.015454424	0.119676864	0.007780191
0.354934736	0.040250339	0.238242624	0
0.512174938	0.057032688	0.090852772	0
0.033389216	0.320606793	0.165759325	0
0.109287539	0.170179567	0.083070973	0

Dendritic cells activated	Mast cells resting	Mast cells activated	Eosinophils	Neutrophils
0.039441249	0.220590526	0	0	0
0.002761972	0.057772573	0	0	0
0	0.06895799	0	0	0
0	0.05618077	0	0	0.004894104
0.004999025	0.088948989	0	0.01659677	0
0	0.030006764	0	0	0
0.01894014	0.096629073	0	0	0
0	0.017432266	0	0	0
0	0.037909702	0	0	0
0	0.006972275	0	0	0
0.00540498	0.0424838	0	0	0
0	0.059032085	0	0	0
0	2.99E-05	0	0	0
0	0.061405129	0	0	0
0.093627177	0.05583849	0	0	0
0	0.061701214	0	0	0
0	0.075248132	0	0	0
0	0.05988869	0	0	0
0	0.118694627	0	0	0
0	0	0.011249132	0	0
0.008579009	0.032906895	0	0	0
0	0.038696862	0	0	0
0.046913483	0.056618331	0	0	0
0	0.076336114	0	0	0
0.010479973	0.03750479	0	0	0
0	0.036304186	0	0	0
0	0.068278225	0	0	0
0.006858822	0.011037709	0	0	0
0	0	0.024845829	0	0
0	0.015363957	0	0	0
0	0.050553789	0	0	0
0.040273415	0.044455461	0	0	0
0	0.049277789	0	0	0
0.025390112	0.067048426	0	0	0
0.02066031	0.055386604	0	0.009865462	0
0	0.058083944	0	0	0
0.069030461	0.025476519	0	0	0
0	0.01769927	0	0	0
0	0.184319501	0	0	0
0.014212573	0.012516337	0	0	0
0.000182263	0.024327727	0	0	0
0.031575979	0.092073105	0	0.015236424	0
0	0.034402977	0	0	0.008247602
0	0.052855596	0	0	0.008200268
0.023505711	0.011016234	0	0	0
0.016405728	0.028037737	0	0	0
0	0.079518675	0	0	0
0.001773327	0.029850608	0	0	0
0.000693895	0.084914821	0	0	0
0	0.029953041	0	0	0
0.17976878	0	0	0	0.045608728
0	0.076829969	0	0	0
0.011524613	0.016186625	0	0	0
0	0.079145267	0	0.00495454	0
0	0.040744781	0	0	0
0	0.011717706	0	0	0
0.031837063	0.017235699	0	0	0.012576795

0	0.014884869	0	0	0
0	0.026514378	0	0	0
0	0.079078897	0	0	0
0	0.084488785	0	0	0
0	0	0	0.008262721	0
0	0.058993425	0	0	0
0	0.05116425	0	0	0
0	0.209970477	0	0	0
0.018732209	0.012996592	0	0	0
0	0.041240112	0	0	0
0.115518434	0.023885903	0	0	0
0	0.042937387	0	0	0
0	0.039067418	0	0	0
0	0.02380933	0	0	0
0	0.015236279	0	0	0
0	0.006612019	0	0	0
0	0.025320967	0	0	0
0.133407531	0	0	0	0
0	0.158756925	0	0	0
0	0.031004223	0	0	0
0.011133662	0.10185158	0	0	0
0.001216836	0.087626633	0	0	0
0	0.045672054	0	0	0
0	0.057375372	0	0.003645626	0
0.158912381	0	0.007298012	0	0
0.064878802	0.008760492	0	0	0
0.045200368	0.035554331	0	0	0
0.02243041	0.070257296	0	0	0
0	0.016203825	0	0	0
0.04956647	0.092517397	0	0	0
0.027070196	0.121192692	0	0	0
0	0.083334147	0	0	0
0	0.100293214	0	0	0
0	0.018299819	0	0	0
0.01168484	0	0	0	0
0	0.063443202	0	0	0
0	0.063186431	0	0.014884792	0
0.057775657	0.026210026	0	0	0.000552248
0.009166251	0.047210133	0	0	0
0	0.036099268	0	0	0
0	0.079414008	0	0	0
0	0.033780803	0	0	0
0.005498291	0.073860298	0	0	0
0.008393338	0.041265051	0	0	0
0.063282264	0.050301779	0	0	0
0	0.022175906	0	0	0
0.022401937	0.046441832	0	0	0
0	0.073708918	0	0	0
0	0.069806406	0	0	0
0	0.048484362	0	0	0
0	0	0.044815	0	0
0.011554953	0.051108726	0	0	0
0	0.03540857	0	0	0
0.112620419	0.077029471	0	0	0.002333142
0.023760636	0.012278434	0	0	0.01259888
0	0.033706052	0	0	0
0.005559258	0.006984682	0	0	0
0.005135187	0.003786069	0	0	0

0	0.054083023	0	0	0
0.039552789	0.032481154	0	0	0
0.018690602	0.023516806	0	0	0
0.01479584	0.031756164	0	0	0
0	0	0	0	0
0	0.038158559	0	0	0
0.00490363	0.038985864	0	0	0
0.017982348	0.202937203	0	0	0
0	0.071467872	0	0.000245767	0
0.022108425	0.015606962	0	0	0
0	0.071544949	0	0	0
0	0.0507808	0	0	0
0.052804216	0.008661821	0	0	0
3.08E-05	0.014452139	0	0	0
0.008679171	0.149870517	0	0	0.000218656
0	0	0	0	0
0.043088411	0.019304881	0	0	0
0	0.022840426	0	0	0
0.018625373	0.057740352	0	0	0
0.010144678	0.07999049	0	0.00186848	0
0	0.00371576	0	0	0.008025188
0.016826702	0.078198779	0.008896486	0	0
0.014116104	0.108344685	0	0	0
0.017114441	0.084041586	0	0	0
0	0	0.014347428	0	0
0.027166454	0.042820383	0	0	0
0.001047395	0.050071696	0	0	0
0.014819131	0.040615676	0	0	0
0.027042489	0.017026666	0	0	0
0	0	0.001948008	0	0
0	0	0	0	0
0.004294946	0	0	0	0
0	0.01701515	0	0	0
0	0.033673572	0	0	0
0	0.049269411	0	0	0
0.01539146	0.001469075	0	0.012029436	0
0	0.056875758	0	0	0
0	0	0	0	0
0	0.070421733	0	0	0
0	0.077728339	0	0	0
0	0.044715415	0	0.011642354	0
0	0.028662159	0.005126577	0	0
0	0	0.01188136	0	0
0.01813107	0	0.001672173	0	0
0	0.077704779	0	0	0
0	0.105106032	0	0	0
0.017106722	0.030768918	0	0	0
0	0	0	0	0
0.02208447	0.037215164	0	0	0
0	0.473205587	0	0	0
0.031727064	0.11341182	0	0	0
0.012386892	0.018906321	0	0	0
0	0.034661094	0	0	0
0	0.066822845	0	0	0
0	0.017620838	0	0	0
0.005434919	0.037187143	0	0.003134673	0
0.008305234	0.024919123	0	0	0
0.001721678	0.009723089	0	0	0

0.148833514	0.042247613	0	0	0
0.002279235	0.055550506	0	0	0
0.001771504	0.041903967	0	0	0
0	0.050230184	0	0	0
0	0.054645363	0	0	0
0	0.052438039	0	0	0
0.018124923	0.03768582	0	0	0.00053623
0.000352692	0.125306368	0	0	0
0.02322662	0	0.012702702	0	0
0	0.120180762	0	0	0
0	0.023185567	0	0	0
0	0.034724547	0	0	0
0	0.02019508	0	0	0
0	0.0349272	0	0	0
0	0.178877345	0	0	0
0	0.068763495	0	0	0
0.012565163	0.0462068	0	0	0.00477273
0	0.033821663	0	0	0
0.002551171	0.026778845	0	0	0
0	0.028970185	0	0	0
0.003978444	0.042514528	0	0.001174048	0.001070838
0	0.040969796	0	0	0
0	0.060132403	0	0	0
0	0	0	0	0
0.004814616	0.033392437	0	0	0
0.001069588	0.012014229	0	0	0
0.00697745	0.015285031	0	0	0
0	0.0132885	0	0	0
0	0.054831179	0	0	0
0	0	0	0	0
0	0.043719379	0	0	0
0.001126725	0.060535203	0	0	0
0	0.008899233	0	0	0
0	0.135635054	0	0	0
0	0.07270806	0	0.00501432	0
0	0.051436183	0	0	0
0	0.023002154	0	0	0
0.015286665	0.036315316	0	0	0
0.006926881	0.090410742	0	0	0
0	0.019913967	0	0	0
0	0.032474696	0	0	0
0.031694244	0.060512902	0	0	0
0	0.058243934	0	0	0
0	0.010215308	0	0	0
0	0.019413437	0	0	0
0	0	0	0	0
0.004431197	0.019765949	0	0	0
0	0.001069757	0	0	0
0	0.003140279	0	0	0
0	0.0816616	0	0.006017712	0
0	0.057628726	0	0	0
0	0.08115241	0	0	0
0	0.064029029	0	0	0
0.007603871	0.091273408	0	0	0
0.044481617	0.059591731	0	0	0
0	0.038222041	0	0	0
0	0.042278723	0	0	0
0.030344081	0.025268974	0	0	0

0.121883478	0	0.001081158	0	0
0.007714204	0.01026812	0	0	0
0	0.030071719	0	0	0
0.005669228	0.038739122	0	0	0.000267553
0.004883258	0.011355372	0	0	0
0.000829523	0.052675725	0	0	0
0.011064909	0.006780532	0	0	0
0	0.031782651	0	0	0
0	0.00911205	0	0	0.008851841
0	0.024281903	0	0	0.008780506
0	0.058674231	0	0	0
0	0.032505051	0	0	0
0	0.042505926	0	0	0
0	0.064294299	0	0	0
0	0.019999277	0	0	0
0	0.042889745	0	0	0
0	0.014269694	0	0	0
0	0.015699741	0	0	0
0	0.025567358	0	0	0
0.012839741	0.020772846	0	0	0
0	0.020962449	0	0.011555639	0
0	0.03898964	0	0	0
0	0.041890191	0	0	0
0	0.103334808	0	0	0
0.015245241	0.061090641	0	0.001825399	0
0.001207041	0.049453895	0	0	0
0	0.019544209	0	0	0
0	0.008160524	0	0	0
0	0.024293896	0	0	0
0	0.081826738	0	0	0
0.049912894	0.016116365	0	0	0
0.006657145	0	0.004589457	0	0
0.0219761	0.036325922	0	0	0
0	0.044699693	0	0	0
0	0.120838456	0	0	0
0.024158683	0.054972891	0	0	0
0.021203261	0.005060687	0	0	0
0.038334227	0.03258611	0	0	0
4.54E-05	0.097001232	0	0	0
0	0.036699506	0	0	0
0	0	0	0	0
0.000321449	0.019792023	0	0	0
0	0.035296333	0	0	0
0	0.031480101	0	0	0
0	0.045721194	0	0	0
0	0.022352054	0	0	0
0.011394697	0.039065537	0	0	0
0.013458116	0.044191056	0	0	0
0	0.007773261	0	0	0
0.001433961	0.173066331	0	0	0
0	0.020017545	0	0	0
0.00144325	0.094247728	0	0	0.005531314
0	0.074657764	0	0	0
0	0.038787979	0	0	0
0.00418799	0.031991647	0	0	0
0	0.00256866	0	0.000904739	0
0	0.015445604	0	0	0
0	0.0167067	0	0	0

0	0.022661615	0	0	0
0.082022763	0.061345867	0	0	0
0.010729625	0	0	0	0
0	0.007409992	0	0	0
0	0.032280309	0	0	0
0	0.023941758	0	0	0
0	0.07702421	0	0	0
0	0.050284741	0	0	0
0	0.033393353	0	0	0
0.007508001	0.055506228	0	0	0
0	0.031461822	0	0	0
0	0.089778636	0	0	0
0	0.03502917	0	0	0
0.001584446	0.013093381	0	0	0
0	0.023806896	0	0	0
0	0.096217224	0	0	0
0	0	0.033999876	0	0
0	0.092188135	0	0	0.000257635
0	0.069605609	0	0	0
0	0.079633227	0	0.005512239	0
0	0.025061226	0	0	0
0.029523007	0.074470989	0	0	0
0	0	0	0	0
0.011642018	0.042830525	0	0	0
0	0.070600303	0	0	0
0.006994062	0.041673699	0	0	0
0	0.041443476	0	0	0
0	0.047072211	0	0	0
0	0.024203556	0	0	0
0	0.031119727	0	0	0
0.014941595	0.016032352	0	0	0
0	0.088161207	0	0	0
0	0.077214715	0	0	0
0	0.000765933	0	0	0
0.000959079	0.06223669	0	0.005436086	0
0	0.039146509	0	0	0
0	0.058141714	0	0.003957291	0
0	0.030351976	0	0	0
0	0	0	0	0
0	0.011656625	0	0	0
0	0.021056582	0	0	0
0	0.083546806	0	0	0.003470337
0	0.07041719	0	0	0
0	0.036784049	0	0	0
0.122231741	0	0.004142172	0	0
0	0.018490465	0	0	0
1.25E-05	0.020882245	0	0	0
0	0.029652248	0	0	0
0	0.076957029	0	0	0.007260836
0	0.056036561	0	0	0
0	0.034132758	0	0	0
0	0.115925366	0	0.007827559	0
0	0.078804194	0	0	0
0	0.026066393	0	0	0
0	0.034665221	0	0	0
0.018347863	0.066165875	0	0	0
0.008411454	0.046826236	0	0	0
0	0.048394014	0	0	0

0.007184009	0.002285178	0	0	0
0.019517386	0.029891925	0	0	0
0	0.011246931	0	0	0
0.01061065	0.006616029	0	0	0
0	0.0551047	0	0	0
0	0.02934771	0	0	0
0	0.005562376	0	0	0
0	0.049230909	0	0	0
0	0.07282875	0	0	0
0.005995253	0.08282699	0	0	0
0	0.043313715	0	0	0
0.015865709	0.001216894	0	0	0
0.008659028	0.000462477	0	0	0
0	0.015959608	0	0	0
0.010177141	0.00577569	0	0	0
0.021224352	0.077158722	0	0	0
0	0.047573843	0	0	0
0	0.014504352	0	0	0
0	0.100228642	0	0	0
0.052386542	0.008493253	0	0	0
0	0.068178099	0	0	0
0	0.065872969	0	0	0
0.068842354	0.101219349	0	0	0
0	0.00292966	0	0	0
0.015495093	0.008100164	0	0	0
0.019593686	0.008946887	0	0	0
0.104099488	0	0.019144539	0	0
0	0.021724738	0	0	0
0	0.019153466	0	0	0
0	0.014684123	0	0	0
0	0.04731342	0	0	0
0.004403243	0.017051958	0	0	0
0	0.04507733	0	0	0
0.000881631	0.014079043	0	0	0
0.04039629	0.040794707	0	0	0.00459901
0.00619667	0.020409763	0	0	0
0	0.027849707	0	0	0
0	0.027948393	0	0	0
0	0	0	0	0
0	0.021064279	0	0	0
0.003218767	0	0	0	0
0.007929021	0.01282527	0	0	0
0.088076561	0.034490621	0	0	0
0	0.039039751	0	0	0
0	0.027992122	0	0	0
0	0.069260881	0	0	0
0	0	0.011359654	0	0
0	0.044789585	0	0	0
0.051609128	0.022496025	0	0	0
0	0.01160126	0	0	0
0.006190271	0	0.033293858	0	0
0	0.030755755	0	0	0.002385649
0	0.025156856	0	0	0
0.071057971	0.006065364	0	0	0
0.006515653	0	0	0	0
0.012599187	0.010685748	0	0	0
0	0.058872473	0	0	0
0	0.005930954	0	0	0

0	0.020055837	0	0	0
0	0.023402575	0	0	0
0	0.026149539	0	0	0
0.016679043	0.016700365	0	0	0
0	0.039577516	0	0	0
0	0.119806662	0	0	0
0.023888434	0.026544192	0	0	0
0	0.024440866	0	0	0
0	0.003303951	0	0	0
0.009291119	0	0	0	0
0	0.053852084	0	0	7.56E-05
0.016758187	0.035225485	0	0	0
0	0.050795005	0	0	0
0	0.038431371	0	0	0
0	0.021653755	0	0	0
0	0.037791526	0	0	0
0	0.12734236	0	0	0
0.001517852	0.003568827	0	0	0
0	0.035047922	0	0	0
0	0.053902461	0	0	0
0	0.001538457	0	0	0.006091287
0.012607588	0.055117283	0	0	0
0	0.044144249	0	0	0
0	0.068086538	0	0	0.003499757
0.016401794	0.042435965	0	0	0
0.004587898	0.007296147	0	0	0
0	0.058338486	0	0	0
0	0.076917435	0	0	0
0	0.012139945	0	0	0
0	0.015197003	0	0	0
0.037572222	0.010955704	0	0	0
0	0.042289682	0	0	0
0	0.004336636	0	0	0
0	0.067604188	0	0	0.015543516
0	0.044997909	0	0	0
0.025499791	0.00964356	0	0	0
0	0.047407718	0	0	0
0	0.056074999	0	0	0
0	0.059580829	0	0	0
9.75E-05	0	0	0	0
