# Supplementary figures and images for: Comprehensive analysis of cuproptosis-related long non-coding RNA signature and personalized therapeutic strategy of breast cancer patients
Source: Front Oncol. 2022 Dec 22;12:1081089. doi: 10.3389/fonc.2022.1081089 (PMC9815178; doi:10.3389/fonc.2022.1081089)

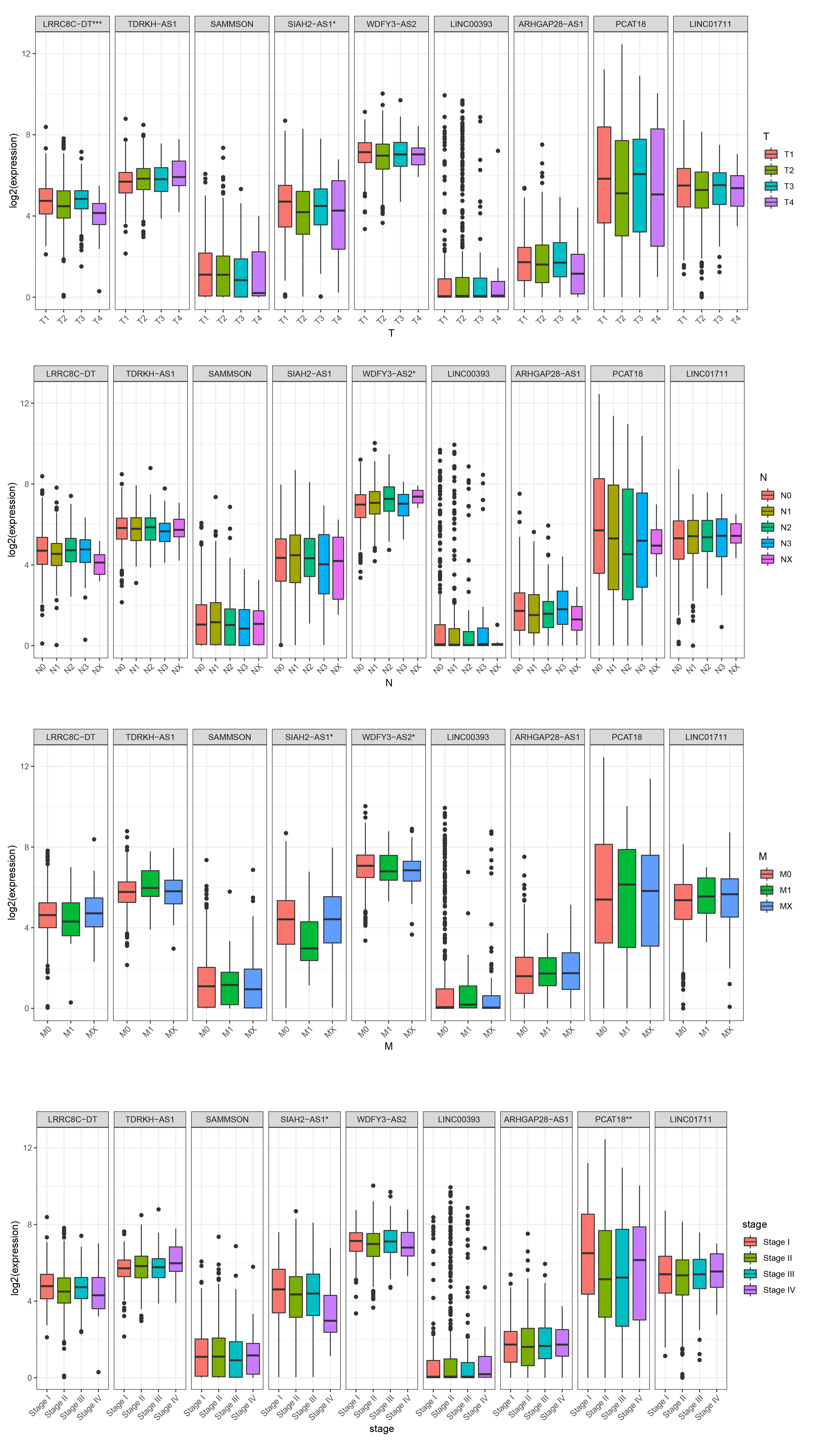

Supplement: Supplementary Figure 1 — The expression levels of the 9 cuproptosis-related lncRNAs in different tumor size and tumor stages for patients with BC. [file Image_1.tif]

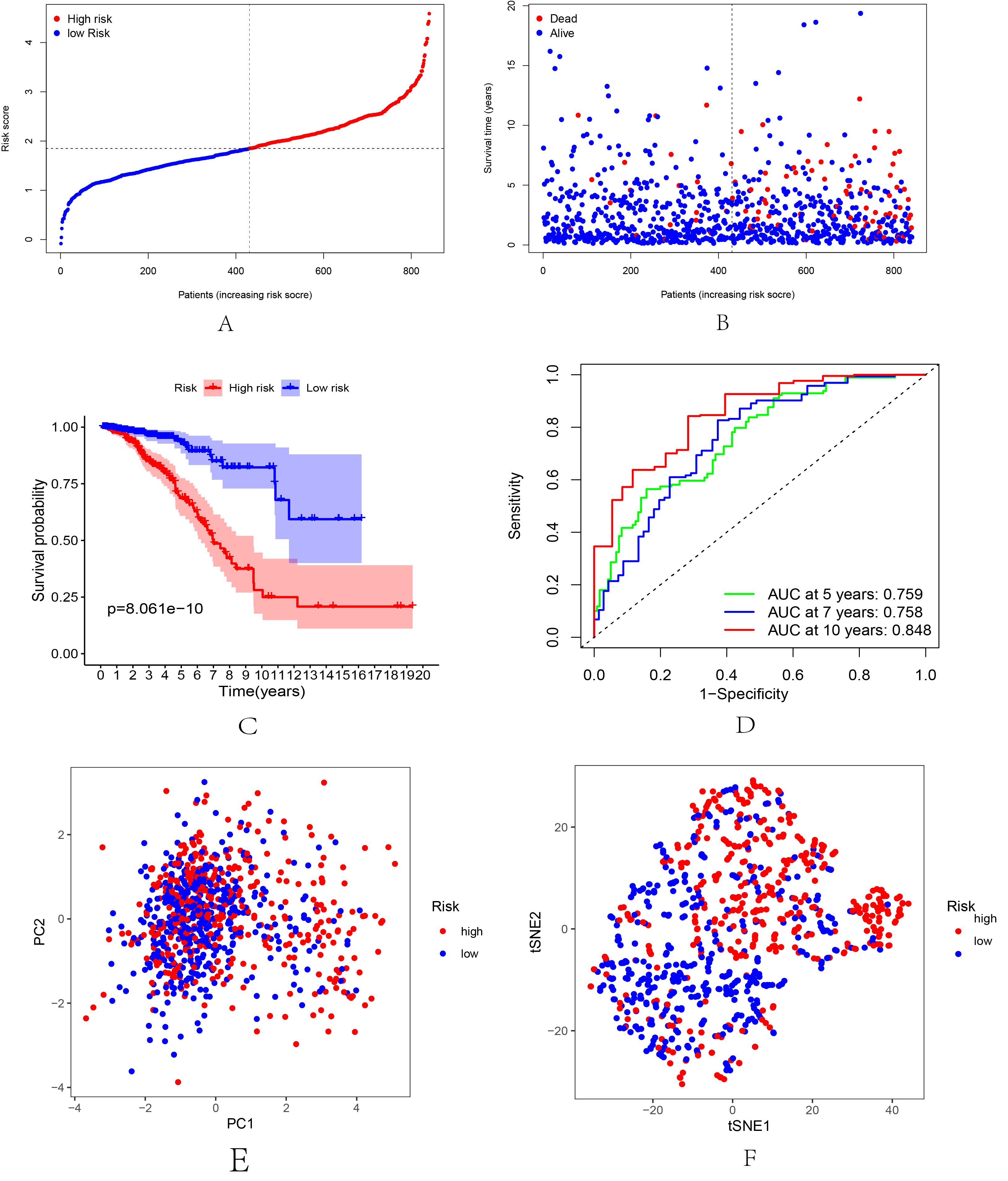

Supplement: Supplementary Figure 2 — The distribution and median risk score in whole cohort, and the cut-off value of high and low risk sets was set as the median risk score of training cohort (A, B). Kaplan–Meier survival curves for whole cohort (C), suggested that the OS of the high-risk sets was lower than that of the low-risk sets (P=8.061E-10). ROC curve analysis for the accuracy of the risk model to forecast clinical outcomes of patients with BC at 5, 7 and 10 years in the whole cohort (D). The results of PCA (E) and tSNE analysis (F) in whole cohort. [file Image_2.tif]

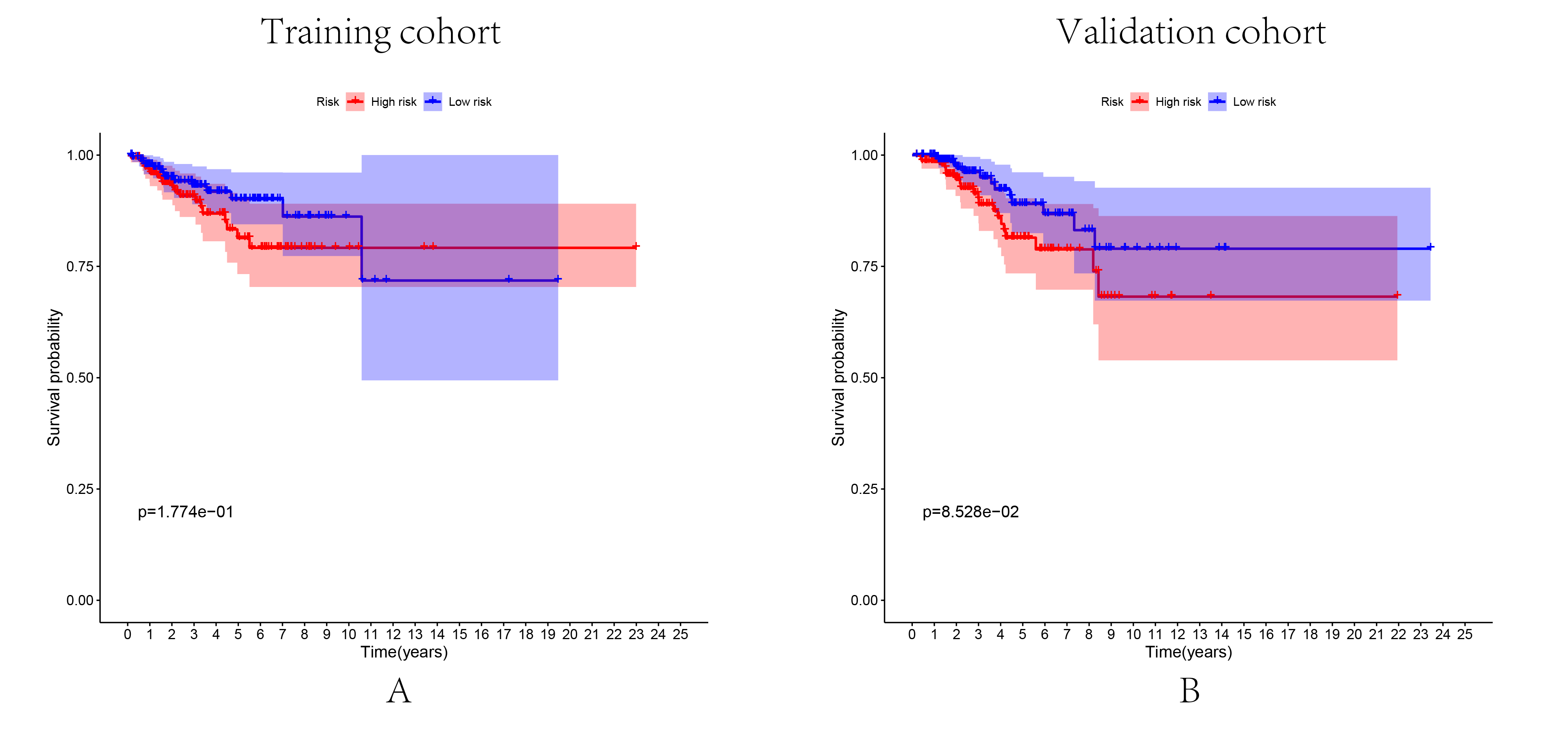

Supplement: Supplementary Figure 3 — The Kaplan-Meier analysis of PFS in training and validation cohorts. Kaplan–Meier survival curves for training (A) and validation (B) groups indicated that the PFS of the high-risk sets was lower than that of the low-risk sets (P=1.774E-01 and P=8.528E-02). [file Image_3.tif]

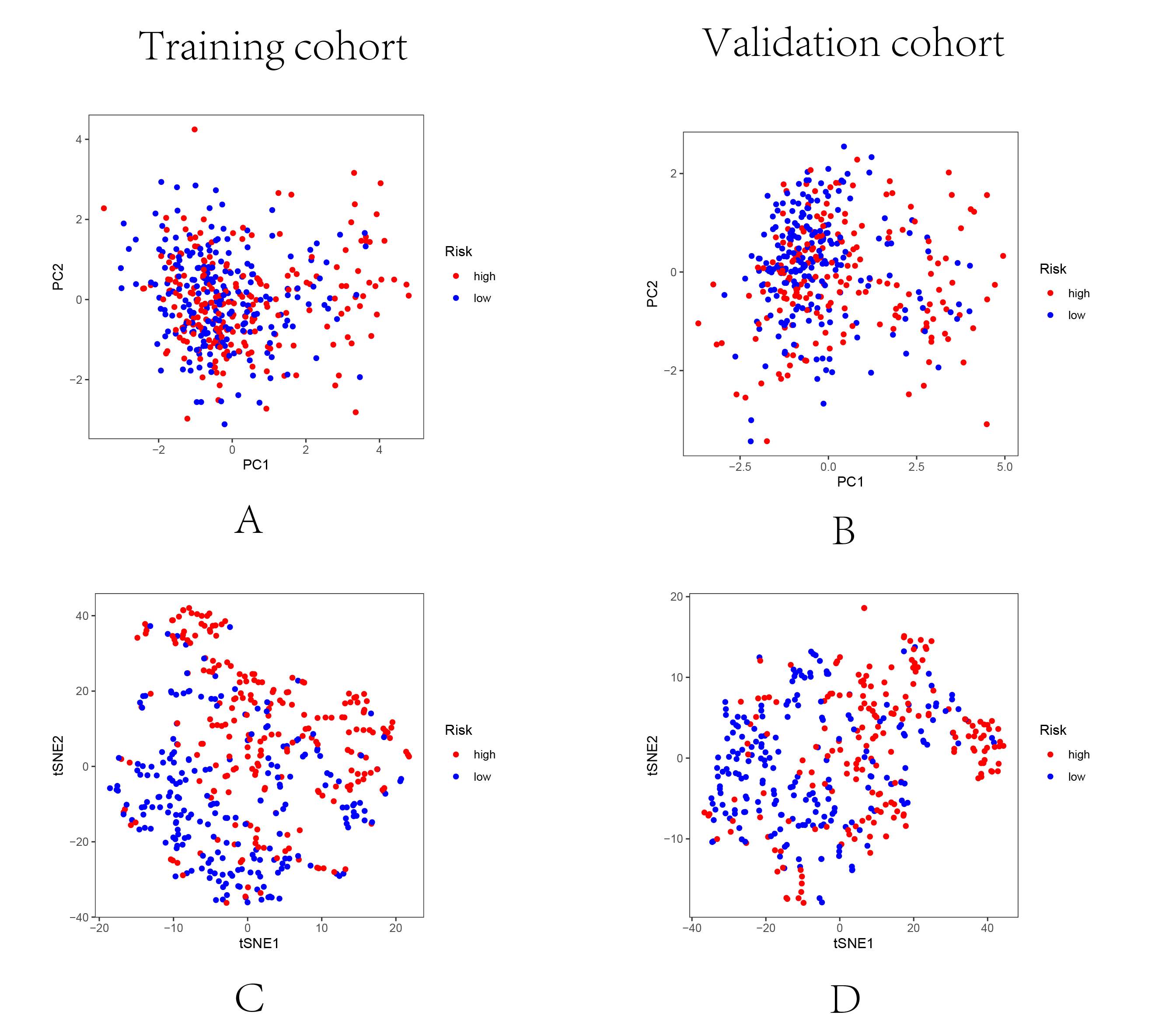

Supplement: Supplementary Figure 4 — PCA plot and tSNE plot of the training and validation groups. The patients with high risk were denoted by red points, and the patients with low risk were denoted by blue points. [file Image_4.tif]

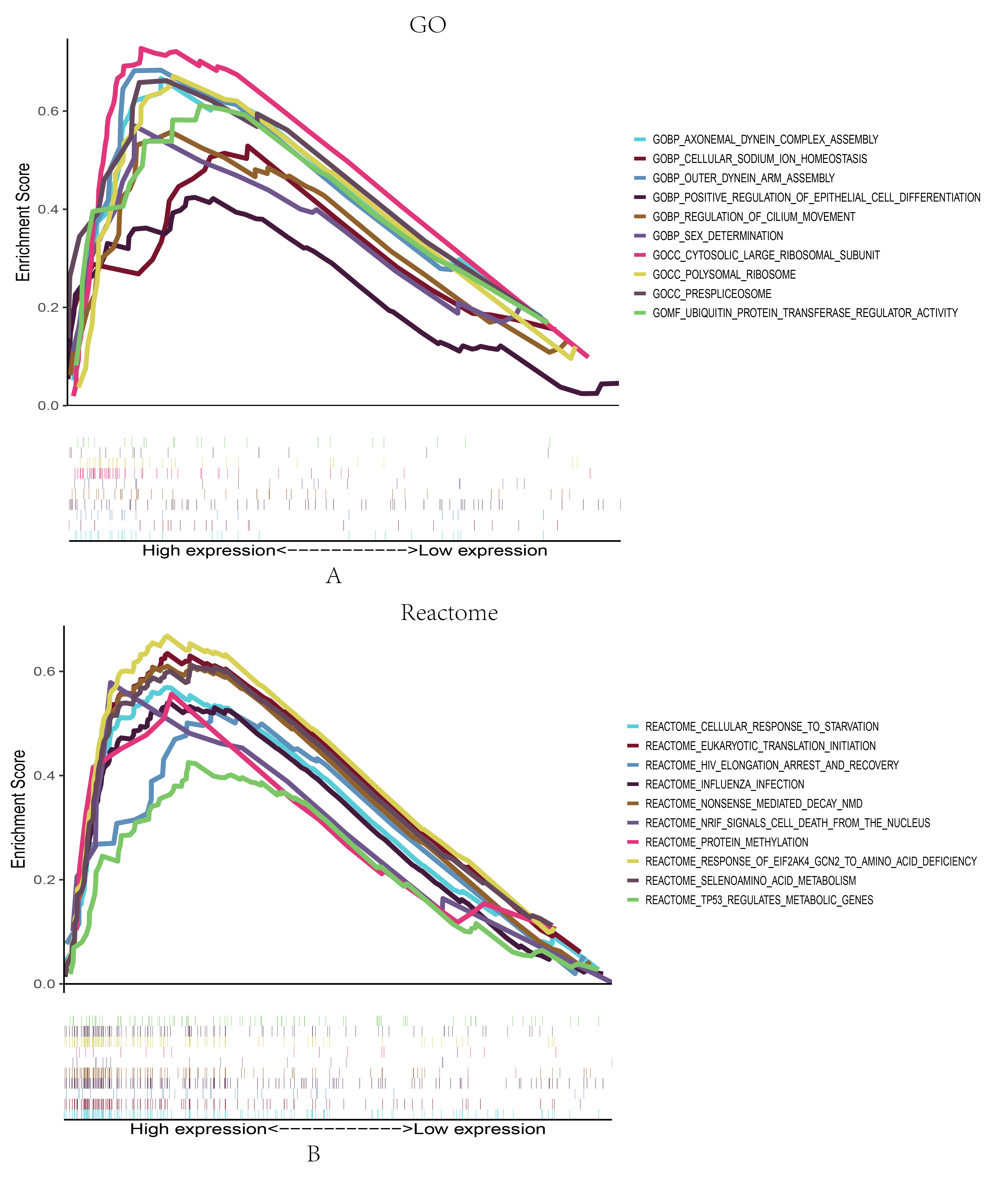

Supplement: Supplementary Figure 5 — Representative results of GO enrichment analysis and REACTOME pathway analysis in the whole cohort. [file Image_5.tif]

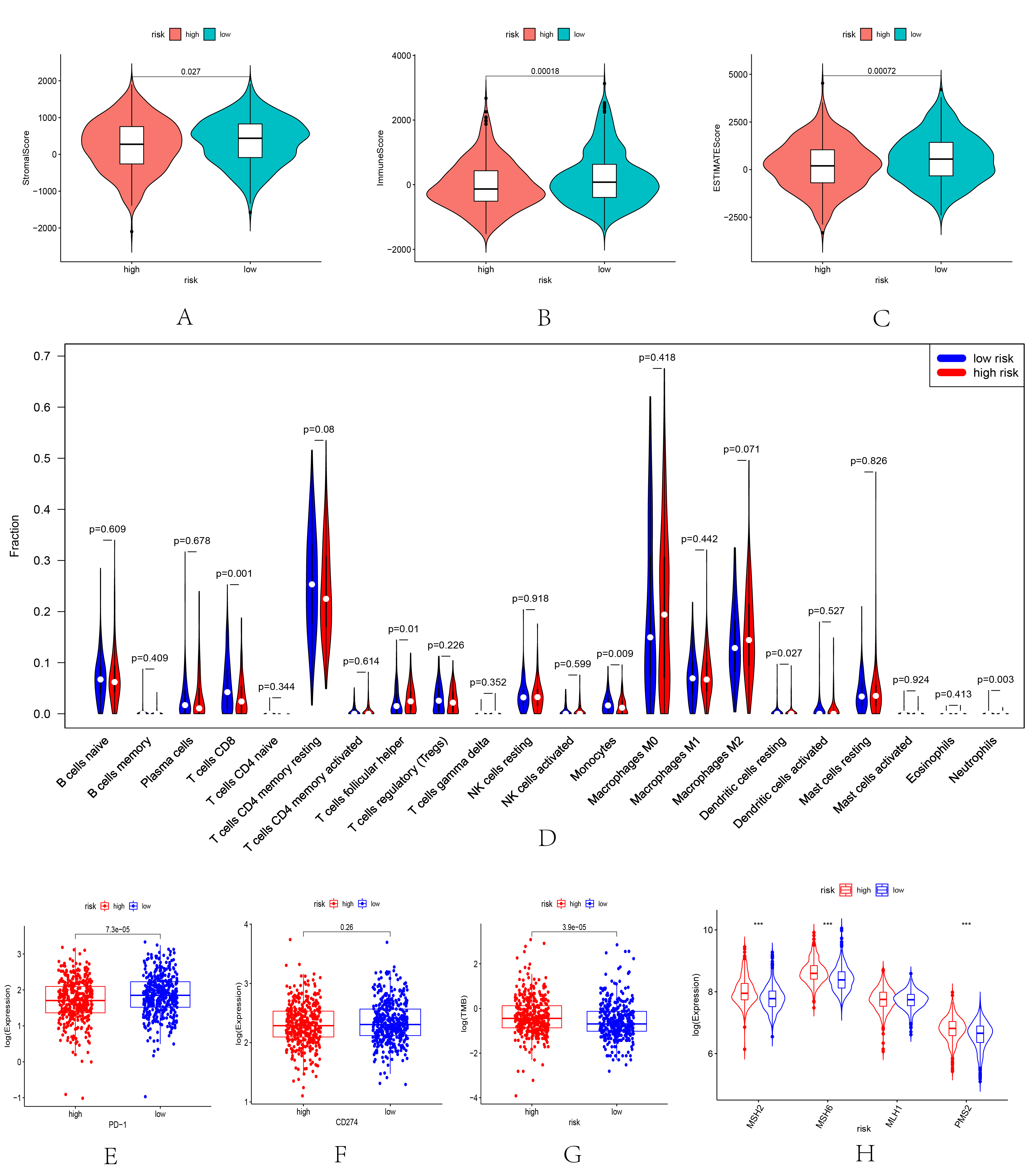

Supplement: Supplementary Figure 6 — The stromal scores, immune scores, and ESTIMATE scores of high-risk and low-risk sets in the whole cohort (A–C). The correlation of distinct different immune cells between high- and low-risk sets in the whole cohort (D), P<0.05. The high-risk sets are presented with red and the low-risk sets are presented with blue. The expression levels of immune checkpoint related genes PD-1 and PDL-1 in the whole cohort (E, F). The differences of TMB between the high- and low- risk sets in the whole cohort (G). The expression of MMR genes of BC samples in the whole cohort (H). [file Image_6.tif]
