# Supplementary material for: Comprehensive analysis of cuproptosis-related long non-coding RNA signature and personalized therapeutic strategy of breast cancer patients
Source: Front Oncol. 2022 Dec 22;12:1081089. doi: 10.3389/fonc.2022.1081089 (PMC9815178; doi:10.3389/fonc.2022.1081089)
Supplement: Supplementary file 8 [file Table_1.pdf]

LncRNAs correlated with cuproptosis

cuprotosis Gene	lncRNA	cor	pvalue	Regulation
CDKN2A	RUNX3-AS1	0.331688495	4.79E-23	postive
CDKN2A	CAMTA1-DT	0.346190451	4.35E-25	postive
PDHA1	CAMTA1-DT	0.324117683	5.06E-22	postive
CDKN2A	LINC00337	0.316583512	4.94E-21	postive
CDKN2A	PIK3CD-AS2	0.330032105	8.07E-23	postive
DLAT	DEPDC1-AS1	0.325730824	3.08E-22	postive
CDKN2A	DEPDC1-AS1	0.34910255	1.64E-25	postive
PDHA1	DEPDC1-AS1	0.384175988	5.74E-31	postive
MTF1	DLEU2L	0.391080264	4.02E-32	postive
MTF1	ERI3-IT1	0.30889419	4.74E-20	postive
CDKN2A	PRKACB-DT	0.320040408	1.75E-21	postive
PDHA1	PRKACB-DT	0.304586489	1.63E-19	postive
GLS	LINC01140	0.363199323	1.27E-27	postive
PDHA1	LINC02609	0.348281546	2.17E-25	postive
GLS	LINC02609	0.375849332	1.31E-29	postive
CDKN2A	SLC16A1-AS1	0.474585735	1.86E-48	postive
PDHA1	SLC16A1-AS1	0.331884748	4.51E-23	postive
GLS	SLC16A1-AS1	0.356900464	1.15E-26	postive
CDKN2A	LINC01356	0.478001533	3.16E-49	postive
CDKN2A	LINC01731	0.302166233	3.24E-19	postive
PDHB	DCST1-AS1	0.306817682	8.63E-20	postive
CDKN2A	ZNF687-AS1	0.356950852	1.13E-26	postive
PDHA1	ZNF687-AS1	0.41795697	6.88E-37	postive
CDKN2A	RFX5-AS1	0.404791388	1.69E-34	postive
PDHA1	RFX5-AS1	0.326889335	2.15E-22	postive
PDHA1	PCAT6	0.43502313	3.79E-40	postive
PDHA1	LINC01354	0.329225275	1.04E-22	postive
GLS	LINC01354	0.33081769	6.31E-23	postive
LIAS	CCDC18-AS1	0.307549367	6.99E-20	postive
CDKN2A	RASAL2-AS1	0.340966675	2.44E-24	postive
GLS	RASAL2-AS1	0.347273277	3.03E-25	postive
PDHA1	C1orf220	-0.31308542	1.39E-20	negative
FDX1	LEMD1-AS1	0.371707212	5.98E-29	postive
GLS	LEMD1-AS1	0.335624911	1.37E-23	postive
CDKN2A	LINC02869	0.307970476	6.19E-20	postive
PDHA1	LINC01740	0.311550479	2.19E-20	postive
CDKN2A	LEMD1-DT	0.336144152	1.16E-23	postive
GLS	LEMD1-DT	0.315472579	6.88E-21	postive
MTF1	COA6-AS1	-0.30374645	2.07E-19	negative
GLS	LINC01036	0.305545112	1.24E-19	postive
MTF1	PINK1-AS	0.325872236	2.95E-22	postive
CDKN2A	FOXD2-AS1	0.308023682	6.10E-20	postive
CDKN2A	LRRC8C-DT	0.312232064	1.79E-20	postive
MTF1	MKNK1-AS1	0.428826294	6.07E-39	postive
MTF1	PABPC4-AS1	0.386749753	2.15E-31	postive
CDKN2A	MIR9-1HG	0.337734474	6.96E-24	postive
PDHB	GAS5-AS1	0.30145953	3.96E-19	postive
CDKN2A	LINC01357	0.377227618	7.84E-30	postive
MTF1	EFCAB14-AS1	0.358936806	5.67E-27	postive

PDHB	LINC00467	0.415390709	2.05E-36	postive
CDKN2A	MED8-AS1	0.311398896	2.29E-20	postive
PDHA1	MED8-AS1	0.322825518	7.51E-22	postive
GLS	MED8-AS1	0.313371683	1.28E-20	postive
MTF1	LINC01128	0.304054457	1.90E-19	postive
PDHA1	TDRKH-AS1	0.307107346	7.94E-20	postive
CDKN2A	SNHG3	0.468887452	3.42E-47	postive
PDHA1	SNHG3	0.330037895	8.06E-23	postive
CDKN2A	SNHG12	0.556466286	1.52E-69	postive
PDHA1	SNHG12	0.323634277	5.87E-22	postive
CDKN2A	RNASEH1-AS1	0.300259035	5.55E-19	postive
PDHA1	RNASEH1-AS1	0.351005049	8.65E-26	postive
CDKN2A	LAPTM4A-DT	0.301711479	3.69E-19	postive
CDKN2A	LINC01460	0.465792337	1.63E-46	postive
PDHA1	LINC01460	0.397000626	3.90E-33	postive
GLS	LINC01460	0.363870205	1.00E-27	postive
CDKN2A	PPP4R3B-DT	0.302399627	3.04E-19	postive
PDHA1	PPP4R3B-DT	0.324973256	3.89E-22	postive
GLS	LINC01883	0.414160799	3.45E-36	postive
GLS	LINC02613	0.351115415	8.33E-26	postive
CDKN2A	LGALSL-DT	0.356715504	1.23E-26	postive
GLS	ATP6V1B1-AS1	0.33803847	6.31E-24	postive
CDKN2A	LINC01143	0.367725273	2.53E-28	postive
PDHA1	LINC01143	0.315906229	6.05E-21	postive
GLS	SIX3-AS1	0.302865461	2.66E-19	postive
GLS	TCF7L1-IT1	0.421603445	1.44E-37	postive
PDHA1	PPP1R21-DT	0.329807507	8.66E-23	postive
CDKN2A	LINC01956	0.495461431	2.67E-53	postive
PDHA1	LINC01956	0.402268999	4.71E-34	postive
GLS	LINC01956	0.362847063	1.44E-27	postive
CDKN2A	HK2-DT	0.326111901	2.74E-22	postive
GLS	STEAP3-AS1	0.312018346	1.91E-20	postive
PDHA1	PKP4-AS1	0.315372892	7.09E-21	postive
CDKN2A	THORLNC	0.309004529	4.59E-20	postive
GLS	ARHGAP15-AS1	0.339502467	3.93E-24	postive
FDX1	HECW2-AS1	0.323110957	6.89E-22	postive
CDKN2A	NCKAP5-AS2	0.327013735	2.07E-22	postive
GLS	NCKAP5-AS2	0.380846483	2.03E-30	postive
GLS	BOK-AS1	0.323861504	5.47E-22	postive
CDKN2A	SPATA3-AS1	0.411411986	1.09E-35	postive
CDKN2A	ARNILA	0.34747926	2.83E-25	postive
DLD	SOS1-IT1	0.333975807	2.32E-23	postive
DLAT	SOS1-IT1	0.36158024	2.25E-27	postive
CDKN2A	SOS1-IT1	0.352466893	5.26E-26	postive
PDHA1	SOS1-IT1	0.398906542	1.82E-33	postive
GLS	SOS1-IT1	0.313239124	1.33E-20	postive
CDKN2A	LINC00487	0.328677811	1.23E-22	postive
PDHB	ENTPD3-AS1	0.394393422	1.10E-32	postive
PDHB	CYB561D2	0.341934008	1.78E-24	postive
GLS	FAM3D-AS1	0.30097588	4.54E-19	postive
FDX1	LINC00636	0.413314807	4.92E-36	postive

CDKN2A	SAMMSON	0.340585444	2.76E-24	postive
MTF1	SUCLG2-AS1	0.335953353	1.23E-23	postive
MTF1	LINC02035	0.310832337	2.70E-20	postive
CDKN2A	ST3GAL6-AS1	0.346041933	4.57E-25	postive
GLS	ST3GAL6-AS1	0.395146618	8.14E-33	postive
CDKN2A	TIMMDC1-DT	0.375041188	1.76E-29	postive
PDHA1	TIMMDC1-DT	0.46960301	2.38E-47	postive
CDKN2A	ARHGAP31-AS1	0.320998805	1.31E-21	postive
PDHA1	ARHGAP31-AS1	0.300816814	4.74E-19	postive
GLS	ARHGAP31-AS1	0.340974832	2.43E-24	postive
CDKN2A	LINC02044	0.430212385	3.28E-39	postive
PDHA1	LINC02044	0.368913667	1.65E-28	postive
CDKN2A	LINC00881	0.349511389	1.43E-25	postive
DLAT	NCK1-DT	0.316935473	4.45E-21	postive
CDKN2A	NCK1-DT	0.40360453	2.74E-34	postive
PDHA1	NCK1-DT	0.402786981	3.81E-34	postive
GLS	LNCSRLR	0.322249223	8.96E-22	postive
PDHB	SIAH2-AS1	0.305121911	1.40E-19	postive
PDHA1	ARHGEF26-AS1	0.321311603	1.19E-21	postive
CDKN2A	TM4SF1-AS1	0.302275138	3.15E-19	postive
PDHB	DLG1-AS1	0.39654271	4.68E-33	postive
CDKN2A	MELTF-AS1	0.498466575	5.02E-54	postive
PDHA1	MELTF-AS1	0.415771149	1.74E-36	postive
CDKN2A	LINC01968	0.301864971	3.53E-19	postive
CDKN2A	ATP13A5-AS1	0.304459575	1.69E-19	postive
PDHA1	ATP13A5-AS1	0.397048235	3.83E-33	postive
GLS	ATP13A5-AS1	0.301161294	4.31E-19	postive
GLS	MBNL1-AS1	0.329828371	8.61E-23	postive
PDHA1	LINC02004	0.364682588	7.50E-28	postive
PDHB	PRKAR2A-AS1	0.446598822	1.82E-42	postive
MTF1	ARIH2OS	-0.31213101	1.85E-20	negative
PDHB	ARIH2OS	0.36857842	1.86E-28	postive
MTF1	PSMD6-AS2	0.354792132	2.38E-26	postive
CDKN2A	MYLK-AS1	0.40595046	1.05E-34	postive
PDHA1	MYLK-AS1	0.313950265	1.08E-20	postive
PDHA1	PRRT3-AS1	0.325595738	3.21E-22	postive
CDKN2A	LINC02043	0.502113317	6.47E-55	postive
PDHA1	LINC02043	0.336735083	9.60E-24	postive
FDX1	LINC01391	0.817495916	3.02E-203	postive
MTF1	ZKSCAN7-AS1	0.372447015	4.57E-29	postive
GLS	SATB1-AS1	0.321508935	1.12E-21	postive
PDHA1	THUMPD3-AS1	0.306827393	8.60E-20	postive
DLAT	H1-10-AS1	-0.33797342	6.44E-24	negative
PDHA1	LINC00504	-0.32607276	2.77E-22	negative
LIAS	LINC02482	0.336466921	1.05E-23	postive
LIAS	LINC02447	0.315962748	5.95E-21	postive
MTF1	STX18-AS1	0.309008444	4.59E-20	postive
CDKN2A	TAPT1-AS1	-0.33421222	2.15E-23	negative
PDHA1	TAPT1-AS1	-0.30408866	1.88E-19	negative
CDKN2A	HNRNPD-DT	0.461944754	1.11E-45	postive
PDHA1	HNRNPD-DT	0.348150891	2.26E-25	postive

LIAS	SNHG8	0.437147158	1.44E-40	postive
CDKN2A	WDFY3-AS2	-0.30108126	4.40E-19	negative
PDHB	BMPR1B-DT	0.337009762	8.79E-24	postive
CDKN2A	FAM198B-AS1	-0.31187216	1.99E-20	negative
CDKN2A	DANCR	0.308831348	4.83E-20	postive
PDHA1	DANCR	0.337615853	7.23E-24	postive
GLS	SAP30-DT	0.307071123	8.02E-20	postive
LIAS	UGDH-AS1	0.397463234	3.25E-33	postive
LIAS	NOP14-AS1	0.337224556	8.20E-24	postive
LIAS	STIM2-AS1	0.363654874	1.08E-27	postive
CDKN2A	AFAP1-AS1	0.42160815	1.43E-37	postive
GLS	AFAP1-AS1	0.302729674	2.77E-19	postive
PDHB	UBE2D3-AS1	0.321365006	1.17E-21	postive
CDKN2A	CTD-2297D10.[illegible]	0.321629465	1.08E-21	postive
CDKN2A	ANKH-DT	0.368542613	1.88E-28	postive
DLAT	NNT-AS1	0.315211682	7.44E-21	postive
GLS	LINC02065	0.342154672	1.65E-24	postive
PDHB	TTC23L-AS1	0.334405646	2.02E-23	postive
LIAS	EPB41L4A-AS1	0.41148273	1.06E-35	postive
CDKN2A	LINC01950	0.302266075	3.15E-19	postive
CDKN2A	LINC01170	0.326059178	2.78E-22	postive
MTF1	LINC01023	-0.34229305	1.58E-24	negative
PDHB	PPP2CA-DT	0.30758716	6.91E-20	postive
CDKN2A	MIR3142HG	0.343109826	1.21E-24	postive
PDHA1	SAP30L-AS1	-0.30826666	5.68E-20	negative
CDKN2A	KCNIP1-AS1	0.343110436	1.21E-24	postive
PDHA1	KCNIP1-AS1	0.402550231	4.20E-34	postive
CDKN2A	ZNF454-DT	0.35530594	1.99E-26	postive
CDKN2A	PRR7-AS1	0.422261496	1.08E-37	postive
GLS	PRR7-AS1	0.36073453	3.02E-27	postive
DLAT	SNHG4	0.301128914	4.35E-19	postive
CDKN2A	SNHG4	0.306352084	9.86E-20	postive
GLS	SNHG4	0.326094012	2.75E-22	postive
GLS	SH3TC2-DT	0.30342202	2.27E-19	postive
CDKN2A	LINC02159	0.378971035	4.09E-30	postive
PDHA1	LINC02159	0.396082395	5.62E-33	postive
FDX1	FABP6-AS1	0.633006988	2.29E-95	postive
CDKN2A	FOXCUT	0.336522385	1.03E-23	postive
CDKN2A	FOXF2-DT	0.369781905	1.20E-28	postive
PDHA1	JARID2-DT	0.306293238	1.00E-19	postive
GLS	LINC02521	0.311487272	2.23E-20	postive
GLS	LINC00518	0.407897397	4.71E-35	postive
CDKN2A	LINC01600	0.368422689	1.97E-28	postive
GLS	LINC01600	0.318393824	2.88E-21	postive
CDKN2A	LINC00240	0.316937452	4.45E-21	postive
DLD	LYRM4-AS1	0.33317692	2.99E-23	postive
DLAT	LYRM4-AS1	0.396738667	4.33E-33	postive
DLAT	HCG18	0.328348402	1.37E-22	postive
PDHB	ILRUN-AS1	0.329066266	1.09E-22	postive
CDKN2A	NUP153-AS1	0.543588735	7.81E-66	postive
PDHA1	NUP153-AS1	0.366161313	4.43E-28	postive

LIAS	CASC15	-0.30771279	6.67E-20	negative
GLS	BVES-AS1	0.327807854	1.62E-22	postive
GLS	FNDC1-IT1	0.359574107	4.54E-27	postive
CDKN2A	HDAC2-AS2	0.327617898	1.72E-22	postive
CDKN2A	CAHM	0.397059025	3.81E-33	postive
PDHA1	CAHM	0.317526927	3.73E-21	postive
DLAT	HMGN3-AS1	0.349242843	1.57E-25	postive
LIAS	HMGN3-AS1	-0.30502254	1.44E-19	negative
PDHA1	HMGN3-AS1	0.336778886	9.47E-24	postive
GLS	HMGN3-AS1	0.300228113	5.60E-19	postive
GLS	LINC01615	0.381432175	1.62E-30	postive
CDKN2A	FOXP4-AS1	0.401424076	6.62E-34	postive
PDHA1	FOXP4-AS1	0.40621869	9.40E-35	postive
GLS	FOXP4-AS1	0.366065861	4.58E-28	postive
PDHA1	POLH-AS1	0.378272468	5.31E-30	postive
CDKN2A	ETV7-AS1	0.404719374	1.74E-34	postive
PDHA1	ETV7-AS1	0.323630472	5.87E-22	postive
CDKN2A	LINC02570	0.307475208	7.14E-20	postive
PDHA1	LINC02570	0.315022171	7.87E-21	postive
CDKN2A	SNHG15	0.440028342	3.86E-41	postive
PDHA1	SNHG15	0.376793052	9.21E-30	postive
GLS	PPP1R9A-AS1	0.354837061	2.34E-26	postive
DLD	EMSLR	0.379883862	2.91E-30	postive
DLAT	EMSLR	0.361272131	2.50E-27	postive
CDKN2A	EMSLR	0.464129581	3.74E-46	postive
PDHA1	EMSLR	0.426395344	1.78E-38	postive
GLS	EMSLR	0.313229134	1.34E-20	postive
GLS	DLX6-AS1	0.454798659	3.64E-44	postive
GLS	LINC02830	0.327046484	2.05E-22	postive
DLD	MKLN1-AS	0.335789217	1.30E-23	postive
CDKN2A	PAXIP1-AS2	-0.31257092	1.62E-20	negative
PDHA1	PAXIP1-AS2	-0.36884277	1.69E-28	negative
PDHA1	INSIG1-DT	0.308050639	6.05E-20	postive
PDHA1	PPP1R35-AS1	0.302654096	2.83E-19	postive
PDHA1	NDUFB2-AS1	0.328606189	1.26E-22	postive
DLD	REPIN1-AS1	0.363792583	1.03E-27	postive
LIAS	LINC00957	0.316069642	5.76E-21	postive
DLD	WASL-DT	0.320809151	1.39E-21	postive
CDKN2A	SNHG26	0.42159517	1.44E-37	postive
GLS	SNHG26	0.405641731	1.19E-34	postive
GLS	TMEM139-AS1	0.312074235	1.88E-20	postive
PDHA1	MAGI2-AS3	-0.3001568	5.71E-19	negative
MTF1	KMT2E-AS1	-0.35354109	3.65E-26	negative
PDHA1	LINC01006	0.362268902	1.76E-27	postive
CDKN2A	LINC02595	0.372036271	5.30E-29	postive
GLS	LINC02595	0.343076965	1.22E-24	postive
MTF1	LINC00630	0.429198399	5.15E-39	postive
CDKN2A	LINC01186	0.338797425	4.94E-24	postive
CDKN2A	FIRRE	0.339933853	3.41E-24	postive
GLS	FIRRE	0.308635233	5.11E-20	postive
CDKN2A	PLS3-AS1	0.332517265	3.69E-23	postive

GLS	PLS3-AS1	0.389150108	8.51E-32	postive
MTF1	CH17-340M24.3	-0.30264532	2.83E-19	negative
PDHB	CH17-340M24.3	0.300528152	5.14E-19	postive
MTF1	MID1IP1-AS1	0.30250245	2.95E-19	postive
PDHA1	XIST	-0.32302317	7.07E-22	negative
PDHB	JPX	0.347405384	2.90E-25	postive
DLAT	OTUD6B-AS1	0.382515278	1.08E-30	postive
CDKN2A	PDE7A-DT	0.426989756	1.37E-38	postive
PDHA1	PDE7A-DT	0.404430673	1.95E-34	postive
GLS	CASC8	0.343438299	1.08E-24	postive
PDHA1	LY6E-DT	0.445629904	2.86E-42	postive
GLS	LINC01301	0.347698867	2.63E-25	postive
CDKN2A	CDKN2A-DT	0.796187706	2.89E-185	postive
PDHA1	CDKN2A-DT	0.385675364	3.24E-31	postive
CDKN2A	CDKN2B-AS1	0.535720339	1.21E-63	postive
CDKN2A	LINC02872	0.377722287	6.52E-30	postive
PDHA1	LINC02872	0.308056934	6.04E-20	postive
GLS	LINC02872	0.408375491	3.86E-35	postive
CDKN2A	MIR181A2HG	0.323743492	5.67E-22	postive
MTF1	ARRDC1-AS1	-0.30362263	2.15E-19	negative
CDKN2A	LINC00092	0.308348836	5.55E-20	postive
FDX1	GLIDR	0.326206413	2.66E-22	postive
LIAS	SNHG7	0.315138382	7.60E-21	postive
CDKN2A	VLDLR-AS1	0.335323106	1.51E-23	postive
PDHA1	VLDLR-AS1	0.38436756	5.34E-31	postive
CDKN2A	FAM27E3	0.406320533	9.01E-35	postive
PDHA1	FAM27E3	0.407803118	4.90E-35	postive
MTF1	TOLLIP-AS1	-0.30561904	1.22E-19	negative
CDKN2A	LINC02721	0.393084012	1.83E-32	postive
PDHA1	LINC02721	0.392584932	2.23E-32	postive
GLS	LINC02721	0.330769303	6.40E-23	postive
CDKN2A	LINC02739	0.321328532	1.19E-21	postive
DLAT	USP2-AS1	0.366190892	4.38E-28	postive
CDKN2A	USP2-AS1	0.355375337	1.95E-26	postive
PDHA1	USP2-AS1	0.379060012	3.96E-30	postive
GLS	LINC02732	0.333852838	2.41E-23	postive
DLAT	UVRAG-DT	0.310254907	3.19E-20	postive
DLAT	BACE1-AS	0.408840767	3.19E-35	postive
PDHA1	MIR210HG	0.373935102	2.65E-29	postive
CDKN2A	SNHG1	0.469361634	2.69E-47	postive
PDHA1	SNHG1	0.373577916	3.02E-29	postive
DLAT	GSEC	0.344819228	6.86E-25	postive
PDHA1	GSEC	0.336635063	9.92E-24	postive
PDHA1	KIRREL3-AS1	0.301679618	3.72E-19	postive
GLS	KIRREL3-AS1	0.340111284	3.22E-24	postive
PDHB	ZBED5-AS1	0.395824937	6.22E-33	postive
PDHA1	TMEM9B-AS1	-0.31014106	3.30E-20	negative
MTF1	CENATAC-DT	0.330699884	6.55E-23	postive
DLAT	CENATAC-DT	0.417315245	9.05E-37	postive
PDHA1	C11orf72	0.376846841	9.03E-30	postive
CDKN2A	PRKCQ-AS1	0.408679745	3.41E-35	postive

CDKN2A	VIM-AS1	0.362715824	1.51E-27	postive
PDHA1	VIM-AS1	0.319440337	2.10E-21	postive
GLS	VIM-AS1	0.421753013	1.35E-37	postive
CDKN2A	RPP38-DT	0.403671932	2.66E-34	postive
PDHA1	RPP38-DT	0.391819925	3.01E-32	postive
CDKN2A	CCNY-AS1	0.416613742	1.22E-36	postive
CDKN2A	LINC00839	0.327792862	1.63E-22	postive
GLS	LINC00839	0.313533952	1.22E-20	postive
CDKN2A	ZNF22-AS1	0.33796044	6.47E-24	postive
CDKN2A	SGMS1-AS1	-0.33805488	6.28E-24	negative
PDHB	SGMS1-AS1	0.348733183	1.86E-25	postive
PDHA1	SGMS1-AS1	-0.36661767	3.76E-28	negative
GLS	LINC00857	0.327118862	2.00E-22	postive
MTF1	LINC00863	0.339974491	3.37E-24	postive
MTF1	ENTPD1-AS1	0.372246756	4.91E-29	postive
FDX1	EMX2OS	0.396201647	5.36E-33	postive
CDKN2A	LINC01475	0.318874824	2.49E-21	postive
GLS	LINC00601	0.321206403	1.23E-21	postive
CDKN2A	TLX1NB	0.313011795	1.42E-20	postive
LIAS	C10orf95-AS1	0.357675484	8.79E-27	postive
MTF1	NEBL-AS1	-0.31155173	2.19E-20	negative
PDHA1	MIR1915HG	0.352996727	4.39E-26	postive
GLS	MIR1915HG	0.369062675	1.56E-28	postive
PDHA1	SUV39H2-DT	0.336969264	8.91E-24	postive
CDKN2A	PFKP-DT	0.446108042	2.29E-42	postive
PDHA1	PFKP-DT	0.345586635	5.32E-25	postive
GLS	PITRM1-AS1	0.378793912	4.37E-30	postive
GLS	IDI2-AS1	0.307147334	7.85E-20	postive
PDHA1	LINC02652	0.341512167	2.04E-24	postive
GLS	LINC02652	0.369088957	1.55E-28	postive
PDHA1	WAC-AS1	0.33804547	6.29E-24	postive
FDX1	MIR202HG	0.849568585	2.75E-235	postive
CDKN2A	LINC02367	0.445246838	3.43E-42	postive
CDKN2A	DDX11-AS1	0.467048205	8.66E-47	postive
PDHA1	DDX11-AS1	0.374177447	2.42E-29	postive
PDHB	BCDIN3D-AS1	0.314028887	1.06E-20	postive
PDHA1	POC1B-AS1	-0.30352956	2.21E-19	negative
GLS	LINC00943	0.319463422	2.08E-21	postive
LIAS	PXN-AS1	0.420825409	2.01E-37	postive
CDKN2A	LINC00944	0.319488688	2.07E-21	postive
GLS	LINC00944	0.302220379	3.20E-19	postive
DLAT	LINC01089	-0.32331802	6.46E-22	negative
PDHB	NRAV	0.38865359	1.03E-31	postive
PDHB	ADCY6-DT	0.377669948	6.65E-30	postive
GLS	CACNA1C-AS4	0.308330133	5.58E-20	postive
CDKN2A	LINC00393	0.30430896	1.77E-19	postive
GLS	LINC00393	0.37970647	3.11E-30	postive
DLAT	LINC01232	0.304384909	1.73E-19	postive
PDHA1	LINC01232	0.327428862	1.82E-22	postive
CDKN2A	MIR17HG	0.415728519	1.78E-36	postive
PDHA1	MIR17HG	0.328351521	1.37E-22	postive

GLS	MYO16-AS1	0.317401444	3.87E-21	postive
CDKN2A	LMO7-AS1	0.349410616	1.48E-25	postive
GLS	TPT1-AS1	0.33478977	1.79E-23	postive
CDKN2A	SPART-AS1	0.321993771	9.68E-22	postive
CDKN2A	SNHG10	0.333571855	2.64E-23	postive
PDHA1	SNHG10	0.339054505	4.54E-24	postive
CDKN2A	LINC02323	0.317909694	3.33E-21	postive
PDHA1	LINC02323	0.3175109	3.75E-21	postive
PDHB	LINC02332	0.311058072	2.53E-20	postive
CDKN2A	LINC00605	0.312949281	1.45E-20	postive
LIAS	DICER1-AS1	0.383095337	8.66E-31	postive
GLS	HIF1A-AS3	0.335642059	1.36E-23	postive
PDHA1	VPS39-DT	-0.30014531	5.73E-19	negative
MTF1	NPTN-IT1	0.353633442	3.54E-26	postive
PDHA1	NPTN-IT1	-0.30547378	1.27E-19	negative
PDHB	USP3-AS1	0.302136965	3.27E-19	postive
PDHA1	RAD51-AS1	-0.32129544	1.20E-21	negative
GLS	LINC01583	0.329431219	9.75E-23	postive
FDX1	LINC01197	0.356841465	1.17E-26	postive
GLS	CARMAL	0.378236818	5.38E-30	postive
LIAS	EIF3J-DT	0.339747922	3.63E-24	postive
MTF1	OIP5-AS1	0.338159245	6.07E-24	postive
CDKN2A	SNHG21	0.349648525	1.37E-25	postive
PDHA1	SNHG21	0.330110075	7.88E-23	postive
PDHB	WASIR2	0.304281769	1.78E-19	postive
GLS	CEROX1	0.402233078	4.78E-34	postive
PDHA1	LINC02188	0.36872063	1.77E-28	postive
GLS	LINC02188	0.370996678	7.75E-29	postive
CDKN2A	C16orf95-DT	0.339058015	4.54E-24	postive
PDHA1	C16orf95-DT	0.413033709	5.54E-36	postive
GLS	LINC02189	0.389740826	6.77E-32	postive
PDHB	CD2BP2-DT	0.441653936	1.82E-41	postive
CDKN2A	VPS9D1-AS1	0.315154475	7.56E-21	postive
PDHA1	VPS9D1-AS1	0.402314634	4.62E-34	postive
GLS	SRRM2-AS1	-0.32797849	1.53E-22	negative
CDKN2A	SNHG30	0.397538898	3.15E-33	postive
PDHA1	SNHG30	0.443222165	8.81E-42	postive
CDKN2A	LINC01563	0.305600149	1.22E-19	postive
PDHA1	LINC01482	0.313429174	1.26E-20	postive
GLS	LINC01482	0.315775181	6.29E-21	postive
CDKN2A	LINC00511	0.456011396	2.03E-44	postive
PDHA1	LINC00511	0.343021734	1.24E-24	postive
GLS	LINC00511	0.396201715	5.36E-33	postive
GLS	PRKCA-AS1	0.41203862	8.41E-36	postive
FDX1	C1QTNF1-AS1	0.518237155	5.55E-59	postive
CDKN2A	DNAH17-AS1	0.480118983	1.04E-49	postive
LIAS	LINC02693	-0.30936731	4.13E-20	negative
GLS	LINC02693	0.316841112	4.58E-21	postive
GLS	LINC01152	0.423794312	5.54E-38	postive
GLS	LINC02097	0.349218962	1.58E-25	postive
GLS	ROCR	0.366853445	3.46E-28	postive

CDKN2A	SOX9-AS1	0.363774532	1.04E-27	postive
PDHA1	SOX9-AS1	0.30065069	4.97E-19	postive
GLS	SOX9-AS1	0.451918201	1.46E-43	postive
CDKN2A	LINC01971	0.408616563	3.50E-35	postive
PDHA1	LINC01971	0.323644584	5.85E-22	postive
GLS	LINC01971	0.301664672	3.74E-19	postive
PDHB	LINC00324	0.332179437	4.11E-23	postive
DLAT	SNHG16	0.345698759	5.13E-25	postive
CDKN2A	ARHGAP28-AS1	0.302905372	2.63E-19	postive
CDKN2A	TYMSOS	0.361153372	2.61E-27	postive
PDHA1	TYMSOS	0.36383941	1.01E-27	postive
PDHA1	GATA6-AS1	0.331863792	4.54E-23	postive
CDKN2A	THOC1-DT	0.316827541	4.60E-21	postive
PDHB	ZNF516-DT	0.342878895	1.30E-24	postive
LIAS	LINC01927	0.302052196	3.35E-19	postive
CDKN2A	COSMOC	0.397372453	3.37E-33	postive
PDHA1	COSMOC	0.330705373	6.53E-23	postive
GLS	LINC01910	0.324449127	4.57E-22	postive
LIAS	ZNF516-AS1	0.319840215	1.86E-21	postive
GLS	LINC02837	0.397075307	3.79E-33	postive
PDHB	PCAT18	0.301082538	4.40E-19	postive
CDKN2A	SNHG17	0.352888284	4.56E-26	postive
PDHA1	SNHG17	0.343319704	1.13E-24	postive
CDKN2A	PELATON	0.351188739	8.12E-26	postive
PDHA1	PELATON	0.389783882	6.65E-32	postive
CDKN2A	LINC01273	0.319676788	1.95E-21	postive
DLAT	LINC01711	-0.31279052	1.52E-20	negative
PDHA1	SLCO4A1-AS1	0.303886202	1.99E-19	postive
GLS	SLCO4A1-AS1	0.319908415	1.82E-21	postive
PDHA1	MHENCR	0.333003274	3.16E-23	postive
CDKN2A	LINC01842	0.30203787	3.36E-19	postive
LIAS	LINC00663	0.310056852	3.38E-20	postive
PDHA1	LINC00663	-0.32629282	2.59E-22	negative
