# Supplementary material for: Comprehensive analysis of cuproptosis-related long non-coding RNA signature and personalized therapeutic strategy of breast cancer patients
Source: Front Oncol. 2022 Dec 22;12:1081089. doi: 10.3389/fonc.2022.1081089 (PMC9815178; doi:10.3389/fonc.2022.1081089)
Supplement: Supplementary file 9 [file Table_2.pdf]

The differential expressed lncRNAs

lncRNA	logFC	logCPM	P-Value	FDR adjusted P-Value
LINC02202	-3.020605527	7.276129689	1.73E-264	5.46E-261
HSD11B1-AS1	-4.210328511	7.117882938	1.10E-210	1.74E-207
TRHDE-AS1	-5.303622739	9.066609774	1.32E-204	1.39E-201
LINC01537	-3.328217228	6.441587168	1.46E-198	1.15E-195
LINC01697	-4.25589499	6.964845612	5.71E-194	3.61E-191
LINC02884	-3.523623948	5.08684508	3.50E-183	1.84E-180
LGR4-AS1	-2.883852104	4.821628681	5.12E-181	2.31E-178
LINC02587	-3.872149885	6.259567873	1.31E-180	5.18E-178
LINC02237	-4.950865715	4.733621204	3.96E-179	1.39E-176
CARMN	-3.27024117	9.579025474	7.58E-178	2.39E-175
ALDH1L1-AS2	-4.821898848	5.869326671	1.29E-176	3.69E-174
ADAMTS9-AS2	-2.987672607	7.259192497	4.73E-176	1.24E-173
LINC00968	-3.646568591	7.191919052	8.94E-159	2.17E-156
MIR133A1HG	-4.723158135	5.941417024	3.97E-158	8.96E-156
LINC01070	-4.557604715	4.058223221	4.92E-149	1.04E-146
LINC01230	-4.453362408	6.946487567	2.01E-148	3.96E-146
LINC02511	-4.0402198	5.09336591	8.68E-148	1.61E-145
MAGI2-AS3	-1.968195825	10.98563619	1.50E-147	2.63E-145
LINC00377	-3.697233815	3.468561872	6.14E-146	1.02E-143
ADIPOQ-AS1	-4.939962309	4.743347423	7.28E-146	1.15E-143
MESTIT1	-2.529823846	6.308238168	7.79E-146	1.17E-143
PGM5P3-AS1	-3.456161834	4.094331119	1.07E-141	1.53E-139
LINC01614	5.834025892	9.639084465	4.74E-136	6.51E-134
LINC02185	-2.617611884	5.084099714	6.32E-135	8.32E-133
LINC01485	-3.642906157	6.682800257	5.17E-132	6.53E-130
ST3GAL3-AS1	-3.993600824	3.887222139	6.06E-131	7.36E-129
MIR1-1HG	-9.868520723	6.367280055	6.36E-130	7.44E-128
LRIG2-DT	-1.827550955	5.963538774	2.26E-126	2.55E-124
LUARIS	-2.915806232	5.289896965	5.36E-126	5.84E-124
PGM5P4-AS1	-3.241390919	3.720483709	1.67E-124	1.76E-122
LINC02082	-3.579102182	3.306959337	4.87E-123	4.96E-121
KCNIP2-AS1	-2.02361954	5.06402266	1.61E-117	1.59E-115
MRGPRF-AS1	-2.836320405	3.796736786	3.44E-117	3.29E-115
PDE9A-AS1	-2.796845405	5.221708024	9.53E-117	8.85E-115
FGGY-DT	-4.165792306	3.955835363	2.43E-116	2.19E-114
OXCT1-AS1	-2.058779777	5.833515716	5.02E-115	4.40E-113
ARHGEF7-AS2	-2.784240072	3.483866867	9.28E-112	7.92E-110
RBMS3-AS3	-2.528926409	4.691200059	9.78E-111	8.13E-109
LINC02660	-3.441155364	4.28773226	1.16E-109	9.38E-108
RHOXF1-AS1	-3.104207951	8.188409444	5.74E-109	4.53E-107
DAAM2-AS1	-2.171449657	7.310738401	1.63E-107	1.25E-105
LINC01140	-2.116810727	8.533260088	3.28E-105	2.47E-103
HID1-AS1	-1.975445615	5.382070566	4.18E-105	3.07E-103
LMO7DN	-2.819980057	3.620118991	3.71E-103	2.66E-101
RBPMS-AS1	-1.886352116	7.020884868	1.03E-102	7.20E-101
ADAMTS9-AS1	-2.664589724	7.462386917	3.86E-102	2.65E-100
PARAL1	-4.004515477	5.592187745	4.42E-102	2.97E-100
ACTA2-AS1	-2.307286071	9.257269976	6.12E-102	4.02E-100
LINC00922	4.78211877	7.279955446	5.16E-101	3.32E-99

MIR497HG	-1.690567379	7.902505122	1.58E-100	9.95E-99
LEF1-AS1	2.56300294	5.919308071	3.22E-99	1.99E-97
MEF2C-AS1	-2.063455255	5.82667019	2.85E-98	1.73E-96
LINC01705	5.729688859	6.54062431	1.82E-96	1.08E-94
GAS1RR	-1.948427657	7.454113162	3.01E-95	1.76E-93
C6orf99	2.937054118	7.529894297	5.04E-95	2.90E-93
LINC02544	3.922124051	8.62366801	4.10E-93	2.31E-91
CAVIN2-AS1	-3.331179779	4.058341601	8.10E-93	4.49E-91
TMEM220-AS1	-1.749450916	6.628280299	1.44E-90	7.86E-89
MIR22HG	-1.375404687	11.07048109	2.33E-90	1.24E-88
SACS-AS1	-2.944357134	3.817858317	2.79E-90	1.47E-88
LINC01942	-3.242175098	2.91115156	6.84E-90	3.54E-88
LINC02104	-2.331245937	4.816748341	1.22E-89	6.19E-88
LINC00924	-2.135553704	6.514083544	1.79E-88	8.99E-87
LINC01586	-3.146452223	3.294983075	1.83E-88	9.03E-87
LINC01561	4.929540096	6.020413655	9.91E-88	4.81E-86
C10orf71-AS1	-7.003768436	3.53136806	4.60E-87	2.20E-85
LCT-AS1	4.189665603	5.135662803	5.81E-87	2.74E-85
EMX2OS	-2.143420053	8.801762597	2.46E-85	1.14E-83
LINC00989	-1.976195025	5.202442242	3.60E-85	1.65E-83
FGF13-AS1	-2.676508926	5.210618587	4.22E-84	1.90E-82
LMO7DN-IT1	-2.643175467	3.034580454	1.25E-82	5.55E-81
PRDM16-DT	-2.154990347	6.172560424	5.76E-81	2.52E-79
LINC01197	-1.755234449	7.290641204	1.04E-80	4.50E-79
LINC01474	-2.709209618	3.433712785	3.68E-80	1.57E-78
ZEB2-AS1	-1.690630264	5.017822129	6.61E-80	2.78E-78
HORMAD2-AS1	-2.479230018	4.227465981	3.47E-79	1.44E-77
PGM5-AS1	-3.233798628	4.942059715	6.62E-79	2.71E-77
LINC01091	-1.864716607	6.135293909	4.88E-77	1.97E-75
B4GALT1-AS1	-1.436707052	7.340976998	5.77E-77	2.31E-75
TMPO-AS1	2.078418733	8.856527855	7.30E-77	2.88E-75
PCAT19	-1.473505561	9.644505825	3.89E-76	1.52E-74
FGF14-AS2	-1.822846788	7.941720131	2.45E-75	9.45E-74
LINC01985	-2.854004254	3.92157406	7.87E-75	2.99E-73
LINC02289	-1.897948312	5.942090756	1.35E-74	5.08E-73
RHPN1-AS1	2.134372355	9.600069104	1.06E-73	3.93E-72
LINC02408	3.342254218	5.063117944	4.24E-73	1.56E-71
KCNJ2-AS1	-1.839061688	6.545350034	1.26E-72	4.55E-71
TPT1-AS1	-1.277965383	10.64568529	1.74E-72	6.26E-71
MIR99AHG	-1.725464485	9.189440472	3.32E-72	1.18E-70
LINC01589	-2.179650477	4.753795487	8.67E-72	3.04E-70
MME-AS1	-3.096494322	3.066394722	6.11E-71	2.12E-69
MAFG-DT	2.04639801	10.27529867	2.47E-70	8.49E-69
LINC02227	-2.589511166	3.615131509	5.48E-70	1.86E-68
LINC00671	-2.345231747	4.056225088	6.05E-68	2.03E-66
LINC01366	-1.84981175	4.109658322	6.51E-68	2.16E-66
CHL1-AS2	-2.703989887	4.413238977	8.81E-68	2.90E-66
GNG12-AS1	-1.561774682	6.834269589	1.31E-67	4.25E-66
LINC00640	-2.154476491	5.211889857	1.79E-67	5.76E-66
RNU6ATAC35P	-2.30556448	4.029927861	5.01E-67	1.60E-65
HAND2-AS1	-1.679064643	7.412281186	6.04E-66	1.91E-64

TDRKH-AS1	1.616043908	7.220470627	6.44E-66	2.01E-64
LINC01625	-2.740958374	4.602187739	2.49E-65	7.70E-64
MAFTRR	-1.806033545	5.617086266	4.86E-65	1.49E-63
LINC00445	-3.039447567	3.865453263	6.04E-65	1.83E-63
LINC00337	3.069099717	5.969098554	9.43E-65	2.83E-63
NGF-AS1	-3.586956538	5.071115102	2.11E-64	6.27E-63
LINC00641	-1.242762096	10.27456237	5.32E-64	1.57E-62
BSG-AS1	2.835807342	8.297086681	5.60E-64	1.64E-62
LGALS8-AS1	2.66537281	6.5160119	7.36E-64	2.13E-62
C9orf163	1.874291598	6.719390747	2.97E-63	8.52E-62
ELF3-AS1	1.65128503	7.872628324	5.69E-63	1.62E-61
MBNL1-AS1	-1.551762878	9.248096193	8.49E-63	2.39E-61
LINC02580	-1.96234861	5.258895222	9.51E-63	2.66E-61
CADM3-AS1	-2.445365844	7.281725477	8.43E-62	2.33E-60
LINC02066	-3.163757713	2.896937471	8.55E-62	2.35E-60
PDZRN3-AS1	-2.803192114	3.112618037	1.56E-61	4.26E-60
LINC02391	-1.624740251	5.161027877	1.88E-61	5.07E-60
LINC00466	4.290975809	5.162577638	5.86E-61	1.57E-59
MIR100HG	-1.387735027	11.03917987	9.89E-60	2.62E-58
POLH-AS1	1.701907855	6.783256205	1.22E-59	3.22E-58
PTENP1-AS	-2.536369814	3.449010176	6.67E-59	1.74E-57
ZBED3-AS1	-1.160716927	7.650933878	9.09E-59	2.35E-57
A2M-AS1	-1.138531764	7.736365933	1.30E-58	3.34E-57
CDKN2B-AS1	2.295466941	7.079481244	2.82E-58	7.17E-57
ATP2A1-AS1	2.418094486	6.905652104	3.51E-58	8.87E-57
SCTR-AS1	-2.681926176	4.239575895	4.58E-58	1.15E-56
WDR86-AS1	-1.992695199	7.323226326	9.29E-58	2.31E-56
LINC01943	2.105170577	6.595356989	9.47E-58	2.34E-56
LINC02881	-1.968034656	4.128819376	2.73E-57	6.69E-56
LINC00645	-3.975645152	3.912631085	4.29E-57	1.04E-55
WDFY3-AS2	-1.195627056	8.76407799	5.06E-57	1.22E-55
LINC01886	-3.364380419	2.940614969	5.36E-57	1.28E-55
LINC02766	-2.14798855	3.428040928	5.57E-57	1.32E-55
TYMSOS	2.449421927	7.51691209	5.63E-57	1.33E-55
MIR23AHG	-1.460117574	10.96620665	6.70E-57	1.57E-55
SNHG26	-1.885228285	7.526171617	8.79E-57	2.04E-55
CD2BP2-DT	1.959227314	9.517525361	1.23E-56	2.83E-55
DDX11-AS1	1.754099455	7.233690091	2.17E-56	4.97E-55
LRRC8C-DT	-1.391667082	6.591527709	4.10E-56	9.30E-55
FOXD3-AS1	4.692781669	8.499766282	5.56E-56	1.25E-54
LINC01186	-2.835665823	4.289560399	1.05E-55	2.34E-54
LINC01594	-3.166643082	3.284407572	1.18E-55	2.62E-54
LINC01352	-1.594167963	5.600387016	1.91E-55	4.22E-54
RAPGEF4-AS1	-2.35347306	3.398791694	2.02E-55	4.42E-54
AGAP1-IT1	2.760208581	6.432365518	2.88E-55	6.27E-54
GSEC	1.604101618	9.055820032	4.00E-55	8.64E-54
JAZF1-AS1	-1.508146423	4.179748685	7.17E-55	1.54E-53
LINC01058	-1.607804789	4.591341891	9.99E-55	2.13E-53
STK32A-AS1	-2.435038992	3.331504397	1.21E-54	2.57E-53
PCAT6	1.904675319	9.774792564	2.03E-54	4.28E-53
LINC02894	1.943683871	8.851691358	2.93E-54	6.12E-53

LINC00906	-2.73678765	3.007126867	5.21E-54	1.08E-52
MEG3	-1.568905687	11.61454296	6.38E-54	1.32E-52
LINC01028	-3.56333003	4.467456235	6.50E-54	1.33E-52
LINC01402	-1.834590094	4.037901282	1.44E-53	2.92E-52
IL6-AS1	-2.223633702	5.144755622	4.13E-53	8.35E-52
NDUFB2-AS1	1.093995831	7.23548409	1.26E-52	2.53E-51
LINC02172	-1.977109592	3.576419342	3.01E-52	6.02E-51
LINC00665	1.397998662	12.5454782	3.19E-52	6.34E-51
CAPN10-DT	1.097776575	8.297294093	4.84E-52	9.55E-51
LINC01929	3.75001534	8.201128947	5.69E-52	1.12E-50
ZNF687-AS1	1.338450585	6.349575642	6.50E-52	1.27E-50
SOCAR	2.426615773	4.968514374	1.52E-51	2.94E-50
LAMA4-AS1	-1.526528021	4.698568653	3.86E-51	7.43E-50
LINC01405	-4.746862739	5.435600765	6.16E-51	1.18E-49
CYP2U1-AS1	-2.040631311	3.961838791	8.62E-51	1.64E-49
LAMC1-AS1	-1.60761694	4.989080387	8.89E-51	1.68E-49
VLDLR-AS1	-1.880057479	7.138923446	1.12E-50	2.10E-49
CNTFR-AS1	-2.895234175	3.891245524	3.93E-50	7.34E-49
ST3GAL1-DT	2.077946177	7.376387839	9.02E-50	1.67E-48
DCST1-AS1	1.564481919	8.671084144	1.44E-49	2.65E-48
LINC01569	1.387544387	9.246648168	1.60E-49	2.94E-48
LINC01429	3.570052227	4.418860326	2.88E-49	5.26E-48
LASTR	2.664037179	5.174028202	4.34E-49	7.87E-48
LINC00598	-1.305433482	4.569510302	5.40E-49	9.75E-48
TFAP2A-AS1	1.464350097	7.689162296	6.40E-49	1.15E-47
LINC02817	-1.963113363	3.611683467	1.29E-48	2.31E-47
TONSL-AS1	1.732103012	5.687222516	3.94E-48	7.00E-47
DLEU2	1.381940525	9.240681348	7.09E-48	1.25E-46
MIR4435-2HG	1.263989826	11.64661985	8.45E-48	1.48E-46
PRICKLE2-DT	3.771711434	3.561752343	1.59E-47	2.77E-46
KLHDC7B-DT	3.277138718	8.193424207	1.64E-47	2.84E-46
LNCOG	-1.573085973	5.850196326	2.20E-47	3.79E-46
KLF3-AS1	-1.234601256	7.934435663	5.98E-47	1.03E-45
C5orf66-AS1	6.030357651	6.807843005	1.74E-46	2.96E-45
PVT1	1.741799334	11.47984134	3.33E-46	5.61E-45
SOCS3-DT	2.294816176	6.961032826	4.88E-46	8.20E-45
TTLL10-AS1	-1.779224996	4.23517363	6.53E-46	1.09E-44
C5orf66	1.329132378	7.701339981	6.84E-46	1.14E-44
LINC00511	3.082407899	9.728535524	1.14E-45	1.89E-44
LINC00467	1.145098895	10.90391721	1.93E-45	3.17E-44
SYNE1-AS1	-2.040487606	2.982769011	3.43E-45	5.62E-44
SMCR2	2.906958355	5.010395885	6.79E-45	1.10E-43
LINC01340	-2.18564676	3.14763836	7.51E-45	1.22E-43
NRAV	1.079491096	11.39007409	1.70E-44	2.74E-43
SENCR	-1.231066395	6.598069742	2.63E-44	4.21E-43
LINC02086	3.819418977	5.916845788	3.57E-44	5.69E-43
KCNIP1-OT1	-2.22410892	3.385974446	3.98E-44	6.32E-43
FAM87B	-1.111521511	5.119974998	4.58E-44	7.23E-43
LINC01094	1.442843503	9.20251935	4.73E-44	7.44E-43
SRRM2-AS1	1.409610847	8.498104491	5.52E-44	8.63E-43
LINC02458	-1.850778252	4.283686086	9.20E-44	1.43E-42

BOK-AS1	-2.463767157	4.59493641	1.74E-43	2.69E-42
LINC01412	-2.011093919	2.995772886	1.78E-43	2.75E-42
MIR3150BHG	2.802784177	6.298433811	2.90E-43	4.44E-42
LINC01775	2.432297242	4.360633246	3.46E-43	5.28E-42
LINC01344	3.803028994	6.273773888	4.72E-43	7.16E-42
TBX18-AS1	-1.792810133	3.757737113	4.77E-43	7.20E-42
RNF139-AS1	1.486924448	9.063708068	1.16E-42	1.75E-41
LIPE-AS1	-1.145056296	8.350696171	3.52E-42	5.26E-41
COL4A2-AS1	-1.613326162	3.868614077	8.77E-42	1.31E-40
RXYLT1-AS1	-1.517145407	3.675683003	2.94E-41	4.35E-40
DHCR24-DT	1.623680819	5.628003414	3.52E-41	5.20E-40
LINC01854	-4.875112531	3.943433804	3.67E-41	5.39E-40
SAMD12-AS1	1.889409822	6.132712493	3.80E-41	5.55E-40
HOXC-AS3	3.732002427	7.569974529	4.72E-41	6.87E-40
LINC00636	-2.21208914	3.463020123	4.84E-41	7.02E-40
PAPOLA-DT	1.349642616	6.8774731	5.22E-41	7.53E-40
LINC01612	-3.069833518	3.950677168	5.81E-41	8.33E-40
SLC12A5-AS1	3.058545625	5.631581959	6.37E-41	9.10E-40
FBXL19-AS1	1.535671858	9.616439941	1.11E-40	1.58E-39
PPP1R26-AS1	1.261169221	9.133026407	1.34E-40	1.89E-39
CYTOR	1.312635513	11.12349792	1.56E-40	2.19E-39
ABCA9-AS1	-2.102515731	3.33554735	1.58E-40	2.21E-39
HOXA-AS2	-1.30156772	8.303719009	2.37E-40	3.31E-39
SNHG25	2.117821429	6.474677152	2.60E-40	3.62E-39
MAP3K4-AS1	1.602901935	9.743906888	2.71E-40	3.75E-39
CERS3-AS1	-2.356654914	3.941272384	3.23E-40	4.45E-39
ADCY6-DT	1.890767751	5.493552089	3.57E-40	4.90E-39
LINC00506	-1.249486918	5.779539464	1.02E-39	1.38E-38
SCAT2	1.446049362	5.973237633	1.48E-39	2.01E-38
LINC01415	-1.359149914	5.828827604	1.55E-39	2.08E-38
PPP1R14B-AS1	1.994285257	7.462475921	2.26E-39	3.02E-38
HOTAIRM1	-1.307242922	8.639173105	3.43E-39	4.55E-38
NKILA	1.532794357	9.345011262	4.05E-39	5.35E-38
LYPLAL1-AS1	-1.79079041	6.048163284	4.37E-39	5.75E-38
SDK1-AS1	-1.91675486	3.760390304	5.31E-39	6.96E-38
MIR1-1HG-AS1	-2.343637202	3.727917761	6.77E-39	8.83E-38
LINC02321	2.529709181	5.352476674	1.28E-38	1.67E-37
LINC00484	-1.865245153	4.487001225	1.33E-38	1.72E-37
LINC02607	-2.472012553	8.13136266	1.55E-38	2.00E-37
MIR210HG	1.806234746	8.947885463	1.87E-38	2.40E-37
LINC01615	2.104796073	6.789537757	3.96E-38	5.06E-37
WT1-AS	4.855457319	7.079824157	4.29E-38	5.46E-37
RUNDC3A-AS1	2.542323552	7.673135999	5.03E-38	6.38E-37
UNQ6494	2.027561822	5.482219972	5.46E-38	6.90E-37
COPDA1	4.221472582	6.768860695	8.81E-38	1.11E-36
LINC01711	1.77302165	6.935858591	8.99E-38	1.13E-36
LMF1-AS1	-1.392432768	4.837820359	1.80E-37	2.23E-36
CLCA4-AS1	-1.939946089	3.133307146	1.86E-37	2.31E-36
LINC00702	-1.373491021	8.740947412	2.19E-37	2.70E-36
HAGLROS	2.548820969	7.145264041	2.51E-37	3.08E-36
SUV39H2-DT	1.287409328	7.098795559	3.16E-37	3.86E-36

LINC01354	-1.74973721	6.976469603	3.35E-37	4.08E-36
LINC02599	-1.463885329	4.378063885	5.44E-37	6.59E-36
LINC02256	-1.08811205	5.63432679	5.84E-37	7.04E-36
ZNF252P-AS1	1.35630065	6.107820886	6.92E-37	8.31E-36
LINC01522	5.500595978	6.978202206	7.10E-37	8.50E-36
LINC01908	-2.173578027	3.351404213	7.57E-37	9.01E-36
LINC01894	-1.837440478	4.44711285	9.72E-37	1.15E-35
PPP2CA-DT	1.363244765	7.301536657	9.96E-37	1.17E-35
LINC01359	-1.352794711	6.341508371	1.07E-36	1.25E-35
POU2F1-DT	1.702363456	5.108858494	1.64E-36	1.91E-35
LINC00460	2.928923102	5.831795826	2.26E-36	2.62E-35
TMEM14B-DT	1.077093782	6.3908228	2.77E-36	3.20E-35
C1orf220	1.440648634	7.353950074	4.47E-36	5.15E-35
CHL1-AS1	-2.267607937	3.160555647	7.66E-36	8.76E-35
LINC01588	1.803019915	9.197439363	1.01E-35	1.16E-34
SLC14A2-AS1	-1.96307906	4.425153702	1.67E-35	1.88E-34
HOXA-AS3	-1.670628564	6.192216079	1.72E-35	1.93E-34
CCND2-AS1	-1.565795126	3.331854223	1.96E-35	2.20E-34
IER3-AS1	1.614427109	5.6932584	2.30E-35	2.57E-34
LINC01136	2.050830257	5.68740975	2.46E-35	2.75E-34
TENM3-AS1	-1.821417252	7.032221968	3.00E-35	3.33E-34
LINC01088	-1.797526922	6.882634918	5.04E-35	5.58E-34
LINC01117	2.228891746	7.223348022	5.33E-35	5.88E-34
GASAL1	1.294839403	7.622656667	6.70E-35	7.37E-34
CCDC183-AS1	1.335776126	8.994306071	9.33E-35	1.02E-33
LINC02541	-1.541843764	5.370586529	1.40E-34	1.53E-33
PYCARD-AS1	1.943594432	4.616010028	1.41E-34	1.53E-33
LINC01281	3.581624788	5.012831678	1.56E-34	1.69E-33
CSGALNACT2-DT	1.028962205	6.24824483	1.78E-34	1.92E-33
ZKSCAN2-DT	1.007058472	8.61701056	2.24E-34	2.41E-33
CHKB-DT	1.284149049	6.912426957	2.34E-34	2.51E-33
MRPL20-DT	1.220295939	8.658103567	2.71E-34	2.90E-33
C15orf54	3.206770246	4.973407981	2.86E-34	3.05E-33
LINC01484	-1.935426526	5.463930385	3.90E-34	4.14E-33
ARHGAP29-AS1	3.430810236	6.67940317	4.77E-34	5.05E-33
FAM83C-AS1	2.472392717	3.598620978	6.96E-34	7.35E-33
LINC00536	2.891336495	7.079045782	1.32E-33	1.39E-32
GHET1	1.274905591	6.794321778	1.44E-33	1.52E-32
SMAD9-IT1	-1.517957303	3.334516211	1.67E-33	1.75E-32
MIR200CHG	1.539333025	9.077164221	1.79E-33	1.87E-32
TLX1NB	5.638105346	4.788130507	1.84E-33	1.91E-32
LINC02716	-1.701357753	5.132345622	1.85E-33	1.92E-32
STAM-AS1	1.301631057	5.81164031	2.21E-33	2.28E-32
EIPR1-IT1	2.155076642	5.110454844	2.50E-33	2.55E-32
LINC01267	-1.723452926	3.663316228	3.48E-33	3.54E-32
LINC01978	2.353940661	5.203554345	3.97E-33	4.03E-32
MIAT	1.823952596	10.07435242	4.13E-33	4.18E-32
KCNH1-IT1	4.149193537	5.399489652	4.99E-33	5.04E-32
LINC00115	1.046457534	6.571969471	1.21E-32	1.22E-31
LINC02685	3.06346206	3.563850332	1.77E-32	1.77E-31
EMSLR	1.883717921	8.375048821	2.01E-32	2.01E-31

LINC00940	2.682327587	7.633755845	2.48E-32	2.47E-31
DGUOK-AS1	1.160976373	7.837489469	2.90E-32	2.88E-31
FLJ12825	2.30744912	7.753508117	3.81E-32	3.77E-31
LINC01224	3.984453188	7.653133375	3.89E-32	3.83E-31
LINC01532	-2.342711472	3.14419869	3.90E-32	3.84E-31
LINC02489	3.764320572	5.515077505	4.39E-32	4.31E-31
CIRBP-AS1	1.049235279	7.375163502	5.00E-32	4.89E-31
LINC01977	2.162923207	8.997220636	5.19E-32	5.05E-31
GEMIN7-AS1	1.05309337	6.905433595	5.51E-32	5.36E-31
LINC00243	2.167832285	6.813350972	5.83E-32	5.63E-31
LINC01842	3.38676815	5.939403952	6.40E-32	6.15E-31
ARHGEF2-AS2	1.268572674	6.927489908	6.41E-32	6.15E-31
LINC02352	-1.229276439	6.080330673	6.55E-32	6.27E-31
LBX2-AS1	1.19467032	10.36475093	7.44E-32	7.09E-31
SFTA1P	-1.487018021	5.87550098	8.69E-32	8.27E-31
LINC02139	-1.309243637	3.984189461	9.48E-32	8.99E-31
HLX-AS1	-1.435508773	3.99298142	9.90E-32	9.36E-31
LYPLAL1-DT	-1.203331847	4.901038856	1.10E-31	1.03E-30
LINC02388	-1.929070546	3.282653145	1.66E-31	1.56E-30
CRIM1-DT	-1.125762274	7.800036694	1.69E-31	1.58E-30
MAFA-AS1	3.77747678	4.448479645	2.31E-31	2.16E-30
CH17-340M24.3	1.077483736	9.722007808	2.33E-31	2.17E-30
LINC02878	1.299120586	6.857062566	2.89E-31	2.68E-30
CERS6-AS1	1.864654896	4.446399119	3.58E-31	3.32E-30
SNHG3	1.211564325	11.67373282	6.21E-31	5.73E-30
PLA2G4C-AS1	2.170697221	4.897905129	6.83E-31	6.29E-30
GATA3-AS1	2.816319974	10.50339763	1.01E-30	9.27E-30
DLG3-AS1	2.023457043	5.168393904	1.08E-30	9.84E-30
RCCD1-AS1	1.125690719	5.166182385	1.17E-30	1.07E-29
LINC02891	-1.369992698	3.530324729	1.30E-30	1.18E-29
FZD4-DT	-1.074057451	6.597226883	1.48E-30	1.35E-29
LINC01152	-2.131804583	8.69396535	1.80E-30	1.63E-29
LINC01238	3.527293562	11.39845276	1.84E-30	1.66E-29
APCDD1L-DT	-1.961707288	8.502169434	1.88E-30	1.69E-29
HOTAIR	3.075667597	10.0733425	1.92E-30	1.72E-29
FLG-AS1	-1.238948815	6.532488372	2.39E-30	2.13E-29
LINC02613	-1.887889331	9.025439043	2.44E-30	2.17E-29
HPN-AS1	1.699783015	5.330765605	2.66E-30	2.36E-29
SLC9A3R1-AS1	2.033517863	9.202028999	3.47E-30	3.07E-29
CLRN1-AS1	3.062802064	4.957674913	4.18E-30	3.67E-29
LINC01703	1.393765905	7.342856294	4.34E-30	3.79E-29
MEG8	-1.424235652	4.014895686	6.46E-30	5.64E-29
LINC02804	1.91472169	4.946759067	7.36E-30	6.41E-29
LINC02528	3.233504388	4.377571725	8.01E-30	6.93E-29
TP53RK-DT	1.403985478	5.742414474	8.38E-30	7.23E-29
LINC01747	1.865011372	3.704045563	8.54E-30	7.35E-29
NARF-AS1	1.684997906	4.133684876	9.24E-30	7.93E-29
LINC01624	-1.249441809	4.14928972	9.53E-30	8.15E-29
THOC1-DT	1.197660129	5.346060724	9.77E-30	8.33E-29
BVES-AS1	-1.666629638	4.656885951	1.01E-29	8.61E-29
TP73-AS3	3.148633325	3.648812276	1.02E-29	8.68E-29

GORAB-AS1	1.606440882	6.908492068	1.54E-29	1.30E-28
OTULIN-DT	1.167592679	6.066641789	1.66E-29	1.40E-28
LINC02166	1.090000216	5.903581286	1.88E-29	1.58E-28
TEX26-AS1	-1.737033184	4.664439795	2.10E-29	1.75E-28
LINC01063	1.644759011	5.040055758	2.17E-29	1.80E-28
MNX1-AS1	3.189741332	7.72982881	2.27E-29	1.88E-28
LINC01497	-2.783285058	4.429276849	2.65E-29	2.19E-28
BRWD1-AS2	1.14824554	6.810584882	3.62E-29	2.99E-28
LINC02762	-1.148806987	8.084894841	4.99E-29	4.10E-28
LMNTD2-AS1	2.003926633	9.344642558	5.53E-29	4.54E-28
TPRG1-AS1	-1.344481657	6.638860139	8.11E-29	6.63E-28
UST-AS2	1.917472054	3.802984023	1.19E-28	9.68E-28
CBR3-AS1	1.138568323	9.765914532	1.42E-28	1.15E-27
WASIR2	2.115952569	7.1340079	1.93E-28	1.56E-27
LINC00853	1.300501055	7.060831258	2.74E-28	2.19E-27
UNC5B-AS1	1.954352459	5.780744369	3.22E-28	2.57E-27
LINC00607	1.873019116	7.619818097	3.36E-28	2.68E-27
MYHAS	-1.340098408	5.230850219	3.52E-28	2.80E-27
FUT8-AS1	1.909516686	7.149315379	5.40E-28	4.27E-27
MIR193BHG	-1.045180123	7.179250009	5.92E-28	4.67E-27
LINC00920	-1.156623716	7.668761234	7.32E-28	5.77E-27
DEPDC1-AS1	2.744228332	3.733720223	7.77E-28	6.10E-27
GAPLINC	1.137789534	6.803890011	8.11E-28	6.36E-27
TSBP1-AS1	-1.406378273	3.418117287	8.32E-28	6.50E-27
LINC02798	-1.283609453	6.158170478	8.35E-28	6.51E-27
MGAT3-AS1	-1.872735417	3.569728477	8.99E-28	6.99E-27
ZNF132-DT	1.284990605	6.67694021	1.08E-27	8.36E-27
LINC02739	-1.215413247	3.61032888	1.25E-27	9.67E-27
KLHL30-AS1	-2.166044673	3.466909892	1.68E-27	1.30E-26
ST8SIA6-AS1	3.294276678	9.791047107	1.92E-27	1.47E-26
MRPS30-DT	3.340667075	12.0032183	2.12E-27	1.63E-26
MEG9	-1.324906257	4.954819708	2.56E-27	1.96E-26
LINC02593	2.509486146	9.61586175	2.95E-27	2.24E-26
LINC00884	1.401999906	8.261987593	3.43E-27	2.60E-26
RALGPS2-AS1	1.600409775	6.440078117	3.47E-27	2.62E-26
HOXB-AS1	-1.234585518	8.958405076	3.88E-27	2.92E-26
LINC02676	4.072303049	3.513188153	3.90E-27	2.93E-26
TBILA	1.762852603	8.404005597	3.98E-27	2.99E-26
LINC02012	1.507557656	6.399364397	4.33E-27	3.24E-26
SMYD3-AS1	3.598745676	4.801258932	4.99E-27	3.73E-26
UBE2Q1-AS1	1.301159346	4.637994385	5.21E-27	3.88E-26
KRTAP5-AS1	1.933209284	8.131795198	5.25E-27	3.90E-26
LINC01505	3.013339851	5.063987188	6.02E-27	4.46E-26
LINC02747	3.895875141	9.534563232	7.09E-27	5.24E-26
LINC02847	-1.528539122	3.083903754	7.29E-27	5.38E-26
LINC00211	-1.339462216	3.576884436	7.48E-27	5.51E-26
ATXN7L3-AS1	1.114986674	5.244772011	7.59E-27	5.57E-26
HDAC11-AS1	1.822103459	3.690704654	9.22E-27	6.73E-26
LINC02712	-1.667154987	4.749518559	1.17E-26	8.52E-26
C9orf106	1.83279973	6.811366066	1.19E-26	8.63E-26
DNAH10OS	1.370882527	9.431468352	1.55E-26	1.12E-25

LINC02771	2.559213444	4.316010507	1.55E-26	1.12E-25
LINC02195	2.619819502	4.078145118	1.61E-26	1.15E-25
JARID2-DT	1.334297436	6.37894499	2.25E-26	1.62E-25
DSCAM-AS1	5.964686411	11.09888458	2.65E-26	1.90E-25
FRG1-DT	-1.187308497	6.278568277	3.22E-26	2.29E-25
CASC16	4.161403794	6.836024323	4.58E-26	3.26E-25
PCAT7	2.075378188	6.899473224	5.88E-26	4.16E-25
PROX1-AS1	-1.830028889	4.512548968	6.58E-26	4.65E-25
SH3TC2-DT	-1.700345613	3.147444113	7.39E-26	5.21E-25
LINC01644	3.954952229	7.274046438	7.42E-26	5.22E-25
RFX5-AS1	1.177427679	6.1688381	7.49E-26	5.26E-25
ELFN1-AS1	4.590711193	7.053310378	8.72E-26	6.09E-25
GRASLND	1.438433909	7.861402798	9.12E-26	6.35E-25
DIO3OS	-1.499913424	8.058497701	9.60E-26	6.68E-25
AFAP1-AS1	3.935848242	9.711631097	1.16E-25	8.03E-25
THBS3-AS1	1.17262699	8.138203403	1.64E-25	1.13E-24
DNAAF3-AS1	2.465200646	3.468124935	1.70E-25	1.16E-24
DPY19L3-DT	1.188152412	6.659289872	1.75E-25	1.20E-24
LINC02870	2.368259125	5.586420813	2.38E-25	1.62E-24
BMPR1B-DT	4.54029487	7.418796795	2.77E-25	1.88E-24
FIGNL2-DT	-1.460599188	3.512880481	4.05E-25	2.75E-24
LINC00670	-1.869454892	2.989706871	5.07E-25	3.42E-24
SCAT1	1.810081875	4.269558642	5.24E-25	3.52E-24
DPYD-AS1	-1.680742241	3.21031872	5.51E-25	3.70E-24
LINC01116	1.567344671	10.81460574	5.79E-25	3.88E-24
THBS1-IT1	1.832766771	8.329000767	5.92E-25	3.96E-24
EHD4-AS1	-1.160224635	4.413574155	6.28E-25	4.19E-24
LINC01096	2.60788595	5.164767604	7.71E-25	5.13E-24
HS1BP3-IT1	-1.118375904	4.107054955	1.00E-24	6.68E-24
LINC02623	-1.839327931	3.218124049	1.04E-24	6.87E-24
PHEX-AS1	-2.318139502	3.462986541	1.20E-24	7.91E-24
MAL2-AS1	1.622247599	5.085383696	1.77E-24	1.16E-23
FALEC	1.282068634	5.143034453	2.18E-24	1.43E-23
LINC01213	2.155195813	5.542890417	2.37E-24	1.55E-23
SPACA6P-AS	1.427003697	5.552907672	2.58E-24	1.68E-23
SNHG9	1.224967553	9.502067809	3.69E-24	2.39E-23
YTHDF3-DT	1.225051083	7.511081029	3.95E-24	2.55E-23
ST6GAL2-IT1	2.14593958	3.594603395	4.23E-24	2.72E-23
SEPTIN9-DT	2.055743135	4.35100689	4.62E-24	2.97E-23
POT1-AS1	1.118139968	7.254525059	4.74E-24	3.04E-23
LINC01050	2.633445446	4.230765091	4.83E-24	3.09E-23
DYRK3-AS1	1.482790642	4.562057852	5.68E-24	3.63E-23
THBS1-AS1	2.640650737	9.892111424	5.92E-24	3.78E-23
LINC01264	2.488546959	3.696378427	6.82E-24	4.33E-23
MMP2-AS1	1.803940669	6.545602458	7.41E-24	4.70E-23
USP30-AS1	1.596175614	6.610879989	8.01E-24	5.07E-23
MIR924HG	1.663835755	7.780473812	8.58E-24	5.42E-23
RNF157-AS1	1.480059658	6.49018876	9.49E-24	5.98E-23
DDX3ILA1	3.367366821	4.936688423	1.34E-23	8.42E-23
LINC02888	4.501909638	6.652596426	1.42E-23	8.89E-23
LINC02806	2.448499118	4.436774823	1.59E-23	9.91E-23

SIAH2-AS1	1.641290476	6.281068327	1.83E-23	1.13E-22
LINC02280	2.146340681	5.222341652	1.85E-23	1.15E-22
LINC01649	2.809693374	4.36214246	1.86E-23	1.15E-22
FOXD2-AS1	1.104452879	8.641436938	1.92E-23	1.18E-22
LINC01857	1.834626476	6.273082577	1.93E-23	1.19E-22
LINC02288	-1.023567361	4.903905197	2.45E-23	1.50E-22
LINC02620	3.481808525	7.419624744	2.58E-23	1.58E-22
LINC00840	-1.186779117	5.886961349	2.64E-23	1.62E-22
HEATR6-DT	1.164673134	5.514150963	3.18E-23	1.93E-22
ABALON	1.084685475	6.381813531	3.38E-23	2.05E-22
TIMMDC1-DT	1.632183486	4.014020063	3.93E-23	2.38E-22
DNAH17-AS1	3.02329014	4.907200494	5.42E-23	3.27E-22
LINC01422	-1.026484212	5.779188825	6.36E-23	3.82E-22
LINC02236	1.928125373	3.779713586	6.55E-23	3.93E-22
SIRLNT	7.176124228	9.781659153	8.98E-23	5.38E-22
LINC02006	-1.577443021	4.535924861	9.82E-23	5.87E-22
LINC02416	2.544628605	3.880759783	1.07E-22	6.41E-22
LINC02846	1.358794188	5.631785568	1.68E-22	1.00E-21
EHMT2-AS1	1.593465466	4.11096244	1.93E-22	1.14E-21
C1orf21-DT	1.302849735	6.051669582	2.11E-22	1.25E-21
LY86-AS1	-1.300699802	4.485689036	2.36E-22	1.40E-21
LINC01426	1.439219997	7.91254525	2.50E-22	1.47E-21
ZNF350-AS1	2.408422754	7.654511378	2.56E-22	1.51E-21
LINC00578	2.555453083	8.042351544	2.81E-22	1.65E-21
BCAR4	4.844662413	6.002046745	2.90E-22	1.70E-21
MIR155HG	1.389700308	7.629918627	3.65E-22	2.14E-21
SSTR5-AS1	5.586351279	5.706310931	3.99E-22	2.33E-21
CYP4A22-AS1	1.149023925	5.396695663	4.18E-22	2.44E-21
LINC02201	2.535829822	3.684137037	4.49E-22	2.61E-21
TWSG1-DT	1.352232089	5.255141756	5.67E-22	3.29E-21
DSCR8	5.53161826	6.021543164	6.52E-22	3.78E-21
LINC00160	1.945022371	7.380366688	8.58E-22	4.96E-21
CD2AP-DT	1.147016378	6.437544701	8.94E-22	5.16E-21
LINC02257	1.977786484	5.344127865	1.24E-21	7.12E-21
ARHGEF26-AS1	1.527608089	8.431724934	1.24E-21	7.12E-21
B3GAT1-DT	-2.049276541	6.563324944	1.53E-21	8.74E-21
SRGAP3-AS2	4.632537465	5.37802278	1.63E-21	9.30E-21
LINC00870	1.836552791	4.557886701	1.64E-21	9.37E-21
DSCR9	1.253983261	4.806786286	2.17E-21	1.24E-20
LINC02889	2.695576743	5.838909923	2.76E-21	1.57E-20
LINC02224	4.608123712	9.019725553	3.38E-21	1.90E-20
TCL6	2.808021216	6.086725985	3.50E-21	1.97E-20
PCOTH	1.441665518	6.377475783	3.57E-21	2.00E-20
STK4-AS1	1.133911814	6.310863081	4.09E-21	2.29E-20
LINC00659	3.702527357	4.140873418	4.77E-21	2.66E-20
LINC00628	1.861875381	4.239287847	5.13E-21	2.85E-20
FNDC1-IT1	2.343793112	4.120663196	5.18E-21	2.87E-20
LINC02026	1.32056479	4.930257245	5.26E-21	2.92E-20
UBE2E2-AS1	-1.462286639	2.997904393	7.05E-21	3.89E-20
LINC01572	1.115237712	5.558546004	7.13E-21	3.93E-20
DNAH8-AS1	2.638874848	3.062873294	7.37E-21	4.05E-20

LINC02867	1.693625943	4.600938923	8.23E-21	4.51E-20
LINC01605	1.814069034	7.567876761	8.50E-21	4.65E-20
DHX35-DT	1.7350044	4.027924376	8.69E-21	4.75E-20
LINC01814	1.45585473	7.565565644	8.76E-21	4.78E-20
LINC00052	6.211671922	9.845109892	9.55E-21	5.20E-20
LINC02827	1.349862473	5.905435012	1.07E-20	5.83E-20
LINC01341	1.349062626	7.86188604	1.24E-20	6.73E-20
TMEM75	2.185290458	4.988078829	1.28E-20	6.90E-20
CBARP-DT	1.564626608	4.182624057	1.30E-20	7.02E-20
LINC02532	2.86768094	6.867014875	1.44E-20	7.76E-20
CNIH3-AS2	1.450325633	4.555256386	1.70E-20	9.14E-20
LINC00887	1.622845667	6.365036966	2.30E-20	1.23E-19
DLX2-DT	3.03516102	4.263944202	2.62E-20	1.40E-19
LINC00605	1.985477703	5.527260561	2.93E-20	1.56E-19
RIPOR3-AS1	2.976783444	4.672701658	3.28E-20	1.75E-19
LINC01060	-1.159427563	5.925797626	3.37E-20	1.79E-19
LINC00664	1.468854559	7.057343518	4.49E-20	2.38E-19
MELTF-AS1	1.071742326	8.746118202	4.65E-20	2.46E-19
LINC01844	-1.452663088	4.314848823	5.53E-20	2.93E-19
LINC02263	3.032004423	2.986539424	5.86E-20	3.09E-19
CFAP58-DT	1.753050624	6.461248441	6.16E-20	3.24E-19
RASGRF2-AS1	-1.128791317	4.331223453	6.44E-20	3.38E-19
SPON1-AS1	-1.295209946	3.139470558	1.12E-19	5.81E-19
LINC02461	2.148848126	4.56808421	1.84E-19	9.50E-19
SYNPR-AS1	2.267085636	5.762377307	1.88E-19	9.70E-19
LINC01909	1.126692706	6.462142648	1.91E-19	9.82E-19
LINC01208	3.655708008	6.741236581	2.08E-19	1.07E-18
MNX1-AS2	2.58249139	5.18372087	2.25E-19	1.15E-18
LINC02130	2.245315961	4.987121649	2.44E-19	1.25E-18
LINC02725	2.599616748	3.776176867	2.58E-19	1.32E-18
LINC02611	1.451595043	7.542223626	2.65E-19	1.35E-18
PWRN1	-2.389890081	2.933038588	2.76E-19	1.41E-18
LINC02242	-2.177272776	3.053874998	3.28E-19	1.67E-18
TTC3-AS1	1.085126896	4.640323933	3.69E-19	1.87E-18
LINC01146	1.206028385	5.275937245	4.01E-19	2.02E-18
MYCNOS	2.374153615	4.502519259	4.02E-19	2.02E-18
ADAMTS19-AS1	2.52561383	5.056800843	4.89E-19	2.45E-18
LINC00844	-2.181710815	4.78768167	4.98E-19	2.50E-18
LINC02487	3.371394499	8.331613363	5.03E-19	2.52E-18
CTD-3080P12.3	-1.557317548	3.283666194	5.89E-19	2.94E-18
UCKL1-AS1	1.161839531	6.929242491	5.91E-19	2.95E-18
LINC00545	-1.705338611	3.097164691	6.30E-19	3.14E-18
HAR1B	2.928436384	6.089746578	7.37E-19	3.66E-18
INSYN1-AS1	4.170070097	5.296233445	7.52E-19	3.73E-18
LINC00891	-1.04048085	4.020437831	7.78E-19	3.85E-18
LINC00461	3.637816447	6.20679917	9.25E-19	4.57E-18
LINC00987	-1.004386518	8.546714254	1.14E-18	5.62E-18
EML4-AS1	1.633194778	4.347027274	1.40E-18	6.87E-18
ZFHX4-AS1	-1.100135042	6.371932531	1.54E-18	7.57E-18
LINC01727	2.315954129	3.565672874	1.55E-18	7.58E-18
LINC01385	4.36762989	4.133173137	1.61E-18	7.88E-18

LINC01132	1.080020582	8.211497995	1.66E-18	8.11E-18
LINC00898	2.778946979	4.950147717	1.76E-18	8.59E-18
LINC00603	-1.805458962	3.054350536	1.94E-18	9.42E-18
KIF26B-AS1	3.279797999	3.575342663	2.25E-18	1.09E-17
LINC01665	4.647418043	5.069397823	2.29E-18	1.11E-17
LINC02550	-1.094803115	4.615270623	2.38E-18	1.15E-17
MIRLET7A1HG	-1.038660698	5.092114323	2.63E-18	1.27E-17
PRSS30P	1.38285893	7.162973819	3.43E-18	1.65E-17
LINC01358	-1.209109117	3.507140181	3.47E-18	1.67E-17
RBAKDN	2.943843434	3.864808173	4.42E-18	2.12E-17
DDX59-AS1	1.681490423	5.82834297	4.84E-18	2.31E-17
MTOR-AS1	-1.560403846	3.042497388	5.00E-18	2.39E-17
LINC01833	3.300527877	8.50655167	5.58E-18	2.66E-17
WARS2-IT1	-1.327409592	5.5277798	6.26E-18	2.97E-17
PDE2A-AS2	1.13672662	5.240846868	7.28E-18	3.45E-17
BCAN-AS1	2.238329217	9.211515323	7.85E-18	3.72E-17
CLMAT3	-1.074882687	4.701528483	7.92E-18	3.74E-17
UCA1	2.605154009	8.299194658	8.26E-18	3.90E-17
OVOL1-AS1	1.396532562	4.40978188	8.30E-18	3.91E-17
LINC02720	2.407411432	4.292567783	9.03E-18	4.24E-17
NFE4	4.746324582	8.139373756	9.79E-18	4.58E-17
LINC00632	-1.381352891	6.058264673	1.06E-17	4.94E-17
NR4A1AS	-1.236583221	7.041247259	1.09E-17	5.06E-17
PSLNR	5.49299839	4.865597123	1.14E-17	5.29E-17
LINC01099	-1.831446331	2.851928121	1.43E-17	6.62E-17
LINC00974	-1.477001476	3.303029946	1.50E-17	6.93E-17
LINC01788	1.849518324	4.906547938	1.59E-17	7.37E-17
FEZF1-AS1	3.099032294	5.527213498	1.71E-17	7.88E-17
HOXC-AS2	1.349785539	7.433087316	1.76E-17	8.12E-17
ZNF516-DT	1.23929214	6.430678342	1.95E-17	8.98E-17
SCUBE1-AS2	3.930173961	5.15370167	2.17E-17	9.94E-17
LINC02757	-1.255487413	2.906555972	2.24E-17	1.02E-16
SLC4A8-AS1	2.30606373	3.598330047	2.25E-17	1.03E-16
C5orf34-AS1	1.861431262	5.414197943	3.12E-17	1.42E-16
HOXB-AS3	2.784687799	8.734155847	3.53E-17	1.60E-16
LINC01648	2.397828929	3.005969672	3.55E-17	1.61E-16
NAV2-AS6	-1.172878153	7.030143429	3.63E-17	1.64E-16
LINC02533	2.14398526	3.820334273	4.00E-17	1.81E-16
DIP2C-AS1	-1.266453443	6.621273536	4.26E-17	1.92E-16
LINC01863	2.203437745	5.241284203	4.70E-17	2.12E-16
THSD4-AS1	2.860034651	5.223803503	5.00E-17	2.24E-16
MAPT-IT1	2.479932077	7.202066041	5.10E-17	2.28E-16
LINC01013	-1.003564614	5.445357078	5.32E-17	2.37E-16
C5orf64	-1.520646296	5.022115145	5.53E-17	2.46E-16
IL12A-AS1	1.249446087	5.089771522	5.66E-17	2.51E-16
LINC02551	2.761544616	6.78020769	5.66E-17	2.51E-16
STPG3-AS1	1.262732884	6.474400414	6.01E-17	2.66E-16
VAC14-AS1	1.071551672	4.683601116	6.38E-17	2.83E-16
C5orf38	1.626272859	11.38736989	7.97E-17	3.52E-16
FAM230G	2.810832433	3.363519409	9.99E-17	4.40E-16
FRGCA	2.716639829	3.856829329	1.01E-16	4.45E-16

LINC01970	1.097524082	4.696348972	1.03E-16	4.54E-16
LINC02636	-1.360585444	3.393190229	1.14E-16	5.00E-16
LINC02571	2.132373713	5.195683633	1.14E-16	5.01E-16
SLX1A-SULT1A3	1.306817821	3.882965477	1.24E-16	5.40E-16
LINC01010	-1.326238298	5.651519118	1.27E-16	5.55E-16
LINC01655	1.813613372	4.095944894	1.37E-16	5.94E-16
IL21R-AS1	1.454877494	4.512192891	1.41E-16	6.13E-16
DIRC1	1.439604972	4.957342844	1.65E-16	7.16E-16
INHBA-AS1	1.247151386	5.375316091	1.73E-16	7.51E-16
TDRG1	2.556473588	3.939223994	1.78E-16	7.72E-16
DPP4-DT	-1.282456863	3.668135767	1.79E-16	7.72E-16
SNAP47-AS1	1.380546736	3.725632009	1.85E-16	7.97E-16
NAMA	-1.339983612	3.759784509	2.00E-16	8.60E-16
LINC00540	-1.23605937	4.841699539	2.43E-16	1.04E-15
GACAT2	3.188215521	5.786466258	2.46E-16	1.05E-15
LINC01979	1.384602181	5.636364893	2.48E-16	1.06E-15
LINC02568	1.866080421	10.84397469	3.03E-16	1.29E-15
RORB-AS1	-1.709209666	2.965825202	3.16E-16	1.34E-15
LHX1-DT	5.435387578	5.679174803	3.18E-16	1.35E-15
C1orf147	1.014813733	5.155996422	3.51E-16	1.48E-15
LINC02028	1.056271778	6.668497125	3.85E-16	1.63E-15
GACAT3	2.32963326	4.412892549	3.95E-16	1.67E-15
BRWD1-AS1	1.236179503	4.407170219	4.33E-16	1.83E-15
MIR222HG	-1.019496063	7.686651083	4.65E-16	1.96E-15
TRPM2-AS	1.606270253	9.089623998	5.18E-16	2.18E-15
LINC01998	2.515446631	3.71915011	5.44E-16	2.28E-15
LINC01266	-1.51023796	5.373558991	5.45E-16	2.28E-15
DGCR5	1.492441035	5.986340073	5.76E-16	2.41E-15
ANK3-DT	1.591368016	4.625911837	6.20E-16	2.59E-15
LINC01556	1.643154031	4.498707789	6.60E-16	2.74E-15
LINC00517	-1.075501262	4.975343642	6.67E-16	2.77E-15
MGC15885	-1.697709839	3.12677161	7.94E-16	3.28E-15
LINC02099	2.301620151	6.123823091	8.48E-16	3.50E-15
LINC00896	1.482386551	5.416598602	8.49E-16	3.50E-15
LINC01956	3.192146965	7.910649047	1.00E-15	4.12E-15
LINC01287	3.416403806	7.603841434	1.05E-15	4.31E-15
LINC00668	5.294782317	9.108270045	1.10E-15	4.52E-15
LINC01546	1.148809329	3.72896858	1.14E-15	4.66E-15
LINC01555	2.052855976	3.594635104	1.16E-15	4.71E-15
LINC00518	4.214192181	6.005004041	1.18E-15	4.82E-15
P3H2-AS1	-1.643314016	3.882213637	1.24E-15	5.03E-15
FAM153CP	1.421450743	4.917849732	1.51E-15	6.15E-15
COLCA1	-1.27624585	9.037608355	1.56E-15	6.34E-15
LINC02554	-1.420370725	3.007583881	1.74E-15	7.05E-15
MEIS1-AS2	-1.224455775	2.945257678	1.74E-15	7.05E-15
FAM83A-AS1	2.554506113	5.093179601	1.83E-15	7.38E-15
FLJ31356	1.096373372	5.879712934	1.95E-15	7.84E-15
LINC00524	2.76011507	3.747124687	2.04E-15	8.20E-15
LINC01239	-1.410917804	6.808358561	2.11E-15	8.45E-15
LINC01592	2.148538353	3.474643108	2.16E-15	8.65E-15
GPRACR	1.35870356	3.784174713	2.23E-15	8.92E-15

LINC02574	1.8510507	3.762312437	2.24E-15	8.94E-15
BCRP3	1.236530551	6.449726429	2.36E-15	9.41E-15
LINC00639	-1.129200582	7.741348117	2.76E-15	1.10E-14
LINC01293	2.307935574	5.409331436	3.10E-15	1.23E-14
FAM242C	1.174405314	4.470355682	3.37E-15	1.33E-14
NECTIN4-AS1	1.477071887	4.12642959	3.39E-15	1.34E-14
LINC01558	-1.02061517	6.5187731	3.48E-15	1.37E-14
BSN-DT	1.690475612	4.231283783	4.18E-15	1.64E-14
HPYR1	2.190045934	3.197029053	4.20E-15	1.65E-14
ETV7-AS1	1.438033651	4.362394478	4.39E-15	1.72E-14
USP2-AS1	1.24205815	6.43194515	4.76E-15	1.86E-14
CRAT37	2.805368099	5.347152072	5.07E-15	1.98E-14
LINC02247	4.606360417	4.926667918	5.09E-15	1.98E-14
LINC01811	3.493128236	4.186260379	5.77E-15	2.24E-14
UMODL1-AS1	2.176544094	4.998251709	6.01E-15	2.33E-14
HCG20	1.066111169	4.829234579	6.06E-15	2.35E-14
STAG1-DT	1.443294334	3.446538767	6.27E-15	2.43E-14
LINC01389	1.073282743	5.803318096	6.30E-15	2.44E-14
NCKAP5-AS2	-1.109058672	4.680531316	6.73E-15	2.59E-14
PPP1R21-DT	1.035097407	4.987398401	7.03E-15	2.70E-14
LINC01883	-1.98075794	3.60774166	7.18E-15	2.75E-14
ETV5-AS1	-1.234969029	3.377950899	8.09E-15	3.09E-14
LINC02516	1.364085662	5.000570817	8.29E-15	3.16E-14
FAM181A-AS1	-1.6921956	2.951498656	8.33E-15	3.17E-14
LINC01357	1.605205119	4.636293641	8.40E-15	3.19E-14
MECOM-AS1	-1.301581202	3.177535	8.77E-15	3.33E-14
COL25A1-DT	-1.123111461	2.917821413	9.03E-15	3.43E-14
LINC02345	1.31934034	5.76743283	9.98E-15	3.78E-14
KCNMB2-AS1	2.863266771	6.651823214	1.00E-14	3.79E-14
LINC02234	-1.169916529	3.965964294	1.09E-14	4.11E-14
LINC01297	4.120752779	4.120864796	1.15E-14	4.34E-14
LINC02438	3.53677944	3.763837296	1.25E-14	4.70E-14
C8orf86	2.042394938	5.97915056	1.26E-14	4.73E-14
C8orf37-AS1	1.070335797	5.94025698	1.32E-14	4.97E-14
SUGCT-AS1	1.468792879	5.667634181	1.33E-14	4.97E-14
VAV3-AS1	1.90006469	4.195794237	1.37E-14	5.13E-14
LINC01121	1.055525652	6.021660726	1.74E-14	6.50E-14
LINC02036	1.408680286	7.163681025	1.85E-14	6.88E-14
LINC02254	-1.96412711	2.98553791	2.11E-14	7.86E-14
PINCR	4.272872317	4.409721007	2.12E-14	7.88E-14
LINC00494	1.890922871	5.898633692	2.18E-14	8.07E-14
DRAIC	1.631402402	9.699815887	2.20E-14	8.14E-14
LINCR-0001	-1.128689178	6.12145027	2.35E-14	8.69E-14
IL21-AS1	2.067275819	4.167846476	2.52E-14	9.31E-14
LINC02446	1.766652899	6.756980206	2.74E-14	1.01E-13
LINC02417	2.105983978	3.169075475	2.85E-14	1.05E-13
LINC02351	-1.805221557	3.834296945	2.89E-14	1.06E-13
LINC01771	1.542433871	3.735192022	2.98E-14	1.09E-13
GS1-24F4.2	-1.390553244	4.150653154	3.01E-14	1.10E-13
GSTCD-AS1	1.040251032	4.181517882	3.13E-14	1.14E-13
TMEM105	1.488046152	7.340910703	3.50E-14	1.27E-13

LINC02433	3.571647174	6.08348469	3.90E-14	1.42E-13
MIR4713HG	3.440780881	3.838015098	4.01E-14	1.46E-13
LINC02761	-1.026816428	5.14667193	4.16E-14	1.51E-13
LINC02560	1.613926966	7.056718927	4.40E-14	1.60E-13
SLC9A3-AS1	1.013491611	10.1204485	4.69E-14	1.70E-13
LINC01410	1.139403635	9.272506801	4.96E-14	1.79E-13
LINC01953	4.134845819	3.188691396	5.31E-14	1.92E-13
LINC02830	3.263707874	4.658370315	6.15E-14	2.22E-13
SILC1	2.679473884	6.782766873	6.63E-14	2.38E-13
CARHSP1-DT	1.521369989	3.256204046	7.97E-14	2.86E-13
PLAC4	2.186323998	7.435440494	8.05E-14	2.88E-13
LINC02077	5.610456708	4.182434022	8.88E-14	3.17E-13
TMEM92-AS1	1.168978861	5.4736816	9.04E-14	3.22E-13
LINC00613	4.885126853	3.638050276	1.10E-13	3.88E-13
ZNF474-AS1	1.539913187	3.651698629	1.11E-13	3.94E-13
HOXB-AS4	2.883281056	3.852160901	1.25E-13	4.41E-13
LSINCT5	-1.313420539	4.857235484	1.30E-13	4.60E-13
LINC02147	-1.452991479	3.591392267	1.31E-13	4.61E-13
LINC01022	2.760503534	2.992541182	1.32E-13	4.64E-13
LINC02518	-1.420842587	4.240272874	1.36E-13	4.79E-13
LINC02208	1.814656898	4.326075173	1.37E-13	4.80E-13
FER1L6-AS2	4.046948487	3.878511563	1.71E-13	5.97E-13
SLC7A11-AS1	1.755943045	4.19653994	1.83E-13	6.37E-13
LINC02520	1.679818535	3.895453503	1.84E-13	6.39E-13
LINC01647	3.063048255	3.037869439	1.88E-13	6.53E-13
PTCHD1-AS	-1.560308626	3.045648388	1.90E-13	6.58E-13
IRAIN	1.847663532	6.06307776	2.05E-13	7.10E-13
NDP-AS1	3.073986121	3.591232681	2.06E-13	7.11E-13
SIX3-AS1	3.117959308	4.75267938	2.06E-13	7.12E-13
FLJ16779	1.842270047	5.875802663	2.14E-13	7.38E-13
RYR3-DT	-1.873060046	3.651924964	2.30E-13	7.87E-13
ASH1L-IT1	1.686581765	3.055829313	2.35E-13	8.04E-13
CSTF3-DT	1.08892029	4.025763941	2.50E-13	8.53E-13
USP7-AS1	1.042796476	3.876128787	2.68E-13	9.12E-13
LINC01926	2.602969174	2.919236981	2.72E-13	9.25E-13
LINC00452	1.623116976	3.478317934	2.74E-13	9.30E-13
LINC01087	2.188270807	10.39588366	3.09E-13	1.05E-12
LINC02245	1.620895402	3.905987632	3.28E-13	1.11E-12
CDKN2A-DT	2.015946204	3.815731048	3.53E-13	1.19E-12
LINC02073	1.249054903	5.133290162	3.56E-13	1.20E-12
LINC02539	1.926978674	2.813138849	3.67E-13	1.24E-12
LINC02475	3.007640415	5.838306512	4.32E-13	1.45E-12
FAM230C	5.115939048	4.164277805	4.44E-13	1.49E-12
FRY-AS1	1.349163773	4.324351033	4.65E-13	1.56E-12
LINC02015	1.91244503	7.553335723	4.67E-13	1.56E-12
LNCAROD	2.675616631	5.856894419	4.82E-13	1.61E-12
PBX1-AS1	2.013039061	4.058841532	4.99E-13	1.67E-12
MAGEA4-AS1	5.52566303	4.480690449	5.57E-13	1.85E-12
LINC01163	1.722522152	3.715797478	5.70E-13	1.89E-12
VPS9D1-AS1	1.11510405	9.694832486	6.18E-13	2.05E-12
CATIP-AS2	1.757289639	3.330841369	6.37E-13	2.11E-12

PKP4-AS1	1.061229221	7.579514752	6.57E-13	2.17E-12
TPRG1-AS2	2.037559463	4.224100643	7.02E-13	2.31E-12
LINC01351	3.245740613	5.592779676	7.55E-13	2.48E-12
SCG5-AS1	1.580343348	3.049304465	7.78E-13	2.56E-12
MYO3B-AS1	3.215839392	4.097156352	7.97E-13	2.62E-12
FOXP4-AS1	1.169666089	7.071109539	7.99E-13	2.62E-12
MDS2	1.305044276	4.214775178	8.32E-13	2.72E-12
LINC00504	1.435721805	11.03393031	8.65E-13	2.82E-12
LINC00165	-1.362383816	4.377390596	9.02E-13	2.93E-12
LINC02197	3.795601272	5.583016624	9.22E-13	2.99E-12
MIR181A1HG	1.475631688	4.061852987	9.50E-13	3.08E-12
MIR7-3HG	3.85353535	5.140193195	9.66E-13	3.13E-12
LINC01740	1.69428771	4.389307561	1.02E-12	3.30E-12
LINC01234	2.821995829	5.346373961	1.03E-12	3.32E-12
LINC02679	-1.136659233	2.946548965	1.05E-12	3.41E-12
LINC02112	2.146891231	3.04347012	1.09E-12	3.52E-12
PIK3CD-AS1	1.189742314	4.139831524	1.10E-12	3.53E-12
DLG1-AS1	1.029081037	5.374385166	1.10E-12	3.55E-12
LINC01214	1.780730772	3.810550578	1.13E-12	3.63E-12
LINC01983	2.496604122	4.746990989	1.13E-12	3.64E-12
LINC00158	1.52552369	4.061468024	1.18E-12	3.78E-12
IDH2-DT	1.541702886	4.718618162	1.19E-12	3.81E-12
LINC02874	2.019034151	3.110793476	1.42E-12	4.51E-12
DELEC1	1.787715356	5.656953914	1.50E-12	4.76E-12
LINC02418	3.310815057	4.289937418	1.57E-12	4.98E-12
LINC01606	3.757283517	5.812027748	1.57E-12	4.99E-12
LINC02450	1.610952337	3.967046611	1.61E-12	5.09E-12
LINC00305	4.680615534	3.702245733	1.61E-12	5.10E-12
KCNK15-AS1	1.032379014	6.77687595	1.64E-12	5.19E-12
LINC00299	1.064546512	5.055629209	1.75E-12	5.51E-12
LURAP1L-AS1	1.589588747	4.260393186	1.83E-12	5.75E-12
LINC02542	1.480432877	6.416175144	2.02E-12	6.35E-12
GTSCR1	2.041828001	3.133434008	2.06E-12	6.47E-12
LINC01419	6.870677894	6.03622285	2.07E-12	6.47E-12
LINC02705	-1.066327004	3.194092563	2.16E-12	6.76E-12
MAPT-AS1	2.214981096	7.405140559	2.37E-12	7.41E-12
LINC02014	1.431380984	5.125482774	2.51E-12	7.83E-12
LINC02097	-1.162580118	4.506971942	2.61E-12	8.12E-12
FARSA-AS1	1.821237728	3.431769329	2.94E-12	9.09E-12
C2orf91	1.242965297	4.486179714	2.96E-12	9.12E-12
LINC02278	1.616458047	3.536320204	2.96E-12	9.13E-12
LINC02880	-1.417069957	4.566609424	3.51E-12	1.08E-11
LINC01879	2.692023543	3.207341324	3.56E-12	1.09E-11
MIR378D2HG	1.101899014	3.99331718	3.66E-12	1.13E-11
LINC00392	5.814607994	4.712139353	3.99E-12	1.22E-11
LINC02100	1.453897993	3.84600602	4.13E-12	1.26E-11
LINC02324	1.756054436	2.994736089	4.52E-12	1.38E-11
LINC01303	1.219327489	5.315689871	4.80E-12	1.47E-11
LINC02129	2.992254546	3.447782293	4.83E-12	1.47E-11
LINC01271	1.066267347	5.315888618	5.12E-12	1.56E-11
LINC02875	1.161158321	5.980902314	5.33E-12	1.62E-11

LINC01513	2.017015231	3.757300958	5.38E-12	1.63E-11
LINC01445	4.833017606	3.741912579	5.94E-12	1.80E-11
LINC02054	2.036499438	4.134894943	5.96E-12	1.80E-11
BFSP2-AS1	1.958495658	5.352035496	6.10E-12	1.84E-11
LINC01583	1.82634788	3.102792935	6.44E-12	1.95E-11
VSTM2A-OT1	4.409483764	6.872639117	7.67E-12	2.30E-11
LINC01653	5.790282796	4.716625647	8.06E-12	2.42E-11
ELDR	2.938532107	3.924254506	8.11E-12	2.43E-11
C1QTNF1-AS1	-1.235333458	3.888045636	8.83E-12	2.64E-11
LINC02868	2.282218403	2.875750631	9.87E-12	2.94E-11
LINC02506	3.843075313	5.994343882	1.01E-11	3.00E-11
LINC02067	1.508014154	4.17533519	1.02E-11	3.02E-11
MIR663AHG	3.305088176	3.714098944	1.14E-11	3.38E-11
LINC01518	4.170818185	3.731632958	1.29E-11	3.81E-11
LINC00930	1.273712881	7.122255643	1.41E-11	4.14E-11
LINC00322	1.261055912	3.830654145	1.41E-11	4.14E-11
AADACL2-AS1	-1.082766009	4.645008632	1.48E-11	4.36E-11
LINC02753	-1.312154633	3.049199796	1.55E-11	4.55E-11
LINC00992	2.310327296	7.216065276	1.63E-11	4.77E-11
LINC02273	1.329379839	6.125434531	1.70E-11	4.96E-11
LINC00365	1.300604403	4.926464511	1.71E-11	4.98E-11
LINC00210	4.639222539	3.555911365	1.77E-11	5.17E-11
FGF12-AS3	-1.209318082	3.267137722	1.81E-11	5.27E-11
LINC00221	3.877381021	5.615449011	1.90E-11	5.53E-11
LINC01479	-1.164659557	3.026020145	1.97E-11	5.73E-11
DNM3-IT1	1.281777759	3.07157817	2.00E-11	5.81E-11
LINC01283	1.614446615	2.906732584	2.12E-11	6.13E-11
C8orf31	1.333701825	7.797468584	2.12E-11	6.13E-11
MYB-AS1	1.332783977	4.256328667	2.21E-11	6.38E-11
NAALADL2-AS2	2.872115624	4.706711821	2.21E-11	6.38E-11
LINC02577	2.437031018	4.156793629	2.43E-11	7.01E-11
C7orf77	2.545674374	3.487190581	2.53E-11	7.29E-11
COL4A2-AS2	-1.273978078	3.498144012	2.76E-11	7.93E-11
LINCR-0003	-1.789588216	3.878522802	2.77E-11	7.95E-11
LINC02332	1.529994446	3.974776621	3.21E-11	9.18E-11
LINC02774	1.680087242	3.869786138	3.61E-11	1.03E-10
TBL1XR1-AS1	1.456772516	3.765565813	3.92E-11	1.11E-10
C5orf60	1.039977833	3.871456593	4.03E-11	1.14E-10
C17orf102	2.950380198	4.05472523	4.10E-11	1.16E-10
TRBV11-2	1.343638177	5.07037558	4.13E-11	1.17E-10
LINC01508	1.83210095	6.01952057	4.15E-11	1.17E-10
LINC02003	2.876649485	3.190833804	4.38E-11	1.24E-10
LINC01896	3.141179622	3.358682544	4.56E-11	1.28E-10
ATXN8OS	4.540163568	4.019794723	4.58E-11	1.29E-10
PEX5L-AS1	2.506006289	2.870738957	5.22E-11	1.46E-10
LINC02463	1.487251061	4.027404804	5.27E-11	1.48E-10
LINC02046	3.232821347	3.323478382	5.49E-11	1.54E-10
TLR8-AS1	2.154420117	3.582473585	5.68E-11	1.59E-10
LINC01694	1.693139904	6.619123829	5.77E-11	1.61E-10
LINC02159	2.015589393	5.13458196	6.37E-11	1.77E-10
LL22NC03-63E9.3	2.396751691	3.766865706	6.48E-11	1.80E-10

LINC00867	2.351420802	4.912859319	7.00E-11	1.94E-10
FOXCUT	2.729785146	6.779805391	7.33E-11	2.03E-10
LINC02335	4.028072588	3.260525088	7.90E-11	2.18E-10
MIR4300HG	2.29649717	3.767226379	8.16E-11	2.25E-10
LMNB1-DT	1.541728114	2.84613156	8.33E-11	2.29E-10
SAMSN1-AS1	1.590666407	2.890197631	8.44E-11	2.32E-10
LINC02223	1.555916527	3.486526544	8.62E-11	2.37E-10
CEACAM16-AS1	1.106575324	7.712322381	9.02E-11	2.47E-10
LINC01425	3.992961855	3.483223298	9.06E-11	2.48E-10
LINC02389	1.193652759	5.167552488	9.27E-11	2.53E-10
ADORA2A-AS1	1.064886904	6.123597774	9.37E-11	2.56E-10
HAR1A	1.574952383	7.460420244	9.56E-11	2.61E-10
LINC02163	3.219008843	3.924630423	1.04E-10	2.83E-10
HOXA11-AS	1.283288941	6.374071055	1.16E-10	3.16E-10
LINC01446	3.795092658	5.287134151	1.20E-10	3.27E-10
LINC01411	2.058396435	7.954464722	1.34E-10	3.63E-10
GATA2-AS1	1.198719463	8.693724375	1.34E-10	3.65E-10
LINC02253	3.66594102	4.70008717	1.45E-10	3.93E-10
LINC01940	-1.04354872	3.409213099	1.46E-10	3.95E-10
NRIR	1.145130718	5.618221848	1.69E-10	4.58E-10
LINC00544	1.818050856	3.553941259	1.70E-10	4.60E-10
LINC01674	2.448567587	2.801426418	1.73E-10	4.67E-10
LINC00261	5.786619694	6.255183511	1.76E-10	4.74E-10
LINC02752	1.222801086	3.968905821	1.80E-10	4.86E-10
LINC00824	2.192947294	4.502656	1.83E-10	4.92E-10
DLX6-AS1	1.927947254	6.338568109	2.08E-10	5.58E-10
LINC01342	1.19489548	5.615150662	2.09E-10	5.61E-10
LINC01633	3.570578793	3.256257853	2.14E-10	5.72E-10
LINC01924	3.524328032	4.52319727	2.14E-10	5.73E-10
LINC00923	-1.253087177	3.860993271	2.29E-10	6.11E-10
LINC02882	3.136868648	4.124246509	2.38E-10	6.34E-10
C8orf87	3.27055311	4.424322864	2.58E-10	6.87E-10
LINC02694	1.403475063	3.253283579	2.76E-10	7.34E-10
SOX9-AS1	-1.083438474	9.84903727	2.79E-10	7.39E-10
LINC01215	1.468687468	6.498052677	3.24E-10	8.55E-10
LINC02377	4.517868742	3.786541105	3.28E-10	8.62E-10
LINC02515	-1.304563632	6.159229624	3.65E-10	9.59E-10
RMRP	4.097958735	4.801802175	3.95E-10	1.04E-09
LINC01973	1.281942235	3.498470079	4.12E-10	1.08E-09
LINC00944	1.187614889	5.633436207	4.38E-10	1.14E-09
CLLU1-AS1	1.931114086	4.649626017	4.53E-10	1.18E-09
CELF2-AS1	-1.029224519	5.043735959	4.95E-10	1.29E-09
LINC02732	2.855442188	4.403518868	5.04E-10	1.31E-09
LINC01781	1.670205436	5.024409966	5.31E-10	1.38E-09
GRIK1-AS1	-1.245673825	6.966692195	5.94E-10	1.54E-09
LINC01489	1.365204408	5.623448198	5.98E-10	1.55E-09
PACRG-AS1	-1.141544944	3.600983431	6.00E-10	1.55E-09
C2-AS1	1.076340742	4.134038953	6.03E-10	1.56E-09
LINC00858	3.076823985	5.164516452	6.10E-10	1.57E-09
HUNK-AS1	2.028589859	3.048621032	6.16E-10	1.59E-09
LINC01475	2.992685843	2.878261345	6.17E-10	1.59E-09

LINC00563	1.614080412	3.374103025	6.29E-10	1.62E-09
LINC02707	1.400866	3.722936299	6.31E-10	1.62E-09
SKAP1-AS1	1.208002983	3.484076348	6.57E-10	1.69E-09
LIVAR	1.538928382	4.506973413	6.96E-10	1.78E-09
LINC02037	1.859706675	3.631527277	7.19E-10	1.84E-09
LINC01664	1.11420793	4.026988978	7.37E-10	1.88E-09
POU6F2-AS2	3.59237251	3.508825562	8.13E-10	2.07E-09
LINC01675	1.651885741	3.086120028	8.21E-10	2.09E-09
LINC02484	3.928770554	3.176802208	8.99E-10	2.28E-09
LINC00564	5.010635698	4.086296769	9.76E-10	2.47E-09
LINC02055	2.598170058	5.415713512	1.04E-09	2.62E-09
NCKAP5-IT1	1.585511984	2.95547887	1.05E-09	2.64E-09
LINC02521	1.295453586	3.300791203	1.06E-09	2.68E-09
DSCR4	3.695757424	3.253407465	1.13E-09	2.84E-09
BANCR	2.432126206	5.443268761	1.15E-09	2.90E-09
LINC02074	2.591114991	2.840829603	1.22E-09	3.05E-09
LINC00589	1.879606767	6.48507619	1.23E-09	3.08E-09
BARX1-DT	2.885444084	3.312630226	1.25E-09	3.14E-09
LINC01574	3.156102415	3.23045357	1.30E-09	3.26E-09
ARHGAP28-AS1	-1.007662497	4.081594031	1.31E-09	3.26E-09
LINC02816	1.102325162	3.407030743	1.45E-09	3.60E-09
LINC02860	-1.122199477	2.777060133	1.46E-09	3.63E-09
LINC00113	2.287304555	3.574119652	1.48E-09	3.67E-09
GRAMD1A-AS1	1.040215414	3.353865072	1.57E-09	3.87E-09
LINC00520	1.510306457	3.641157703	1.57E-09	3.87E-09
LINC02809	1.016835524	4.127536252	1.58E-09	3.91E-09
LINC02154	1.836826454	4.376374481	1.60E-09	3.95E-09
SDAD1-AS1	1.377600523	3.027597316	1.72E-09	4.24E-09
LINC01470	2.297676863	3.300771425	1.78E-09	4.38E-09
LINC01906	2.187556787	3.675861538	1.93E-09	4.72E-09
LINC02466	3.2710377	3.18632145	1.93E-09	4.74E-09
LINC01124	1.336484986	7.36084269	2.02E-09	4.93E-09
LINC02241	3.134418415	3.108018997	2.15E-09	5.26E-09
RBMS3-AS2	-1.108302245	3.375642441	2.16E-09	5.28E-09
LINC02523	2.751977326	3.601788996	2.31E-09	5.63E-09
LINC01414	2.878937519	3.00274485	2.41E-09	5.85E-09
LINC02380	2.480732673	2.870285745	2.58E-09	6.25E-09
LINC01992	3.470506223	4.776907517	2.70E-09	6.54E-09
LINC01436	1.4842826	8.791389494	2.76E-09	6.70E-09
LINC01716	4.464950284	4.461751223	2.79E-09	6.75E-09
LINC02378	3.777107348	3.596377644	2.80E-09	6.78E-09
MIR548XHG	4.095973004	4.452292881	2.88E-09	6.95E-09
LINC00200	3.470084301	4.351400563	3.02E-09	7.29E-09
LINC01269	1.335504066	5.125086721	3.26E-09	7.86E-09
PAQR5-DT	1.402443257	4.923526535	3.45E-09	8.30E-09
LINC02869	1.700988877	3.521343888	3.51E-09	8.43E-09
SMYD3-IT1	1.623523741	3.040125294	3.54E-09	8.51E-09
BBOX1-AS1	1.166896174	5.317415544	3.66E-09	8.79E-09
LINC01792	1.235585758	4.290634714	3.96E-09	9.49E-09
LINC01764	1.134206535	3.442985836	4.18E-09	1.00E-08
LINC00402	1.553732931	5.193199677	4.69E-09	1.12E-08

LINC01891	-1.091220557	3.344325783	5.24E-09	1.24E-08
LINC01559	2.109190408	4.167545523	5.36E-09	1.27E-08
LINC02561	1.014549383	4.07260523	5.74E-09	1.36E-08
LINC02068	1.088871447	5.333915982	5.82E-09	1.38E-08
SLC2A9-AS1	1.170743329	4.879734254	6.21E-09	1.47E-08
LINC02641	1.325153461	3.770543018	6.36E-09	1.50E-08
LINC01014	3.379679118	3.528498333	6.43E-09	1.51E-08
FOXF2-DT	1.348638041	3.530816107	7.22E-09	1.69E-08
LINC00244	1.093756662	3.265279674	7.30E-09	1.71E-08
BICRA-AS1	1.849504932	3.169543013	7.44E-09	1.74E-08
LINC01107	1.257420902	3.563433868	9.02E-09	2.10E-08
LINC02167	4.732700716	5.629378534	9.65E-09	2.25E-08
OPCML-IT1	4.142422037	3.389364642	9.74E-09	2.27E-08
LINC02576	1.029272013	5.209121855	9.75E-09	2.27E-08
VCAN-AS1	1.154403371	3.945026638	1.01E-08	2.35E-08
LINC00567	1.051611831	3.977208249	1.03E-08	2.38E-08
MIMT1	1.773777133	4.965738345	1.10E-08	2.56E-08
RSF1-IT1	1.19870162	3.495018783	1.15E-08	2.67E-08
RABGAP1L-AS1	1.550398916	3.139139546	1.16E-08	2.68E-08
ADARB2-AS1	3.39066824	5.440798778	1.27E-08	2.93E-08
LINC02579	1.222309046	3.492840459	1.28E-08	2.94E-08
LINC00709	4.084429367	3.502658538	1.31E-08	3.01E-08
LINC02365	1.421043014	3.150216696	1.31E-08	3.01E-08
DLGAP1-AS3	2.212886931	3.340768386	1.33E-08	3.06E-08
LINC02437	2.346713651	4.890540009	1.40E-08	3.20E-08
LINC01843	1.235540682	7.174089881	1.45E-08	3.32E-08
POTEF-AS1	2.417539202	3.995804073	1.49E-08	3.40E-08
SPATA3-AS1	1.227554428	4.620252892	1.52E-08	3.48E-08
LINC02588	2.626005497	4.448860168	1.54E-08	3.52E-08
MGC32805	1.852216134	5.292645525	1.59E-08	3.64E-08
LINC01993	1.056501358	4.236963535	1.64E-08	3.74E-08
NAV2-AS3	1.234390414	3.574641446	1.76E-08	3.99E-08
LINC02837	2.474308581	2.893947397	1.77E-08	4.02E-08
LINC01490	3.853988484	3.643178011	1.83E-08	4.16E-08
LINC01501	2.155952924	3.150080363	1.89E-08	4.28E-08
CASC20	2.239689846	3.166490113	1.89E-08	4.29E-08
SIDT1-AS1	1.050011609	4.12311792	1.94E-08	4.40E-08
NECTIN3-AS1	-1.043160405	4.192563478	1.98E-08	4.46E-08
LINC02308	-1.133238961	4.395860993	2.11E-08	4.77E-08
LINC00626	1.418425635	3.460802818	2.30E-08	5.18E-08
LINC01726	1.463320662	2.70730803	2.55E-08	5.74E-08
LINC02109	2.848363334	3.541238961	2.56E-08	5.75E-08
SCGB1B2P	1.651627549	9.496819725	2.60E-08	5.84E-08
KU-MEL-3	1.126520796	4.746638928	2.61E-08	5.86E-08
GDF5-AS1	-1.043632853	3.003392901	2.66E-08	5.97E-08
PDE6B-AS1	1.487092691	4.665395575	2.75E-08	6.17E-08
LINC00351	2.907681014	2.773074361	2.78E-08	6.23E-08
LINC02783	1.878650499	3.534550369	2.95E-08	6.58E-08
ELFN2	1.902038612	3.139086051	3.07E-08	6.83E-08
PPM1B-DT	1.167450292	2.833641891	3.24E-08	7.21E-08
LINC02473	1.475544076	6.087032783	3.52E-08	7.82E-08

LINC02181	1.975583581	3.443451996	3.72E-08	8.24E-08
LINC01603	1.665044032	3.478669332	4.04E-08	8.91E-08
FILNC1	-1.159380377	4.109958925	4.05E-08	8.93E-08
DISC1FP1	-1.132690276	3.479642966	4.18E-08	9.20E-08
FLJ36000	3.265391353	3.066690636	4.30E-08	9.45E-08
SH3PXD2A-AS1	1.131192534	6.215262657	4.34E-08	9.53E-08
LINGO1-AS1	1.363059257	3.795374786	4.39E-08	9.64E-08
LINC00861	1.00449342	8.191908725	4.48E-08	9.82E-08
SALRNA1	1.216485685	5.665733184	4.79E-08	1.05E-07
LINC01608	3.980910035	3.988600748	4.98E-08	1.09E-07
PEX5L-AS2	2.56610733	2.818971612	5.00E-08	1.09E-07
HHATL-AS1	-1.013544369	3.493973484	5.05E-08	1.10E-07
LINC01016	1.685026411	9.484260256	5.31E-08	1.16E-07
LINC02842	1.432428014	3.371536223	5.49E-08	1.19E-07
IGFL2-AS1	1.574489884	5.350558669	5.76E-08	1.25E-07
C12orf77	1.323276932	2.954771482	6.19E-08	1.34E-07
LUNAR1	1.210185234	4.54879012	6.35E-08	1.37E-07
FOXP1-IT1	1.161947251	4.567646347	7.17E-08	1.55E-07
SLC25A48-AS1	2.004036116	3.031339922	7.37E-08	1.59E-07
LINC01925	3.062803693	3.073828925	7.75E-08	1.67E-07
LINC02432	1.788775404	7.486803465	8.21E-08	1.76E-07
LINC00355	3.017526724	4.598392337	8.35E-08	1.79E-07
LARGE-AS1	1.278846893	3.597943369	9.01E-08	1.92E-07
CACNA2D3-AS1	2.35005482	2.959293803	9.17E-08	1.95E-07
LINC00707	2.251317965	8.116280619	1.01E-07	2.15E-07
LINC00856	1.06827476	4.52799721	1.03E-07	2.18E-07
LINC00602	1.965422789	3.53462464	1.08E-07	2.28E-07
KCNAB1-AS2	1.728724524	2.893884264	1.08E-07	2.29E-07
PCSK6-AS1	1.498252775	2.808342556	1.10E-07	2.33E-07
BZW1-AS1	1.236844309	3.082410947	1.10E-07	2.34E-07
LINC02672	3.29828776	3.333662731	1.12E-07	2.37E-07
LINC01975	1.365167433	2.925096135	1.14E-07	2.41E-07
CASC9	2.687812202	5.904913467	1.21E-07	2.55E-07
LINC01623	1.253554669	3.816810565	1.24E-07	2.61E-07
PANTR1	-1.430468307	3.962065585	1.47E-07	3.09E-07
LINC02616	3.697391474	4.339213409	1.81E-07	3.79E-07
SHANK2-AS1	1.364725562	4.107138618	1.90E-07	3.96E-07
ARLNC1	1.024158	5.15449526	2.01E-07	4.19E-07
LINC01524	-1.057599029	3.771331276	2.05E-07	4.27E-07
IATPR	1.213695725	3.407765471	2.08E-07	4.33E-07
LINC00092	-1.051260267	6.465322981	2.18E-07	4.52E-07
MIR4290HG	1.37075584	2.902250891	2.46E-07	5.09E-07
RUNX2-AS1	1.75435139	2.721890657	2.63E-07	5.42E-07
LINC01980	3.306114981	5.33674256	2.67E-07	5.51E-07
LINC02820	2.996626324	4.620061229	3.00E-07	6.17E-07
ANKRD44-AS1	1.106581084	4.307893347	3.23E-07	6.63E-07
LINC01800	1.0697382	3.487829923	3.29E-07	6.76E-07
LEMD1-DT	1.620197675	3.974894267	3.34E-07	6.84E-07
LINC02492	1.982228889	4.101329872	3.36E-07	6.87E-07
LINC02688	1.132162258	5.537847397	3.49E-07	7.14E-07
LINC02287	2.148393862	3.341474928	3.53E-07	7.21E-07

LINC02141	2.804684236	3.336540009	3.57E-07	7.29E-07
GLIS3-AS1	1.646958694	3.345638657	3.60E-07	7.34E-07
LINC01971	1.446881872	3.863605391	3.61E-07	7.36E-07
LINC01976	1.498868468	3.150343861	3.74E-07	7.62E-07
CXXC5-AS1	1.015494633	3.133994795	3.80E-07	7.74E-07
THRA1/BTR	1.446561817	3.434022798	3.86E-07	7.85E-07
LINC01441	3.057059397	3.085969656	3.92E-07	7.96E-07
LINC02152	1.919073113	2.905831806	3.94E-07	8.00E-07
SATB2-AS1	1.07021082	4.732464154	4.01E-07	8.14E-07
ELOVL2-AS1	1.348138992	6.760670213	4.04E-07	8.19E-07
MIR3681HG	1.083802651	3.876032123	4.34E-07	8.78E-07
SAMMSON	1.315544904	3.672725195	4.43E-07	8.95E-07
LINC00488	2.198611731	2.815085784	4.47E-07	9.03E-07
SND1-IT1	1.095254977	4.070314766	4.52E-07	9.10E-07
LINC02570	2.357907513	3.892043992	4.92E-07	9.89E-07
LINC02293	2.621041287	3.450373133	5.07E-07	1.02E-06
SHANK2-AS2	1.456292111	3.390750576	5.25E-07	1.05E-06
LINC01460	1.346412513	3.900414322	5.80E-07	1.16E-06
LINC01639	3.339672916	5.851013726	5.82E-07	1.17E-06
LINC01587	1.322564527	4.808493954	5.89E-07	1.18E-06
LSAMP-AS1	2.445342137	3.187879799	5.92E-07	1.18E-06
LINC00434	1.460757952	2.931150591	6.41E-07	1.28E-06
LINC01693	1.470479978	3.214234611	6.66E-07	1.32E-06
MAST4-IT1	1.971434413	2.699605874	7.18E-07	1.42E-06
LINC02422	1.285415004	4.381333223	8.53E-07	1.68E-06
LINC01093	-1.033380232	4.255995204	9.62E-07	1.89E-06
FABP6-AS1	1.603999841	2.816751051	9.67E-07	1.89E-06
LINC02726	2.290018212	2.863931605	1.00E-06	1.96E-06
DPP10-AS1	2.416283838	5.649750113	1.16E-06	2.27E-06
LINC02404	1.763097022	2.60721721	1.19E-06	2.32E-06
LINC01206	1.752651331	3.334823317	1.48E-06	2.88E-06
PTPRJ-AS1	1.376310936	2.884352598	1.58E-06	3.06E-06
ITPK1-AS1	1.146956469	3.498829286	1.63E-06	3.15E-06
LINC01143	1.205864363	5.075870705	1.70E-06	3.27E-06
SOX1-OT	3.188962559	4.014565264	1.70E-06	3.28E-06
LINC00404	2.986718125	3.368307324	1.76E-06	3.39E-06
HOTTIP	1.943157732	3.702628096	1.80E-06	3.47E-06
SLC1A2-AS1	1.467599542	2.76038494	1.81E-06	3.49E-06
LINC01194	3.318759348	4.177388157	1.90E-06	3.65E-06
GS1-594A7.3	2.286681801	4.764043412	2.09E-06	4.02E-06
C5orf17	1.720459724	6.145336313	2.14E-06	4.11E-06
LINC00393	2.664481686	5.629828528	2.34E-06	4.48E-06
LINC01198	-1.399272321	6.819949137	2.35E-06	4.50E-06
ZIM2-AS1	1.112859112	6.334633505	2.39E-06	4.57E-06
LINC01102	1.124959586	3.847848047	2.43E-06	4.65E-06
LINC02267	2.089495419	2.944027583	2.61E-06	4.98E-06
ABCC5-AS1	1.138302602	2.958430082	2.71E-06	5.17E-06
G2E3-AS1	2.316327785	3.358791022	2.92E-06	5.55E-06
IFNG-AS1	1.074655953	5.653650787	3.02E-06	5.75E-06
LINC01866	2.384069788	2.951020034	3.39E-06	6.43E-06
LINC01838	1.226615465	2.959471001	3.53E-06	6.68E-06

PCAT18	1.226229767	9.038647639	3.55E-06	6.71E-06
C21orf91-OT1	1.560200803	3.476625221	4.38E-06	8.22E-06
LINC01168	1.696013244	3.185209724	4.52E-06	8.49E-06
KIRREL3-AS1	2.046496565	5.777891653	4.73E-06	8.86E-06
LINC02115	1.537581292	5.116078798	4.75E-06	8.88E-06
LINC02306	1.703724936	6.352990274	4.96E-06	9.26E-06
SIRPG-AS1	1.064228194	3.905778166	5.01E-06	9.33E-06
CHN2-AS1	1.73150898	2.839587396	5.31E-06	9.88E-06
MTUS2-AS1	1.038480881	3.166988434	5.63E-06	1.04E-05
LINC02474	1.896892539	3.045399002	5.76E-06	1.07E-05
MIR2052HG	1.387263196	6.595151712	5.78E-06	1.07E-05
LINC02617	2.029634982	3.254746259	6.05E-06	1.12E-05
LINC01856	1.348193205	4.771411709	6.11E-06	1.13E-05
EGFR-AS1	-1.004805566	3.806766955	6.47E-06	1.19E-05
SCHLAP1	2.452756569	3.471001543	6.93E-06	1.27E-05
PHACTR2-AS1	1.170422097	3.13039008	7.48E-06	1.37E-05
NCF4-AS1	1.087374565	3.325660062	7.60E-06	1.39E-05
SHANK2-AS3	1.450496404	3.248208111	7.93E-06	1.45E-05
TMEM108-AS1	1.751706616	2.796999757	8.12E-06	1.48E-05
ASTN2-AS1	1.352043025	2.889512657	8.86E-06	1.61E-05
LINC01611	1.602285113	3.926325825	8.98E-06	1.64E-05
SPATA13-AS1	1.213408692	2.883096411	9.19E-06	1.67E-05
LINC02836	1.846310706	2.891480605	9.40E-06	1.71E-05
LINC02050	2.460228148	3.173225476	1.00E-05	1.82E-05
LINC01777	-1.225075315	2.896771136	1.12E-05	2.03E-05
LINC00501	1.24798977	3.482351186	1.13E-05	2.05E-05
KCNIP4-IT1	2.338557699	2.873760531	1.29E-05	2.31E-05
ARHGEF3-AS1	1.695106228	2.682667505	1.35E-05	2.42E-05
LINC02323	1.193662708	4.173278557	1.51E-05	2.70E-05
LINC02470	1.186220747	3.116562135	1.57E-05	2.80E-05
LINC02669	1.293104607	3.596247906	1.66E-05	2.95E-05
PCBP3-AS1	1.980229799	3.795092282	1.74E-05	3.09E-05
MIR4432HG	1.239439564	3.423690165	1.75E-05	3.10E-05
OSTM1-AS1	1.882873862	3.004461637	1.77E-05	3.13E-05
LINC00491	1.939109757	3.780317342	1.81E-05	3.20E-05
BTBD9-AS1	1.242225033	3.154618597	2.02E-05	3.57E-05
LINC01761	-1.07194483	3.711692824	2.41E-05	4.23E-05
LINC01954	1.134606776	3.700102446	2.72E-05	4.75E-05
LINC02233	1.945742062	4.244710515	2.81E-05	4.90E-05
LINC01115	1.480313927	4.811999608	2.94E-05	5.12E-05
ENOX1-AS1	1.032144791	3.024374101	3.11E-05	5.41E-05
LINC01539	1.235720777	3.203443715	3.12E-05	5.43E-05
LINC02189	-1.159593404	3.093126172	3.16E-05	5.48E-05
ROCR	-1.074663017	8.659099855	3.33E-05	5.77E-05
LINC01695	1.091501923	5.252109331	3.85E-05	6.65E-05
NFIA-AS1	1.095425991	3.577574459	4.09E-05	7.05E-05
LINC01910	1.324088259	4.424394426	4.95E-05	8.51E-05
LINC02735	1.673537374	2.836498656	5.61E-05	9.60E-05
FAM131B-AS1	1.307505524	3.266886247	6.33E-05	1.08E-04
FENDRR	1.212230975	4.579549789	6.50E-05	1.11E-04
LINC02864	1.758879864	5.108477843	6.69E-05	1.14E-04

LINC02555	1.219390832	2.973254263	7.13E-05	1.21E-04
MKX-AS1	1.856827239	5.207828677	7.31E-05	1.24E-04
LINC01249	-1.331094766	3.797824614	8.25E-05	1.39E-04
SNHG27	1.903720114	3.922286414	9.00E-05	1.51E-04
LINC01804	1.827906179	2.967871343	9.53E-05	1.60E-04
MACROD2-AS1	1.274875568	2.814440214	9.82E-05	1.64E-04
ADGRD1-AS1	2.01326534	3.479615325	1.05E-04	1.75E-04
NEUROG2-AS1	-1.056461911	4.932471505	1.19E-04	1.97E-04
LINC01324	1.905447791	2.941756795	1.30E-04	2.14E-04
LINC02477	1.518605747	3.276944011	1.40E-04	2.30E-04
LINC01748	1.488064492	4.320737845	1.41E-04	2.32E-04
LINC01456	2.17583014	4.628256557	1.43E-04	2.36E-04
MIR4500HG	1.337030979	3.842732789	1.52E-04	2.49E-04
LINC01840	1.28443788	4.082152176	1.53E-04	2.50E-04
CTD-2350J17.1	1.588154949	2.817235499	1.67E-04	2.73E-04
E2F3-IT1	1.251658193	2.747563004	1.75E-04	2.85E-04
LINC01111	2.151032322	3.001657298	1.77E-04	2.89E-04
F11-AS1	1.684769802	3.558873589	2.04E-04	3.30E-04
LPP-AS1	1.453076229	2.695053723	2.67E-04	4.27E-04
LINC01257	1.599048054	3.90075756	3.15E-04	5.03E-04
LINC02765	1.03285382	4.785768897	3.24E-04	5.16E-04
ATP13A4-AS1	-1.124997089	2.908451917	3.54E-04	5.60E-04
LINC02251	1.373609804	4.21804548	3.70E-04	5.85E-04
LINC01913	1.815630967	3.423202934	3.76E-04	5.95E-04
RC3H1-IT1	1.070260827	2.878584276	3.94E-04	6.21E-04
MYO16-AS1	1.444123403	2.94245405	3.97E-04	6.25E-04
FAM167A-AS1	1.107482753	2.964584188	4.01E-04	6.32E-04
LINC01950	1.576039192	3.482922901	4.18E-04	6.56E-04
LINC02562	1.432155866	6.92151844	4.42E-04	6.92E-04
LINC01602	1.061852724	2.807682194	4.67E-04	7.31E-04
LINC01507	1.361514838	3.058702295	4.78E-04	7.47E-04
KIF25-AS1	1.25915172	5.781903852	5.22E-04	8.12E-04
LINC02327	1.691124616	3.301865305	5.91E-04	9.15E-04
LINC02866	1.591548235	3.928108067	5.93E-04	9.18E-04
LINC01630	1.159752795	3.357588641	8.63E-04	1.32E-03
LINC01098	1.201611351	3.120921918	1.01E-03	1.54E-03
PDE4B-AS1	1.340166865	2.600702679	1.17E-03	1.76E-03
LINC00348	-1.081109418	2.974998135	1.17E-03	1.77E-03
PTPRT-AS1	1.193390372	2.650151978	1.18E-03	1.77E-03
LINC02041	1.261647783	3.545577345	1.20E-03	1.80E-03
RIC3-DT	1.603239416	3.381083724	1.23E-03	1.85E-03
JAKMIP2-AS1	1.025420128	3.260118582	1.34E-03	2.00E-03
LINC01036	1.896983938	3.261339527	1.36E-03	2.03E-03
KCND3-AS1	1.110044783	2.93887211	1.44E-03	2.15E-03
LINC01193	1.490422047	2.880047954	1.63E-03	2.41E-03
CACNA1C-IT3	1.207050431	2.74106523	1.75E-03	2.59E-03
EVX1-AS	1.005141568	2.91650523	1.85E-03	2.72E-03
PCAT14	1.083612035	6.824198242	1.85E-03	2.73E-03
CACNA1C-AS4	1.305629313	2.912949599	1.95E-03	2.86E-03
MAGI1-AS1	1.101735995	3.231192185	2.02E-03	2.97E-03
LINC00114	1.319182655	2.862549881	2.59E-03	3.77E-03

ARHGAP15-AS1	1.013420983	2.903436098	3.08E-03	4.46E-03
LINC01447	1.021292952	3.445766222	3.80E-03	5.44E-03
LINC02527	1.597757079	2.766997556	4.18E-03	5.97E-03
LINC01151	1.226835906	3.205144476	4.23E-03	6.04E-03
LINC01511	1.43617499	7.101942214	4.59E-03	6.54E-03
LINC01322	1.236894041	3.095724709	4.80E-03	6.82E-03
LINC00871	1.0787869	3.075856525	5.18E-03	7.34E-03
SOX5-AS1	1.292297169	2.609391076	5.28E-03	7.47E-03
LINC02302	1.066890901	5.882991385	5.96E-03	8.40E-03
LINC02434	1.432714372	3.1701919	7.47E-03	1.04E-02
LINC01793	1.14560017	3.03406095	8.07E-03	1.12E-02
MAGEA10-MAGEA5	1.032520627	2.950286225	8.11E-03	1.13E-02
LINC01749	1.158966805	3.374176266	1.24E-02	1.68E-02
LINC02476	1.239633376	2.775725838	1.71E-02	2.28E-02
