# Supplementary material for: Comprehensive analysis of cuproptosis-related long non-coding RNA signature and personalized therapeutic strategy of breast cancer patients
Source: Front Oncol. 2022 Dec 22;12:1081089. doi: 10.3389/fonc.2022.1081089 (PMC9815178; doi:10.3389/fonc.2022.1081089)
Supplement: Supplementary file 10 [file Table_3.pdf]

LncRNAs associated with BC patient prognosis

id	HR	HR.95L	HR.95H	p-value
TNFRSF14-AS1	0.727595231	0.620724977	0.852865342	8.72E-05
CAMTA1-AS1	0.788076757	0.644928029	0.962998889	1.99E-02
RERE-AS1	0.728733767	0.615479619	0.862827763	2.41E-04
LINC01778	0.861307232	0.748870417	0.990625521	3.64E-02
PIK3CD-AS2	0.823943115	0.711269435	0.954465669	9.85E-03
LINC01786	0.825163467	0.692861597	0.982728369	3.11E-02
PRKCZ-AS1	0.745563746	0.565961489	0.982160996	3.68E-02
NFIA-AS2	0.858941957	0.74386663	0.991819307	3.83E-02
PKN2-AS1	1.216031616	1.032355057	1.432387899	1.92E-02
VAV3-AS1	0.789626249	0.674258906	0.924733226	3.38E-03
LRIG2-DT	0.771133759	0.622587006	0.95512317	1.73E-02
LINC02609	0.791325189	0.65805482	0.951585697	1.29E-02
LINC00623	0.847325534	0.731373678	0.981660377	2.73E-02
LINC01765	0.71223177	0.562238895	0.902239419	4.91E-03
LINC00624	1.247325206	1.091481047	1.425421151	1.17E-03
LINC01633	1.209650524	1.06036248	1.379956776	4.62E-03
KIAA1614-AS1	1.253942653	1.036302824	1.517290256	2.00E-02
MIR205HG	0.915508984	0.84931706	0.986859607	2.11E-02
LINC01353	0.788855569	0.652058427	0.954351763	1.47E-02
LINC01341	0.85846128	0.743969729	0.990572251	3.66E-02
IPO9-AS1	0.76127065	0.596491607	0.971569417	2.84E-02
KCNH1-IT1	0.859941908	0.774063111	0.95534857	4.94E-03
LINC01344	0.886177369	0.796732473	0.985663765	2.60E-02
RABGAP1L-IT1	0.723572922	0.551560886	0.949229335	1.95E-02
LINC01036	1.164631052	1.0314773	1.314973666	1.39E-02
LINC00582	0.858421696	0.7470132	0.986445498	3.14E-02
PINK1-AS	1.473517462	1.054466352	2.059101939	2.32E-02
ATP1A1-AS1	0.696905082	0.520637716	0.932849617	1.52E-02
THBS3-AS1	0.794992151	0.654338704	0.965879775	2.09E-02
CHRM3-AS2	0.860452681	0.764190034	0.968841235	1.30E-02
LRRC8C-DT	0.809775534	0.685921878	0.955992855	1.27E-02
GORAB-AS1	1.201406723	1.020130863	1.414895055	2.79E-02
MRPL20-DT	1.339704546	1.084967099	1.654251334	6.57E-03
PEF1-AS1	0.759633064	0.630138819	0.915738524	3.94E-03
CNIH3-AS2	0.849538029	0.727182974	0.992480421	3.99E-02
LAMTOR5-AS1	1.478264779	1.0889552	2.006755427	1.22E-02
AKT3-IT1	0.746077563	0.575387889	0.967402582	2.71E-02
TTLL10-AS1	0.818528374	0.681497516	0.98311246	3.22E-02
ECE1-AS1	0.713458921	0.576412507	0.88308915	1.92E-03
TDRKH-AS1	1.344915987	1.056798392	1.711583804	1.60E-02
C2CD4D-AS1	1.285441644	1.058703515	1.560739334	1.12E-02
TFAP2E-AS1	0.79531588	0.655616128	0.964783082	2.01E-02
LINC01871	0.872571941	0.765963497	0.994018376	4.03E-02
ID2-AS1	1.226587909	1.005040509	1.496972395	4.45E-02
FLJ31356	1.234436767	1.044695393	1.458639658	1.34E-02
PPP1R21-DT	1.223949535	1.021558857	1.466437742	2.84E-02
RASGRP3-AS1	1.282440197	1.013829749	1.622217992	3.80E-02
LINC02245	0.834828617	0.710741015	0.980580554	2.79E-02
PGM5P4-AS1	0.70760161	0.560401011	0.893467407	3.65E-03

C2orf91	0.82452405	0.702339834	0.967964332	1.84E-02
FSIP2-AS1	0.82303021	0.695190881	0.974378038	2.37E-02
ANKRD44-AS1	0.763437431	0.657596171	0.886314029	3.93E-04
ANKRD44-IT1	0.737785091	0.589217169	0.92381361	8.03E-03
LINC01854	1.218401576	1.041738668	1.425023805	1.35E-02
KIAA2012-AS1	0.797140709	0.697260943	0.911327841	9.02E-04
C2orf49-DT	1.517069434	1.123057187	2.04931654	6.60E-03
LINC01889	1.134045044	1.005194146	1.279412707	4.09E-02
LINC01827	0.800858119	0.650561648	0.985876942	3.63E-02
LINC01117	0.901322238	0.817760915	0.993422113	3.64E-02
CATIP-AS1	0.783145942	0.657193521	0.933237391	6.29E-03
SNED1-AS1	1.196159906	1.001164966	1.429133628	4.85E-02
LINC01918	0.86379971	0.751797253	0.992488249	3.88E-02
LINC00471	1.322626351	1.020689615	1.713880927	3.44E-02
NIFK-AS1	0.586508595	0.363221676	0.947058929	2.91E-02
HDAC4-AS1	0.778132757	0.632078893	0.957935148	1.80E-02
BOLA3-AS1	1.199548325	1.005644437	1.430839899	4.31E-02
DGUOK-AS1	1.38497534	1.082164829	1.772518049	9.67E-03
HOXD-AS2	0.817494554	0.704505594	0.948604741	7.93E-03
TNS1-AS1	0.848027959	0.720111018	0.998667428	4.82E-02
MFF-DT	1.730916787	1.268908716	2.361141417	5.34E-04
LMCD1-AS1	1.290265895	1.037201592	1.605074744	2.21E-02
LINC02084	0.799739603	0.686581735	0.931547403	4.09E-03
SLC6A1-AS1	1.245050197	1.00883501	1.536574344	4.12E-02
SRGAP3-AS2	0.860982261	0.763287286	0.971181452	1.49E-02
FGD5-AS1	1.533338273	1.095804626	2.145570666	1.26E-02
LINC01267	0.814037125	0.672725096	0.985033032	3.44E-02
GOLGA4-AS1	1.536739186	1.177356422	2.005821926	1.57E-03
ESRG	0.76835689	0.620399229	0.951600651	1.58E-02
CYB561D2	0.825582924	0.705668703	0.965874159	1.67E-02
FOXP1-AS1	0.82090201	0.682170129	0.987847579	3.67E-02
LINC02018	0.781033992	0.638746957	0.955016834	1.60E-02
SEMA3F-AS1	0.687821923	0.521889563	0.906511705	7.89E-03
LINC01215	0.892941587	0.80623603	0.988971774	2.98E-02
SAMMSON	1.226739616	1.070983077	1.405148334	3.18E-03
SYNPR-AS1	0.885238397	0.806036837	0.972222339	1.08E-02
SIDT1-AS1	0.845763563	0.725817087	0.985532053	3.18E-02
LINC00881	0.791701644	0.648532924	0.96647598	2.17E-02
STAG1-DT	1.28352472	1.06883364	1.541339685	7.52E-03
NCK1-DT	1.489813357	1.081177924	2.052894153	1.48E-02
TBL1XR1-AS1	0.823581217	0.680056471	0.997396612	4.70E-02
ERICH6-AS1	0.744077101	0.58054082	0.953680971	1.96E-02
OPA1-AS1	1.260137125	1.041322692	1.524931307	1.75E-02
TPRG1-AS2	0.842464549	0.731626004	0.970094711	1.72E-02
SIAH2-AS1	0.803636255	0.705112688	0.915926265	1.05E-03
ACAP2-IT1	1.23835775	1.013198332	1.513553534	3.68E-02
LINC00885	1.163382359	1.015602323	1.332665831	2.90E-02
LINC02037	1.162199269	1.001678643	1.348443585	4.75E-02
ATP13A5-AS1	1.189645442	1.032762216	1.370360239	1.61E-02
PLCH1-AS1	1.244356614	1.009343785	1.534089183	4.07E-02
TIPARP-AS1	0.766067975	0.620972789	0.945065793	1.29E-02

ARIH2OS	0.714847622	0.516859313	0.988677401	4.25E-02
SCAANT1	0.770890603	0.632141393	0.940093986	1.02E-02
ZBTB20-AS1	0.826518513	0.694815842	0.983185487	3.14E-02
MME-AS1	0.736433245	0.544684845	0.995683888	4.68E-02
LINC02054	0.803572063	0.663155266	0.973720776	2.56E-02
WNT5A-AS1	0.867752151	0.771430966	0.976100039	1.81E-02
BSN-DT	1.150874337	1.005925166	1.316710012	4.08E-02
LINC02043	1.186803466	1.026455075	1.372200791	2.07E-02
SEMA3B-AS1	0.847767347	0.747791852	0.961108995	9.89E-03
FGF12-AS2	1.198813108	1.013475007	1.418044705	3.43E-02
ITPR1-DT	1.289153191	1.018474347	1.631770063	3.47E-02
EGOT	0.865175632	0.777372799	0.962895635	7.99E-03
ITGB5-AS1	0.800814024	0.665337317	0.963876644	1.88E-02
THUMPD3-AS1	1.513653951	1.113813238	2.057030932	8.08E-03
LINC02482	0.79682044	0.642473179	0.988247966	3.87E-02
LINC02265	0.79254478	0.672402198	0.934154038	5.57E-03
LINC02428	0.873095122	0.767484244	0.993238751	3.91E-02
FLJ20021	0.766387308	0.610535013	0.962024279	2.18E-02
WDFY3-AS2	1.285403276	1.02245605	1.615973206	3.15E-02
LNX1-AS2	1.268449221	1.065514886	1.510033737	7.51E-03
LINC02263	0.775886155	0.610587766	0.985934143	3.79E-02
UBA6-AS1	1.465690709	1.009627047	2.12776516	4.44E-02
SLC7A11-AS1	1.263897531	1.098399105	1.454332003	1.07E-03
LINC02466	1.147058778	1.002645359	1.312272409	4.57E-02
LINC02267	1.228310834	1.056202391	1.428464392	7.59E-03
HHIP-AS1	0.761387723	0.641426158	0.903784883	1.83E-03
LINC02275	1.153717851	1.016456112	1.309515349	2.69E-02
LINC01098	1.200798302	1.017623968	1.41694438	3.02E-02
LINC02477	1.185103806	1.01050814	1.389866123	3.68E-02
FAM160A1-DT	1.222809305	1.01714939	1.4700521	3.23E-02
UNC5C-AS1	0.869985569	0.759614699	0.996393159	4.42E-02
LINC02434	1.191685918	1.010490228	1.405372647	3.72E-02
USP46-DT	1.404142991	1.042876399	1.890557253	2.53E-02
SRD5A3-AS1	1.367712013	1.080076949	1.731947111	9.34E-03
LEF1-AS1	0.76744885	0.631608784	0.932504032	7.74E-03
MIR4458HG	0.866593274	0.760931793	0.986926699	3.09E-02
PURPL	1.177391317	1.062324929	1.304921192	1.86E-03
LINC01513	0.779396881	0.656318936	0.925555344	4.48E-03
LINC02223	1.245089921	1.058087679	1.465142202	8.29E-03
OSMR-AS1	0.837041133	0.701855027	0.998265783	4.78E-02
MEF2C-AS1	0.830306902	0.713873044	0.965731312	1.59E-02
LINC02062	0.784626804	0.627836499	0.980572527	3.30E-02
SNHG18	0.862962917	0.75089531	0.99175609	3.78E-02
LINC01950	1.192245334	1.035440186	1.372796764	1.45E-02
LINC02208	1.169769127	1.025533953	1.334290111	1.95E-02
ARHGAP26-AS1	0.76863513	0.635434635	0.929757258	6.73E-03
LINC01411	0.922814405	0.852767581	0.998614915	4.61E-02
LINC02227	0.79967912	0.646960047	0.98844851	3.87E-02
LINC01574	1.194237225	1.049530178	1.358896178	7.07E-03
LINC01863	0.851996267	0.766318354	0.947253365	3.06E-03
LINC02234	1.273517461	1.076135769	1.507102328	4.89E-03

PRR7-AS1	1.219536456	1.032260998	1.440787912	1.96E-02
PP7080	1.52560663	1.093867575	2.127748956	1.28E-02
SNHG4	1.1905444	1.023842757	1.384388334	2.34E-02
LINC02236	1.382700776	1.166434617	1.639064383	1.88E-04
ZNF474-AS1	0.794788239	0.663007502	0.952761985	1.30E-02
P4HA2-AS1	1.320866508	1.064161705	1.639495507	1.16E-02
FABP6-AS1	1.222235551	1.01944878	1.465360272	3.02E-02
LINC00491	1.177406149	1.050011361	1.320257372	5.19E-03
HCG18	1.699074256	1.140710162	2.530750953	9.12E-03
ELOVL2-AS1	0.892137489	0.824573621	0.965237401	4.50E-03
LINC01016	0.918761292	0.863854572	0.97715789	7.04E-03
BTBD9-AS1	1.259182872	1.021679442	1.55189724	3.07E-02
PTPRK-AS1	0.800327104	0.646394068	0.990917934	4.10E-02
LINC00472	0.892125737	0.799990472	0.994872261	4.01E-02
MLIP-IT1	1.208208987	1.031054823	1.415801491	1.94E-02
C6orf99	1.232557543	1.028298888	1.477389614	2.37E-02
STXBP5-AS1	1.603510558	1.290110571	1.993043209	2.08E-05
LINC01010	0.842299964	0.731109612	0.970400632	1.75E-02
TAGAP-AS1	1.436499747	1.056123518	1.953873281	2.10E-02
LINC02532	1.11554668	1.024269787	1.214957632	1.21E-02
LINC00602	1.149321855	1.005793368	1.31333211	4.09E-02
MPC1-DT	1.317052655	1.060933686	1.635001054	1.26E-02
SYNJ2-IT1	1.309481498	1.041956295	1.645694549	2.08E-02
AFDN-DT	1.224595227	1.031184051	1.454283033	2.09E-02
SDK1-AS1	1.217452629	1.019872043	1.453310652	2.94E-02
PRKAR1B-AS1	0.765336787	0.618796082	0.946580649	1.37E-02
LINC02888	1.111108043	1.015668347	1.215515958	2.15E-02
SEC61G-DT	1.251474863	1.023354547	1.530446448	2.89E-02
WEE2-AS1	0.686588151	0.538306217	0.875715855	2.45E-03
WDR86-AS1	0.835703211	0.732174441	0.953870851	7.82E-03
PRKAG2-AS1	0.777672411	0.651995189	0.92757491	5.18E-03
SUGCT-AS1	1.211672011	1.045134378	1.404746693	1.09E-02
PPP1R35-AS1	1.344423151	1.06853695	1.691540576	1.15E-02
ST7-AS1	0.720196911	0.566563902	0.915490007	7.34E-03
ST7-OT4	1.299695741	1.048062903	1.611743927	1.70E-02
CRPPA-AS1	1.319611991	1.074969413	1.619930563	8.03E-03
FKBP14-AS1	1.40198111	1.116860029	1.759890213	3.58E-03
ELN-AS1	0.879396217	0.785787147	0.984156726	2.52E-02
TP53TG1	0.745789	0.608907047	0.913441937	4.58E-03
MNX1-AS1	1.084362489	1.003322217	1.171948539	4.10E-02
LINC01456	1.121333433	1.029362225	1.221522062	8.72E-03
LINC01283	1.514378812	1.21521308	1.88719429	2.19E-04
ZNF674-AS1	1.363438708	1.024176064	1.815083534	3.37E-02
USP27X-DT	1.347551944	1.013595377	1.791539586	4.01E-02
LINC00630	1.591823587	1.220389879	2.076305592	6.06E-04
FTX	1.443731083	1.095990796	1.901803783	9.00E-03
FIRRE	1.136995246	1.019638776	1.26785899	2.09E-02
RAP2C-AS1	1.276125142	1.002567202	1.624325406	4.76E-02
LINC01278	1.541884549	1.110602495	2.140647056	9.69E-03
UXT-AS1	1.554290902	1.175057658	2.055916312	2.00E-03
TLR8-AS1	1.147418018	1.007318039	1.307003406	3.85E-02

MORF4L2-AS1	1.340067646	1.055588563	1.701213294	1.62E-02
ARMCX5-GPRASP2	1.493810769	1.074538319	2.07667849	1.70E-02
LINC00968	1.159508496	1.010140087	1.330963863	3.54E-02
LINC02605	0.844757665	0.733516967	0.972868447	1.92E-02
OTUD6B-AS1	1.8755804	1.424981143	2.468665536	7.25E-06
LINC01592	1.217086597	1.046310135	1.415736822	1.09E-02
MIR3150BHG	1.136157301	1.005018202	1.284407994	4.14E-02
RAD21-AS1	1.279572741	1.071529393	1.528008852	6.47E-03
ZNNT1	1.257541415	1.030053805	1.535269712	2.44E-02
FAM83A-AS1	1.135214415	1.037486712	1.242147734	5.76E-03
LINC00824	1.196892412	1.038102025	1.379971728	1.33E-02
BAALC-AS1	1.195401557	1.019102735	1.402199046	2.84E-02
WASHC5-AS1	1.246402231	1.019261332	1.524161148	3.19E-02
MAL2-AS1	1.210796567	1.025664038	1.429345548	2.39E-02
ASAP1-IT2	0.743044369	0.620152405	0.890289113	1.28E-03
NCRNA00250	1.310582681	1.092494813	1.572206058	3.58E-03
RHPN1-AS1	1.261278304	1.02444749	1.552859443	2.87E-02
LINC00536	1.176625327	1.062461929	1.303055782	1.79E-03
LNCOC1	1.248424306	1.096910958	1.420865783	7.76E-04
MAFA-AS1	0.815265558	0.710936548	0.934904714	3.46E-03
MIR31HG	0.829394126	0.717308321	0.958994335	1.16E-02
PGM5-AS1	0.845521185	0.728447573	0.98141047	2.73E-02
SMC5-DT	1.421967939	1.071546812	1.886985055	1.47E-02
LINC01508	1.093309798	1.001897055	1.193063009	4.52E-02
BARX1-DT	1.143008991	1.010239715	1.293227274	3.39E-02
UNQ6494	0.836990498	0.716704943	0.97746374	2.46E-02
DBH-AS1	0.784886006	0.67509885	0.912527168	1.63E-03
BNC2-AS1	0.790510997	0.671999896	0.929922222	4.56E-03
C9orf163	1.36200311	1.091640692	1.699325139	6.21E-03
LINC01235	1.209840782	1.088001405	1.345324291	4.36E-04
PAPPA-AS2	0.720172433	0.568630329	0.912101073	6.47E-03
PCAT7	1.150674774	1.019001742	1.299362289	2.36E-02
NAV2-AS6	0.862001475	0.752302249	0.987696826	3.25E-02
LINC02726	1.20428778	1.012812452	1.431962112	3.54E-02
LINC00294	1.428012402	1.093328523	1.86514792	8.93E-03
WT1-AS	1.093051103	1.011002702	1.18175818	2.54E-02
ZNRD2-AS1	1.41476904	1.008282059	1.985130468	4.47E-02
RSF1-IT1	1.291620426	1.108805762	1.50457671	1.01E-03
LINC01395	1.186715539	1.026956331	1.371327803	2.03E-02
SENCR	0.781114597	0.633138521	0.963675393	2.12E-02
GSEC	1.280764526	1.01874575	1.610173854	3.41E-02
IRAG1-AS1	0.832081918	0.694602073	0.996772606	4.60E-02
LINC02752	0.829798223	0.706066025	0.975213458	2.35E-02
KRTAP5-AS1	1.131725043	1.005072287	1.274337766	4.10E-02
LINC01001	1.420532298	1.095193641	1.842516186	8.17E-03
CENATAC-DT	1.432880198	1.134041558	1.810467745	2.58E-03
LINC02685	1.207354497	1.024392235	1.422994857	2.46E-02
ADARB2-AS1	1.192720831	1.093781038	1.300610389	6.64E-05
MANCR	1.239726293	1.092158863	1.407232348	8.90E-04
ST8SIA6-AS1	1.100119906	1.01923932	1.18741868	1.43E-02
RPP38-DT	1.285394247	1.030274329	1.603687798	2.61E-02

UNC5B-AS1	0.824902476	0.70637861	0.963313562	1.50E-02
DLG5-AS1	0.706746153	0.561534469	0.889509288	3.10E-03
ZNF503-AS1	1.144767773	1.031798837	1.270105381	1.08E-02
LNCAROD	1.141273611	1.029225722	1.265519727	1.22E-02
ANK3-DT	0.815675772	0.710037036	0.937031353	3.99E-03
ENTPD1-AS1	1.314952672	1.013174448	1.706616794	3.96E-02
NUTM2A-AS1	1.506773199	1.063046764	2.135715522	2.13E-02
PDCD4-AS1	0.651835559	0.507011361	0.838027763	8.43E-04
PITRM1-AS1	1.339350205	1.022553634	1.754293283	3.38E-02
SFTPD-AS1	0.748006915	0.588724268	0.950384374	1.75E-02
NUTM2B-AS1	1.485257684	1.091638785	2.020806167	1.18E-02
LINC02652	1.187261817	1.004044503	1.403912493	4.47E-02
WAC-AS1	2.189963071	1.496833031	3.204056934	5.40E-05
LINC02617	1.194315649	1.015082118	1.405196529	3.23E-02
LINC02449	1.209267963	1.007276633	1.451765045	4.16E-02
HOTAIR	1.094357988	1.015293236	1.179579814	1.84E-02
LINC02408	1.241985305	1.039165269	1.484390929	1.72E-02
GIHCG	0.763470745	0.589089278	0.989472394	4.14E-02
LINC01234	1.210548204	1.109995666	1.320209618	1.57E-05
CAPS2-AS1	0.674473802	0.499331773	0.911047392	1.03E-02
ADGRD1-AS1	1.22645867	1.084028037	1.38760329	1.19E-03
LINC02823	0.816573666	0.702509348	0.949158263	8.30E-03
USP30-AS1	0.850179128	0.740878923	0.975604147	2.08E-02
LINC02463	1.195188455	1.028847267	1.388423227	1.97E-02
PCED1B-AS1	0.840645664	0.711884343	0.992696552	4.07E-02
LINC00987	0.803226097	0.66451617	0.970890087	2.35E-02
LINC01465	0.719528018	0.541200801	0.956614563	2.35E-02
DDN-AS1	1.295298655	1.05695268	1.587392357	1.26E-02
CCND2-AS1	0.783357435	0.624240855	0.983032217	3.51E-02
LINC01559	1.200867502	1.068773035	1.349288118	2.08E-03
LRRK2-DT	0.802536478	0.682362619	0.943874679	7.86E-03
LLPH-DT	1.168826487	1.002305931	1.363012343	4.67E-02
CACNA1C-IT3	1.562432288	1.183165624	2.063273816	1.66E-03
LINC00544	0.781910546	0.634295684	0.963878703	2.12E-02
LINC00398	0.854786055	0.732352306	0.997688125	4.67E-02
LINC00393	1.128163077	1.035639489	1.228952682	5.74E-03
LINC00377	0.648461435	0.479837378	0.876343218	4.82E-03
LINC01232	1.263705167	1.043409372	1.530512176	1.66E-02
LINC01055	0.837764057	0.730166919	0.961216671	1.16E-02
PRECSIT	1.198576663	1.02836871	1.396956173	2.05E-02
LINC00348	1.212339393	1.030327071	1.426505082	2.03E-02
DOCK9-DT	0.788959188	0.623497493	0.998330558	4.84E-02
FGF14-AS2	0.803372877	0.685206843	0.941917008	6.99E-03
SLC25A30-AS1	0.755090647	0.640720623	0.889875969	8.02E-04
LINC00645	1.258713311	1.043253621	1.518671172	1.63E-02
LINC00519	0.793635496	0.667441186	0.943689591	8.90E-03
LINC02588	1.12020754	1.018024105	1.232647565	2.00E-02
LINC02318	1.378419077	1.125277181	1.688507671	1.93E-03
DIO3OS	0.821828891	0.730160013	0.925006457	1.15E-03
FAM181A-AS1	0.724374171	0.53601189	0.978929664	3.59E-02
PSMA3-AS1	0.635309994	0.409672468	0.985223125	4.27E-02

ACTN1-AS1	0.745971449	0.6161268	0.903179999	2.67E-03
LINC01269	1.148268506	1.019914684	1.29277535	2.23E-02
C14orf178	0.775474844	0.602078666	0.998808409	4.89E-02
HIF1A-AS3	1.198632422	1.017672402	1.41177031	3.00E-02
PWRN1	1.231189227	1.013820743	1.495162654	3.59E-02
THBS1-AS1	0.930079596	0.867847959	0.99677374	4.02E-02
TPM1-AS	0.820782877	0.711176435	0.947281854	6.92E-03
EWSAT1	0.899773084	0.812538601	0.996373098	4.24E-02
C15orf54	1.186644122	1.010552855	1.393419717	3.68E-02
LINC00926	0.684866648	0.571291247	0.821021377	4.28E-05
MIR4713HG	1.136287336	1.015759105	1.271117241	2.55E-02
GPR176-DT	1.218031781	1.007795396	1.472125617	4.13E-02
VPS33B-DT	1.493180604	1.176419508	1.895232356	9.82E-04
LINC00923	0.7956201	0.650929531	0.972472922	2.56E-02
OIP5-AS1	1.419452762	1.033856907	1.94886365	3.03E-02
SPATA41	0.75924068	0.647788046	0.88986886	6.73E-04
LINC02130	0.878244063	0.787616847	0.979299309	1.95E-02
ITFG1-AS1	1.226109869	1.011280299	1.486576386	3.81E-02
LINC02133	1.190516805	1.003206905	1.41279955	4.59E-02
CRNDE	1.439880049	1.099972265	1.884824393	7.97E-03
LINC00922	0.895451399	0.802044652	0.99973637	4.95E-02
LINC02141	1.183618554	1.00368414	1.39581052	4.51E-02
LINC01572	1.344519824	1.110239778	1.628237065	2.44E-03
LINC01569	0.777318233	0.616126763	0.980680716	3.36E-02
CORO1A-AS1	0.834429131	0.723180651	0.962791212	1.32E-02
CENPN-AS1	1.267802317	1.03313691	1.555769325	2.31E-02
FAM157C	1.207385167	1.019306897	1.430166857	2.92E-02
CARHSP1-DT	0.766498541	0.620659604	0.946605853	1.35E-02
KIF1C-AS1	0.764783927	0.613458536	0.953437633	1.71E-02
LINC02091	0.742810437	0.622260232	0.886714782	9.99E-04
ZFP3-DT	1.223892314	1.029020545	1.455668114	2.24E-02
LINC01563	0.834169644	0.705676079	0.986060059	3.36E-02
RARA-AS1	0.743939773	0.595015659	0.930137513	9.45E-03
SP2-AS1	1.459429205	1.152976625	1.847334594	1.67E-03
LINC00671	0.74804768	0.603252825	0.927596703	8.18E-03
LINC00511	1.143573818	1.004878141	1.301412604	4.20E-02
CACNA1G-AS1	0.843500918	0.716996838	0.992324877	4.01E-02
LINC01978	1.165987094	1.022478337	1.329637856	2.19E-02
RNF213-AS1	0.734033315	0.60032442	0.897522887	2.58E-03
ERVE-1	1.130013051	1.011271083	1.262697526	3.09E-02
MAPT-AS1	0.827233774	0.764898945	0.894648531	2.08E-06
MAPT-IT1	0.850256153	0.785019342	0.920914285	6.81E-05
LINC00482	1.139674878	1.012510048	1.282810802	3.03E-02
TMEM105	1.122768309	1.003944745	1.255655436	4.25E-02
PIK3R5-DT	1.23779243	1.057683129	1.448571937	7.84E-03
LINC00670	1.342030771	1.065812381	1.689834556	1.23E-02
SNHG16	1.304418366	1.024388722	1.660997664	3.11E-02
LINC01973	0.777764002	0.63437082	0.953569779	1.56E-02
FMNL1-DT	0.811276617	0.695842116	0.945860755	7.57E-03
LHX1-DT	1.119826034	1.028728894	1.218990108	8.94E-03
BAIAP2-DT	0.733700406	0.567516959	0.948546608	1.81E-02

RUNDC3A-AS1	1.166321071	1.028991348	1.321978892	1.61E-02
HID1-AS1	1.27964465	1.045302269	1.566523368	1.69E-02
ARHGAP28-AS1	0.814556477	0.683481667	0.97076818	2.19E-02
LINC01925	1.182084802	1.015812313	1.37557348	3.06E-02
DLGAP1-AS1	0.701406386	0.518540272	0.948761254	2.14E-02
PPP4R1-AS1	0.777292299	0.637765241	0.947344381	1.26E-02
DLGAP1-AS5	0.90510656	0.833175536	0.983247648	1.83E-02
AQP4-AS1	0.835478872	0.726244204	0.961143569	1.19E-02
TTC39C-AS1	0.798270503	0.682957548	0.933053303	4.65E-03
SNHG22	0.71112965	0.578770199	0.873758497	1.18E-03
NDUFV2-AS1	0.555253434	0.400723855	0.769373651	4.07E-04
LINC01901	1.182885111	1.017144117	1.375633169	2.92E-02
LINC01539	0.758346059	0.607373679	0.946845023	1.46E-02
LINC00668	1.077254865	1.001306343	1.158964041	4.60E-02
GAPLINC	0.770027065	0.605423865	0.979382736	3.32E-02
PCAT18	0.870430379	0.807783243	0.937936075	2.71E-04
LIVAR	1.323696112	1.176577531	1.489210315	3.09E-06
GREB1L-DT	0.67679553	0.564369182	0.811618005	2.53E-05
KDSR-DT	0.676889913	0.534625267	0.85701141	1.19E-03
LINC00654	1.282846968	1.095388417	1.502386112	2.00E-03
STK4-AS1	1.529885698	1.217512859	1.922402899	2.63E-04
CFAP61-AS1	1.267096983	1.089329004	1.47387498	2.15E-03
LINC01275	1.249723733	1.054684655	1.480830694	1.00E-02
C20orf197	0.748359444	0.614173988	0.911861896	4.04E-03
LINC00266-1	1.336392329	1.094811867	1.631279777	4.37E-03
LAMA5-AS1	0.842868458	0.731306002	0.97145003	1.83E-02
ZNF337-AS1	1.343043711	1.034825755	1.743062927	2.66E-02
LINC01711	0.795799466	0.68644274	0.922577737	2.46E-03
LINC00659	1.144549088	1.022191397	1.28155316	1.93E-02
NORAD	1.384034048	1.019355938	1.879177013	3.73E-02
DPP9-AS1	0.793639814	0.655262956	0.961238763	1.81E-02
KLF2-DT	0.704424976	0.534519395	0.928337778	1.28E-02
LINC00662	1.443331541	1.070662321	1.945717055	1.60E-02
DPY19L3-DT	1.230199124	1.004606819	1.506449941	4.50E-02
NAPA-AS1	0.729227228	0.531801481	0.999945221	5.00E-02
IGFL2-AS1	1.148510731	1.028893592	1.282034322	1.36E-02
